# Photoabsorption and Photoionization Cross-Sections and Asymmetry Parameters of Pyrrole in the Vacuum-Ultraviolet Energy Range

**DOI:** 10.1021/acsphyschemau.4c00101

**Published:** 2025-02-24

**Authors:** Mariana B. M. S. Medeiros, Josenilton N. Sousa, Manuela S. Arruda, Milton M. Fujimoto, Manoel G. P. Homem, Helder K. Tanaka, Bruno Credidio, Ricardo R. T. Marinho, Frederico V. Prudente

**Affiliations:** † Instituto de Física, 28111 Universidade Federal da Bahia, 40170-115 Salvador, BA, Brasil; ‡ Instituto Federal Baiano, Campus Guanambi, 46430-000 Guanambi, BA, Brasil; § Departamento de Física, 28122 Universidade Federal do Paraná, 81531-980 Curitiba, PR, Brasil; ∥ Departamento de Química, 67828 Universidade Federal de São Carlos, 13565-905 São Carlos, SP, Brasil; ⊥ 169704 Instituto Federal da Bahia, Campus Porto Seguro, 45810-000 Porto Seguro, BA, Brasil; # Instituto de Física, 28127 Universidade de Brasilia, 70910-970 Brasília, DF, Brasil

**Keywords:** photoabsorption cross-sections, photoionization cross-sections, photoionization quantum yields, neutral-decay cross-sections, asymmetric parameters, five-membered heteroaromatic compounds

## Abstract

We carried out a joint experimental and theoretical investigation of the interaction of vacuum-ultraviolet radiation with pyrrole molecules in the gas phase. A double-ion chamber spectrometer was used to measure the absolute photoabsorption cross-sections and the photoionization quantum yields from the ionization threshold to 21.5 eV using synchrotron radiation. Moreover, photoionization and neutral-decay cross-sections on an absolute scale were derived directly from the measured data. In addition, theoretical results of the photoionization cross-section (PICS) and asymmetric parameters were obtained from the ionization threshold to 50.0 eV considering all valence orbitals. These calculations were performed using the Padé approximant technique to solve the Lippmann–Schwinger equation at the static-exchange (SE) and static-exchange-polarization (SEP) levels of approximation. Comparisons are also made with the experimental and theoretical data available in the literature. We report, for the first time, experimental ionization efficiency, neutral decay cross-sections, theoretical photoionization cross-sections for each valence orbital, and several asymmetry parameters. In general, the results are consistent and in good agreement with the available data.

## Graphical abstract

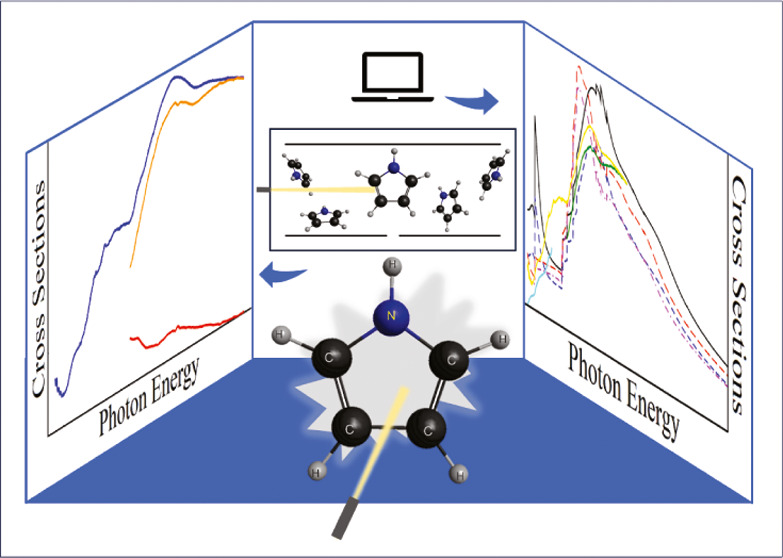

## 1 Introduction

Pyrrole, one of the simplest five-membered heteroaromatic compounds, has five p orbitals and six π electrons delocalized in the planar ring that contribute to its aromaticity^1^ and is considered an important building block in many biological molecules^2–4^ and technological systems.^5,6^ Specifically, pyrrole is a highly active compound with diverse biological activities. Pyrrole-containing analogs are potential sources of biologically active compounds found in natural products. Its combination with different pharmacophores in a ring system produces even more active compounds. Marketed drugs with pyrrole ring systems exhibit various biological properties, including antipsychotic, b-adrenergic antagonistic, anxiolytic, and anticancer effects, as well as antibacterial, antifungal, antiprotozoal, and antimalarial activities.^7^

On the other hand, pyrrole has an interesting role in astrochemistry. It has already been detected in meteorites,^8^ and there is a great expectation of its detection in the interstellar medium (ISM),^9^ due to the abundance of larger polycyclic aromatic nitrogen heterocycles (PANHs),^10^ among others. However, several attempts to detect pyrrole in ISM have failed.^11–13^ One of the reasons for this nondetection may be associated with the aforementioned tendency for a large portion of pyrrole to adhere strongly to the surface of interstellar dust grains, thus reducing their abundance in the gas phase.^9^ A process of hydrogenization^14^ or dehydrogenization^15^ of the pyrrole may also be occurring, influencing its detection. For example, the molecule C_4_H_4_N may be present in Titan’s atmosphere.^15^ Another possibility would be that much of the chemically active nitrogen would not be available in the form of NH or NH_2_, whose reaction with butadiene could be an important route of forming pyrrole.^16^ Pyrrole and other aromatic rings with π character orbitals exhibit charge transfer properties, which are central to various biological processes and technological applications, including solar energy harvesting and molecular electronics.^17^

Another aspect to be added is that the knowledge of the total absolute photoionization cross-section (PICS) is essential for the quantitative analysis of different molecules, especially heterocyclic compounds. For example, comparing the photoionization cross-section between different radicals and also theoretical and experimental results allows evaluating the concentration of specific compounds in a complex mixture,^18–20^ such as in combustion processes.^21^ The photoionization cross-section study combined with mass spectra of the products of the photoionization process also contributes to the determination of ionization and dissociation rates in the interstellar medium.^22,23^ Furthermore, these cross-sections provide essential information about the photochemical reactivity of heterocycles, contributing to the elucidation of reaction mechanisms and chemical processes.^24^

In the case of pyrrole molecule, investigations related to photoabsorption spectra, using theoretical methods^25–29^ and experimental techniques,^30–33^ were and have been explored investigating some characteristics such as excitations below and above the first ionization threshold. In the work of Palmer et al.,^34^ a study of the photoabsorption cross-section via monochromatized synchrotron radiation was carried out, for photon energies starting below the first ionization potential (IP), that is, in an energy range from 5.0 to 10.8 eV, where a LiF filter was used (IP = 11.3 eV). Rennie et al.^35^ determined the absolute photoabsorption cross-section in a wider energy range, i.e., from 8.2 to 35.0 eV, with a technique called the double ionization chamber,^36^ although different from the one used in this work. Furthermore, Rennie et al. also report mass spectrometry measurements in which some pyrrole ion fragments were identified. However, to our knowledge, only one experimental study reports the photoionization cross-section of pyrrole, from its ionization thresholds to 11.5 eV.^37^ Specifically, Xie et al.^37^ carried out the measurements using the photoionization mass spectrometry technique with tunable synchrotron vacuum-ultraviolet (VUV) light as an ionization source.

It is noteworthy that, in addition to studies on the interaction of pyrrole with photons, other research has also focused on its interactions with particles in the gas phase, including electrons^38–41^ and ions.^42–44^ These investigations enhance the understanding of the dynamic processes involving pyrrole, such as excitation, ionization, and charge transfer, complementing the knowledge gained from photoabsorption studies.

Following the trend of some recent papers^45–47^ of our research group, the present work aims to study theoretically and experimentally the photostability of pyrrole molecules in the vacuum-ultraviolet region. In theoretical studies, we determined the photoionization cross-section and the asymmetry parameter in the energy range between 8.2 and 50 eV, using the ePolyScat^48,49^ computational package. In the experimental approach, using the double ionization chamber technique,^50^ we determined the absolute photoabsorption cross-section in the energy range of 8.1–21.5 eV, and the absolute photoionization cross-section (from the ionization quantum efficiency) in the energy range between 13.5 and 21.5 eV. Knowledge of these quantities helps to understand the mechanisms of interaction between pyrrole and VUV radiation, providing essential data for studies of astrochemistry and chemical reactivity of nitrogenous heterocyclic systems. With these theoretical and experimental data, we hope to contribute to identifying molecules in astrophysical environments and understanding the photochemical processes that can influence the formation of complex organic molecules.

The structure of this paper is as follows: Section 2 describes the experimental and theoretical methods employed to calculate and measure the properties of interest. The results are presented in Section 3, and in Section 4, we present our concluding remarks.

## 2 Methodology

### 2.1 Experimental Section

The experimental results on the photostability of pyrrole were recorded using a synchrotron radiation source at the Brazilian National Synchrotron Light Laboratory (LNLS). In particular, we use the TGM beamline which operated in the vacuum-ultraviolet and soft X-ray regions^51,52^ and it had three toroidal grating monochromators, designed to provide photons with energies ranging from 7.3 to 310.0 eV, with a resolution of *E*/Δ*E* ≈ 500. An important feature of this line was associated with the fact that photons with energies greater than 14.00 and 21.56 eV are eliminated through the use of neon + krypton and neon gas filters,^53,54^ respectively, ensuring that there is no contamination of higher-order harmonics. Emphasizing that the absolute value of the photon energy was calibrated by observing the energy cutoff at the ionization potential of Ne, and the energy resolution of the beam was evaluated at 50 meV at this edge.

At the end of this beamline, an experimental station could be set up consisting of an ionization chamber where the double ionization chamber technique, proposed by Samson,^55^ was used. By obtaining the absolute values of the ionic currents *i*_1_ and *i*_2_ produced in the chamber containing the vapor sample (pyrrole), it was possible to determine the photoabsorption cross-section (σ_a_(*E*)) above the first ionization energy, using the equation

1 $$\sigma_{a}\left(E\right)=\frac{1}{\mathrm{nL}}\;\ln\left(\frac{i_{1}}{i_{2}}\right)$$

where *E* is the photon energy; *n* is the molecular density of the sample in the chamber of length *L*, determined by measuring the pressure in the ideal gas regime. With the knowledge of *I*_0_(*E*), obtained by combining the flows of Xe and Ar gases, taking into account η_Ar_ = η_Xe_ = 1, it is possible to determine the values of photoionization quantum efficiency η

2 $$\eta \left(E\right)=\frac{i_{1}^{2}}{eI_{0}\left(i_{1}-i_{2}\right)}$$

where *e* is the electron charge. From this information, the absolute values of the photoionization (σ_i_(*E*)) and neutral decay (σ_n_(*E*)) cross-sections are obtained in the following ways

3 $$\sigma_{i}\left(E\right)=\eta \left(E\right)\sigma_{a}\left(E\right)$$

and

4 $$\sigma_{n}\left(E\right)=\sigma_{a}\left(E\right)-\sigma_{i}\left(E\right)$$

respectively, in the energy range between 13.5 and 21.5 eV. It is important to highlight that this technique has already been used in previous studies and more details can be found in the refs 46,47,56,57 . Specifically, the results were determined by performing cycles of current measurements in photon energy steps of 0.01 eV, within a pressure and voltage range of 30–35 mTorr and 13–22 V, respectively.

The pyrrole is a colorless volatile liquid at room temperature, and the sample was purchased from Sigma-Aldrich with a purity of better than 98.0%. All measurements, at room temperature, were performed with the sample in the gas phase. This was obtained from the saturated vapor above the liquid sample in a vial attached to the gas handling system. Freeze–pump–thaw cycles were performed to eliminate atmospheric air and other volatile contaminants.

Random errors, such as sample pressure fluctuations during energy scans, contribute significantly to the overall experimental error in the measured data. The uncertainty due to pressure fluctuations was evaluated as being less than 3.5%. Furthermore, fluctuations of ionic currents measured during various scans were slightly greater than 1 and 2.5% for *i*_1_ and *i*_2_, respectively. The overall experimental uncertainty, given by the square root of the sum of the squares of the individual uncertainties, is 4% for the absolute σ_a_ results. The associated uncertainties of *I*_0_(*E*) and η­(*E*), recorded in the energy range between 13.5 and 21.5 eV, were evaluated at 3.5 and 10%, respectively, generating an overall uncertainty of around 11% for the absolute results in σ_i_ and σ_n_ (see the Supporting Information of ref 46 for more details of the error analysis).

### 2.2 Theoretical Calculations

#### 2.2.1 Theory

The direct photoionization process can be denoted by

5 $$h\nu +M\to M^{+}+e^{-}$$

The probability of this transition is related to the square of the dipole transition integral, which links the initial state of neutral molecule M to the final states of ion M^+^ and the photoelectron. In the transition integral, the electric dipole operator is written in two equivalent forms, the first in the length form (*L*)­

6 $$I_{k,\mathrm{n̂}}^{\left(L\right)}=k^{1/2}\left\langle \psi_{i}\right|r\cdot \mathrm{n̂}\left|\psi_{k}^{\left(-\right)}\right\rangle$$

and also in the velocity form (*V*)­

7 $$I_{k,\mathrm{n̂}}^{\left(V\right)}=\frac{k^{1/2}}{E}\left\langle \psi_{i}\right|\nabla \cdot \mathrm{n̂}\left|\psi_{k}^{\left(-\right)}\right\rangle$$

where ψ_i_ is the initial state represented by the ground state wave function of the neutral molecule; ψ_**k**_^(−)^ is an antisymmetrized product between the molecular ion wave function and the photoelectron; **k** is the momentum vector of the ejected electron; ^(−)^ means that ψ_**k**_^(−)^ satisfies the incoming spherical waves boundary condition; *E* is the photon energy, and *n̂* is the direction of polarization of light, which is admitted as linearly polarized. These matrices lead to a selection rule for dipole-allowed transitions.

The differential cross-section in the usual molecular gas phase experiment is averaged over all target orientations and given by

8 $$\frac{d\sigma^{\left(L,V\right)}}{d\Omega_{k}}=\frac{\sigma^{\left(L,V\right)}}{4\pi }\left[1+\beta_{k}^{\left(L,V\right)}P_{2}\left(\cos\theta \right)\right]$$

where σ^(*L,V*)^ is the integral photoionization cross-section, obtained in the length (*L*) or velocity (*V*) form of the dipole operator, β_**k**_ is the asymmetry parameter, and *P*_2_(cos θ) is the Legendre polynomial of order 2. θ is the angle between the light polarization vector and the momentum vector direction of the photoelectron.

The asymmetry parameter is written as^58,59^

9 $$\beta_{k}^{\left(L,V\right)}=\frac{3}{5}\;\frac{1}{\sum_{p\mu \mathrm{lhv}}{\left|{I_{\mathrm{lhv}}^{p\mu }}^{\left(L,V\right)}\right|}^{2}}\times \sum_{\begin{array}{l} p\mu lvmm_{v}\\ p^{\prime }\mu^{\prime }l^{\prime }h^{\prime }v^{\prime }m^{\prime }m\prime_{v} \end{array}}{\left(-1\right)}^{m\prime -m_{v}}A\times B\times C$$

where

$$\begin{array}{l} A=\left({I_{\mathrm{lhv}}^{p\mu }}^{\left(L,V\right)}\right){\left({I_{l\prime h\prime v\prime }^{p\prime \mu \prime }}^{\left(L,V\right)}\right)}^{*}\\ B=\left(b_{\mathrm{lhm}}^{p\mu }\right){\left(b_{l\prime h\prime m\prime }^{p\prime \mu \prime }\right)}^{*}\left(b_{1h_{v}m_{v}}^{p_{v}\mu_{v}}\right){\left(b_{1h\prime_{v}m\prime_{v}}^{p\prime_{v}\mu \prime_{v}}\right)}^{*}\\ C={\left[\left(2l+1\right)\left(2l^{\prime }+1\right)\right]}^{1/2}\left(11-{m^{\prime }}_{v}m_{v}|2M^{\prime }\right)\times \left(1100|20\right)\left(l^{\prime }l00|20\right)\left(l^{\prime }l-m^{\prime }m|2-M^{\prime }\right) \end{array}$$ 10

where *m* and *m*_*v*_ are projected components of *l* and *v*, respectively; *M* is the component of total angular momentum *L*; *b*′*s* are the expansion coefficients for the corresponding symmetry and (*l*′*lm*′*m*|*L*′*M*′) are the usual Clebsch–Gordan coefficients.

To compare our calculations with experimental measurements in the gas phase, the PICS in the laboratory frame is calculated by averaging all possible molecular orientations relative to the polarization direction of the light beam, *n̂*, and integrating over all photoelectron directions, *k̂*. The integral photoionization cross-section is given by

11 $$\sigma^{\left(L,V\right)}\left(E\right)=\frac{\pi^{2}E}{c}\int d\Omega_{\mathrm{k̂}}\int d\Omega_{\mathrm{n̂}}{\left|I_{k,\mathrm{n̂}}^{\left(L,V\right)}\right|}^{2}$$

where *c* is the speed of light. The approach in eq 11 is known as the integrated target angular distribution (ITAD).

In our calculation methodology which will be detailed further up, the wave functions above are expanded in partial-waves and single-center, in terms of the symmetry-adapted functions,^60^ χ_*lh*_^*pμ*^, the dipole transition integral results in

12 $$I_{k,\mathrm{n̂}}^{\left(L,V\right)}={\left[\frac{4\pi }{E}\right]}^{1/2}\sum_{p\mu \mathrm{lhv}}^{\max}I_{\mathrm{lhv}}^{p\mu \left(L,V\right)}\chi_{\mathrm{lh}}^{p\mu }\left(\mathrm{k̂}\right)\chi_{1v}^{p_{v}\mu_{v}}\left(\mathrm{n̂}\right)$$

where *p* is one of the irreducible representations of the molecular point group, μ is a component of the representation *p*, and *h* runs over all possible χ_*lh*_^*pμ*^ which belongs to the same irreducible representation (*pμ*) with the same value of angular momentum *l*, due to dipole terms *l* = 1, *h* = *v*, corresponding to a photon with one unit of angular momentum and projections *v* = −1, 0, 1, which are correlated with irreducible representations (*p*_*v*_μ_*v*_).

The integral photoionization cross-section in partial waves is

13 $$\sigma^{\left(L,V\right)}\left(E\right)=\frac{4\pi^{2}E}{3c}\sum_{p\mu \mathrm{lhv}}^{\max}{\left|{I_{\mathrm{lhv}}^{p\mu }}^{\left(L,V\right)}\right|}^{2}$$

#### 2.2.2 Molecular Systems

The pyrrole molecule has a planar geometry formed by a five-atom aromatic ring (see Figure 1) and belongs to the *C*_2v_ point group symmetry with the following irreducible representations: A_1_, B_1_, A_2_, and B_2_. The wave function of the ground state of the neutral molecule was obtained in the Hartree–Fock level with aug-cc-pVTZ basis set,^61^ taken from the Basis Set Exchange software.^62^ It was the largest basis set that we managed to obtain accurate results in a reasonable computational time. The nuclear geometry used for pyrrole, optimized at the HF/6-311G level, is shown in Table 1 and closely matches the experimental geometry from Alparone et al.^63^ With this geometry, we achieved excellent agreement with the molecular properties reported in the literature. The electronic structure calculation was done with Gaussian 03, revision D.01 ^64^ and the calculated total energy was −208.886223 au for the ground state of neutral pyrrole which is in accordance with −208.87676 au,^40^ using the DZP basis set. The calculated dipole moment of 1.892 D is in excellent agreement with 1.895 D from de Oliveira et al.^38^ The electronic configuration for the ground state is represented by

$$\begin{array}{ll} {\left(1a_{1}\right)}^{2}{\left(1b_{2}\right)}^{2}{\left(2a_{1}\right)}^{2}{\left(3a_{1}\right)}^{2}{\left(2b_{2}\right)}^{2} & \left(\mathrm{core}\right)\\ {\left(4a_{1}\right)}^{2}{\left(5a_{1}\right)}^{2}{\left(3b_{2}\right)}^{2}{\left(6a_{1}\right)}^{2}{\left(4b_{2}\right)}^{2}{\left(7a_{1}\right)}^{2}{\left(8a_{1}\right)}^{2}{\left(5b_{2}\right)}^{2}{\left(1b_{1}\right)}^{2}{\left(6b_{2}\right)}^{2}{\left(9a_{1}\right)}^{2}{\left(2b_{1}\right)}^{2}{\left(1a_{2}\right)}^{2} & \left(\mathrm{valence}\right) \end{array}$$

**Figure 1:**
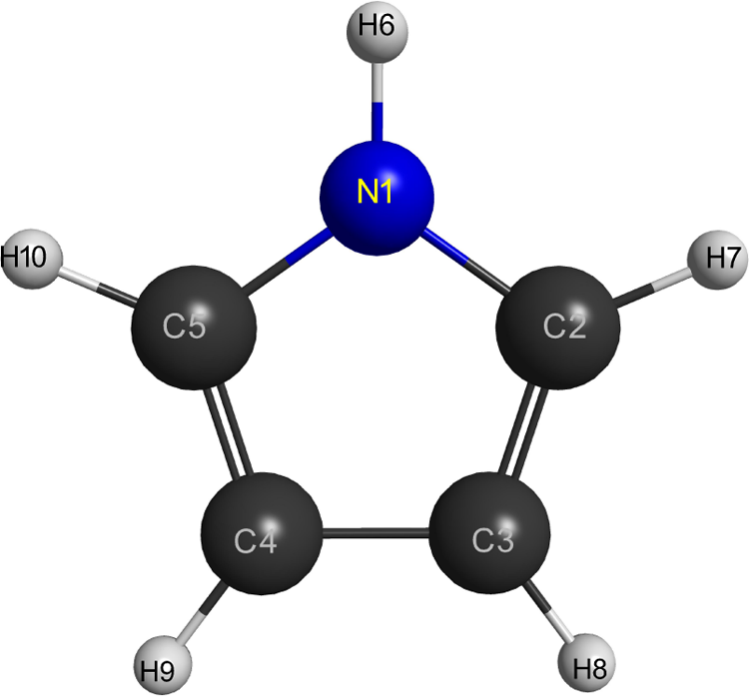
Molecular structure of the pyrrole molecule.

**Table 1:** Nuclear Geometry for the Pyrrole Molecule

distance	*r* (Å)	angle	θ (deg)
N1–C2	1.37203	C3–C2–N1	108.00
C2–C3	1.36119	C4–C3–C2	107.31
C3–C4	1.42958	H6–N1–C2	125.30
N1–H6	0.98629	H7–C2–N1	121.44
C2–H7	1.06472	H8–C3–C2	126.10
C3–H8	1.06569		

The ion molecular orbitals are represented in the frozen-core approximation, which describes the cation wave function using the orbitals of the neutral molecule in the same nuclear geometry. In this approximation, an electron is removed from the respective ionized orbital.

The ionization potential of each valence orbital is an input parameter for calculating the photoionization cross-sections and asymmetry parameters. In this work, we employ the best estimate of the experimental results for vertical ionization potentials presented by Chong.^65^ These values are shown in Table 2.

**Table 2:** Experimental Vertical Ionization Potential for Valence Orbitals from Pyrrole Molecule^65^

orbitals	1a_2_	2b_1_	9a_1_	6b_2_	1b_1_	5b_2_	8a_1_	7a_1_	4b_2_	6a_1_	3b_2_	5a_1_	4a_1_
IP (eV)	8.2	9.2	12.8	12.9	13.5	14.3	14.8	17.4	18.0	18.8	22.3	23.8	29.5

#### 2.2.3 Photoelectron Wave Function

The photoelectron wave functions used in eqs 6 and 7 were calculated using the ePolyScat.E package^49^ which considers an iterative scheme using the Schwinger Variational Principle combined with Padé approximants and is described in detail elsewhere.^48^ The ψ_**k**_ is obtained by solving the one-electron Schrödinger equation given, in atomic units, by

14 $$\left[-\frac{1}{2}\nabla^{2}+V^{\left(N-1\right)}\left(r,R\right)-\frac{k^{2}}{2}\right]\psi_{k}\left(r\right)=0$$

where $-\frac{1}{2}\nabla^{2}$ represents the kinetic energy of the photoelectron; *V* ^*N*–1^ is the interaction potential between the continuum electron and the molecular ion given by

15 $$V^{\left(N-1\right)}=V_{\mathrm{st}}+V_{\mathrm{ex}}+V_{\mathrm{cp}}$$

where *V*_st_ is the static potential; the coulomb interaction due to the net charge of the ion and photoelectron is also embedded in *V*_st_; *V*_ex_ is the exchange term, calculated exactly by the molecular wave function in the HF level; *V*_cp_ is the correlation-polarization potential model from Perdew and Zunger.^66^ The latter contribution is a parameter-free potential derived from the free-electron gas local density approach for the correlation part. The asymptotic polarization potential of *V*_cp_ is given by

16 $$\lim_{r\to \infty }V_{\mathrm{pol}}=-\frac{\alpha }{r^{4}}$$

where α is the ion polarizability. The first crossing between them determines the inner and outer regions of the (*V*_cp_) potential. The value of polarizability employed in this work is 54.87 au,^63^ which is from the neutral molecule. Although appropriately the ion polarizability should be used in *V*_pol_, Fófano et al.^67^ recently showed that using the polarizability of the neutral molecule instead of the ion does not significantly affect the ionization cross-section and asymmetry parameters in the photoionization study of ethylene oxide. The rationale is that for the long-range interaction, the Coulomb interaction is dominant compared to polarization effects when a photoelectron moves away from the ion at relatively higher energies.

The ePolyScat.E, version 3, solves the Lippman–Schwinger equation instead of eq 14. In this method, the wave functions and potentials are expanded in partial waves and single-center in the center of mass of the system. The cutoff parameters used for the partial-wave expansions were up to *R*_max_ = 10.0 Å for the maximum *R* value on the radial numerical grid for the scattering wave function and interaction potentials; *l*_max_ = 30 for molecular and continuum wave functions; *l*_max*A*_ = 15 for maximum *l* included at larger *r*; and *l*_max*K*_ = 10 for *K*-matrix, which is used to obtain the photoelectron wave function. The *K*-matrix, which would be obtained from the asymptotic form of the scattering wave function, is used to compute the dipole matrix element.^68^ With these values, the photoionization cross-sections can be considered to be converged to less than 0.5% for all calculated results.

## 3 Results and Discussion

### 3.1 Absorption and Ionization Cross-Sections

Our experimental result of the absorption cross-section, σ_a_, measured in the range of 8.1–21.5 eV is presented in Figure 2, along with the measured quantum efficiency (η) and the results of the photoionization (σ_i_) and neutral decay cross-sections (σ_n_), which are shown starting at 13.5 eV. The values of (σ_a_), (η), (σ_i_) and σ_n_ for some energies are listed in Table S1 of the Supporting Information.

**Figure 2:**
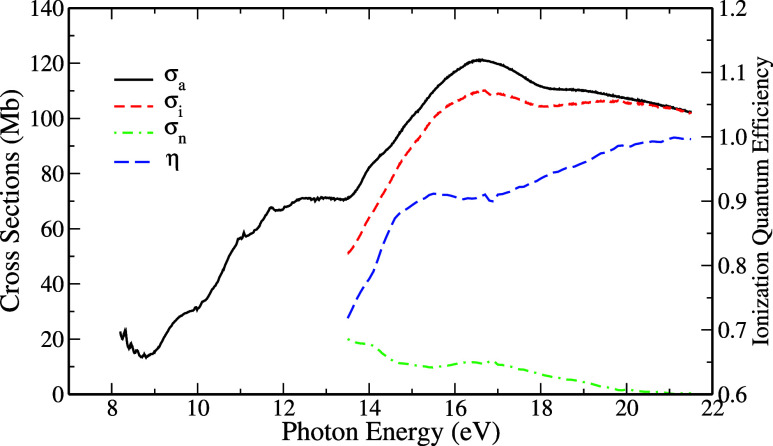
Present quantum ionization efficiency (η, long-dashed blue line) and photoabsorption (σ_a_, black solid line) and photoionization (σ_i_, short-dashed red line) and neutral-decay (σ_n_, short-dash-dotted green line) cross-sections.

We determined the first ionization potential to be 8.20 ± 0.05 eV from our experimental measurement of the currents *i*_1_ and *i*_2_ produced in the experimental chamber and the analysis of their derivatives. This result shows good agreement with previously recorded results: 8.21,^34^ 8.209,^35^ 8.3,^69^ 8.1,^70^ and 8.25 ^65^ eV and is associated with the opening of the first ionization channel (orbital 1a_2_). After this energy value, a positive inflection (associated with maxima of the derivative of σ_a_) is observed at the beginning of the photoabsorption spectrum, at 9.2 ± 0.1 eV, which is associated with the opening of the ionization channel (orbital 2b_1_). These two energy values agree with the peaks present in the photoelectron spectra of Sell and Kuppermann^71^ and Derrick et al.,^72^ which correspond to the two valence orbitals with the lowest ionization potential values.

It is possible to see in Figure 2, a very sharp increase in the quantum efficiency of ionization up to 14.6 eV, which means a significant increase in direct ionization processes. This growth in η­(*E*) is associated with the opening of three ionization channels, associated with states 6b_2_, 8a_1_, and 5b_2_: peaks are visible in the PES spectra of Sell and Kuppermann^71^ and Derrick et al.^72^ At 14.7 ± 0.1 eV, the change in the slope in η­(*E*) is noticeable. The quantum ionization efficiency reaches a plateau in the energy range between 15.5 and 17.2 eV. This stabilization in η also corresponds to a small increase in the neutral decay cross-section (σ_n_). Then, there is another increase in η, corroborated by the appearance of three peaks in the photoelectron spectrum (associated with orbitals 7a_1_, 4b_2_, and 6a_1_).

Still, in Figure 2, the values of σ_a_ and σ_i_ are closer to each other for photons with higher energies. In particular, from 21.0 eV and above, σ_a_ and σ_i_ coincide, while σ_n_ and η take on the values 0 and 1, respectively. This behavior means that all absorbed photons necessarily promote the ionization of the molecule.

In order to compare our results with previous studies, Figure 3 presents the experimental photoabsorption cross-section of the current study, together with the experimental σ_a_ results recorded by Palmer et al.^34^ and by Rennie et al.^35^ The figure also shows the theoretical results obtained by Tenorio et al.^73^ It is noted that below the first ionization potential (8.2 eV) there are three energy bands in the spectra from Palmer et al.^34^ (6.0, 6.8, and 7.5 eV) and Tenorio et al.^73^ (5.3, 6.1, and 7.4 eV) which are associated with the absorption of photons by the molecule that does not produce direct ionization. According to Palmer et al.,^34^ in these bands, there are Rydberg states associated with the autoionization of valence orbitals 1a_2_ e 2b_1_.

**Figure 3:**
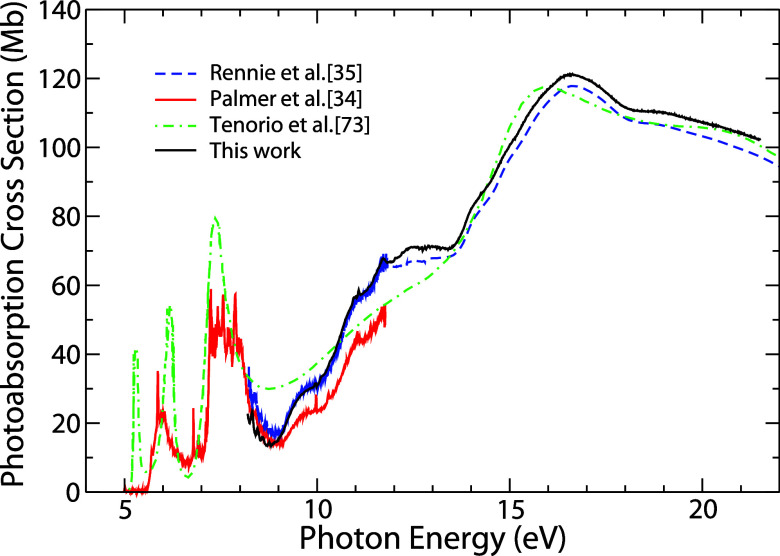
Photoabsorption cross-section (σ_a_) for pyrrole in the VUV energy range. (Black solid line) Present results; (short-dashed blue line) data of Rennie et al. obtained from ref 35; (solid red line) data of Palmer et al. obtained from ref 34; and (short-dash-dot green line) theoretical results of Tenorio et al. obtained from ref 73.

In Figure 3, we observe that our experimental σ_a_ result coincides with the result of Palmer et al.^34^ right off after the first ionization potential, besides presenting the same profile and structure as the data obtained by Rennie et al.^35^ However, in the range of 8.2–9.0 eV, our experimental spectrum is about 5 and 15% smaller than the experimental data of Palmer et al.^34^ and Rennie et al.,^35^ respectively. Above this energy (9.0 eV), our experimental result becomes closer to that of Rennie et al.^35^ and coincides up to about 12 eV, after that our values are about 6% higher than theirs, for higher energies. It indicates that our σ_a_, within error bars ca. 4%, is in good agreement with literature data.

To verify the accuracy of the measured absorption cross-section, the S(−2) sum rule analysis of the dipole oscillator strength distribution (d*f*/d*E* = 9.112 × 10^–3^σ_a_) was performed to determine the static polarizability value due to the pyrrole’s electric dipole. Since our result only covers the energy range from 8.2 to 21.5 eV, we completed the estimate of σ_a_ with the experimental data of Palmer et al.,^34^ for energies below 8.2 eV, and with the asymptotic decay of the photoabsorption spectrum, for values above 21.5 eV using the polynomial function σ_a_ = *A* · *E* ^–2^ + *B* · *E* ^–3^ + *C* · *E* ^–4^, where *A*, *B*, and *C* are constants to be determined and *E* is the photon energy. Figure 4 represents the (d*f*/d*E*)/*E* ^2^ distribution, resulting from the combination of the photoabsorption spectrum from ref 34 (blue line) and the asymptotic decay (red line). With the curve presented in this figure, the value obtained for the static polarizability of the pyrrole’s electric dipole was 50.3 au for the neutral molecule. This value is in good agreement with 54.87 au^63^ and also with the previously determined using double refraction electrical measurements: 53.56 ^74^ and 55.80 au.^75^

**Figure 4:**
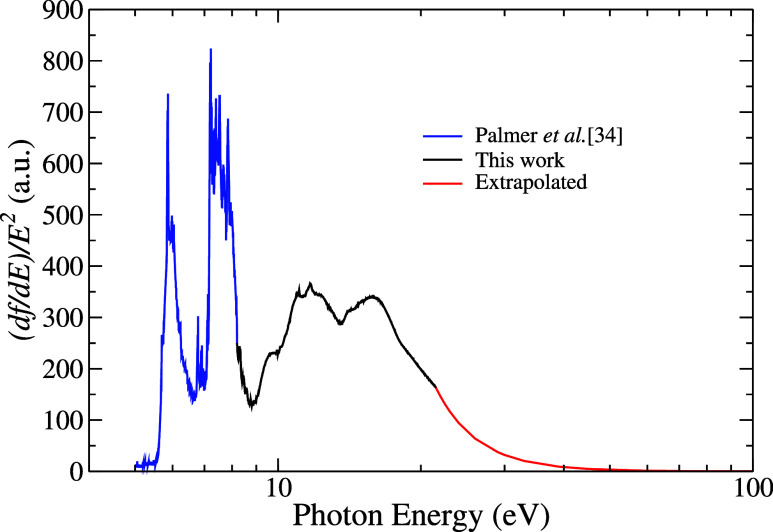
Combination of the high-resolution absorption recorded by Palmer et al. obtained from ref 34 for low energies of pyrrole molecule (solid blue line). Present study: the photoabsorption cross-sections (solid black line) and the extrapolation for high energies (solid red line).

For a more detailed analysis of the experimental ionization cross-section, we calculated the individual contributions (partial ionization cross-sections) of each of the 13 outermost pyrrole valence orbitals, 1a_2_, 2b_1_, 9a_1_, 6b_2_, 1b_1_, 5b_2_, 8a_1_, 7a_1_, 4b_2_, 6a_1_, 3b_2_, 5a_1_, and 4a_1_, in the photon energy range from 8.2 to 50.0 eV, with steps of 0.25 eV. Calculations are presented in four different approaches: the dipole operator in length (*L*) and velocity (*V*) form, and also at the static-exchange (SE) (without) and static-exchange-polarization (SEP) (with polarization) levels. The results of the 12 outermost orbitals are presented, for the first time, in Figure 5, in four levels of calculations: static-exchange­(SE)-*L*/*V* and static-exchange-polarization (SEP)-*L*/*V*. Each cross-section includes all allowed dipole transitions from each target molecular orbital to continuum orbitals with appropriate symmetry. The choice of representing only the first 12 orbitals in this figure comes from the fact that the partial cross-section of the last orbital is relatively small. However, the numerical values for all 13 valence orbitals are listed in Tables S2–S14 of the Supporting Information.

**Figure 5:**
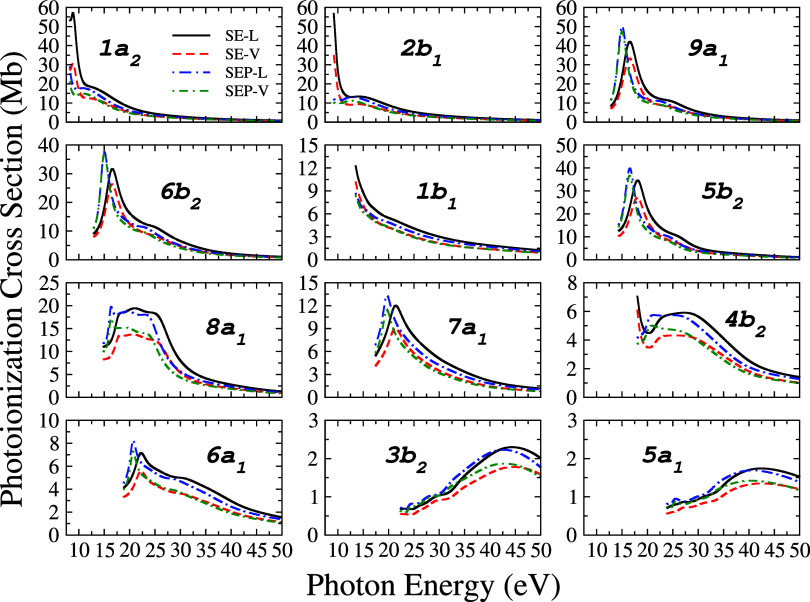
Partial photoionization cross-sections of pyrrole, of the 12 valence orbitals, in the length form (*L*): solid black line, without polarization (SE); short-dash-dot blue line, with polarization (SEP) and in the velocity form (*V*): short-dashed red line, without polarization (SE); short-dash-dot green line, with polarization (SEP).

It is observed from the results at the SE level that the 1a_2_ orbital has the largest cross-section in length form, with a value of 57.1 Mb for the energy value of 8.8 eV, while the largest cross-section in velocity form is for the orbital 2b_1_, with the value of 35.0 Mb for the energy value of 9.2 eV. With the inclusion of the polarization effect (SEP level), orbital 9a_1_ has the highest cross-section values, both for length (50.1 Mb) and velocity (47.2 Mb), for the energy of 15 eV. It is also noted that at high energies (from 30 eV), the values of the cross-sections decrease and there is a convergence between the length and velocity curves at both levels of polarization. As expected, the values of the cross-sections for the innermost orbitals, 3b_2_ and 5a_1_, are relatively small over the entire energy range of the incident photon. The results for 1b_1_, 4b_2_, 3b_2_, and 5a_1_ do not show clearly observable resonance peaks in this energy range, and consequently, the cross-sections in the SE and SEP approximations are practically the same. These results show that the polarization effect basically affects the resonance position, which can be seen in the peaks of the cross-section, shifting them to lower values of the incident photon energy by about 2–3 eV. The behavior of moving the location of resonance peaks, when polarization is introduced is also observed in theoretical results for the electron scattering cross-section by the pyrrole molecule, employing the Schwinger multichannel method implemented with pseudopotentials.^38^ The shift in the resonance position arises from the stabilization of the ion-photoelectron complex, which occurs due to the distortion of the ion’s electronic cloud in response to the electric field generated by the ejected electron. We hope that these four-level results can serve as a guide for experimentalists in future measurements.

In Figure 6, the sum of the photoionization cross-sections of all valence orbitals (1a_2_ + 2b_1_ + 9a_1_ + 6b_2_ + 1b_1_ + 5b_2_ + 8a_1_+ 7a_1_ + 4b_2_ + 6a_1_ + 3b_2_ + 5a_1_ + 4a_1_) is shown, in length and velocity approaches, at the SE and SEP levels, compared with the experimental photoionization and photoabsorption cross-sections measured by this work. Additionally, the experimental σ_i_ from the ionization thresholds to 11.5 eV obtained by Xie et al.,^37^ which has an uncertainty lower than ±25%, is included for comparison. A first comment on our experimental photoionization cross-section σ_i_, which was obtained from 13.5 eV, seems to join smoothly with the data set measured by Xie et al.^37^ if we consider the similar trend of experimental photoabsorption cross-section in the energy gap between 11.5 and 13.5 eV. This reinforces the quality of our measurement of the absolute photoionization cross-section of pyrrole.

**Figure 6:**
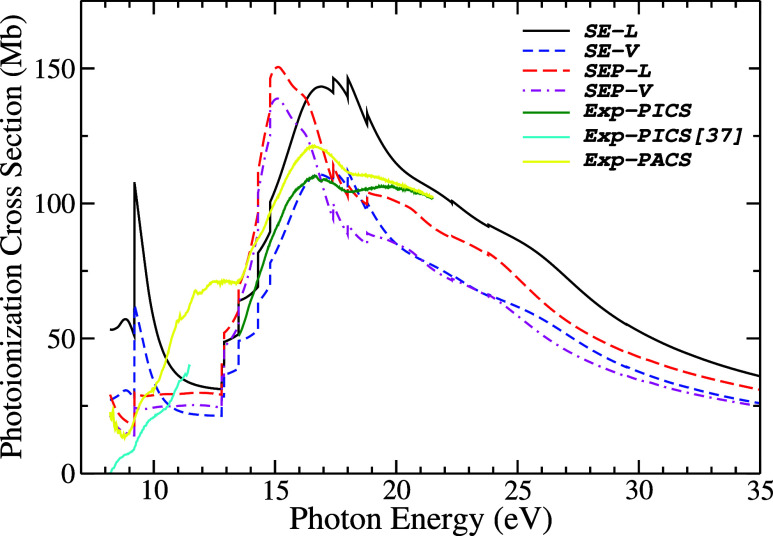
Pyrrole photoionization cross-sections (PICS). Present results: theory, sum of 13 outermost orbitals: in the length form (*L*): solid black line, static-exchange (SE); long-dashed red line, static-exchange-polarization (SEP); in the velocity form (*V*): short-dashed blue line at the SE level; short-dash-dot magenta line at the SEP level. Experimental results: solid green line, photoionization cross-section, and solid yellow line, photoabsorption cross-section (PACS). The experimental σ_i_ data of Xie et al. obtained from ref 37, solid cyan line, is included for comparison.

In general, all theoretical curves present similar profiles, except around the first ionization threshold, where for the curves without polarization (SE), a cusp in the cross-section is noted, which is due to the orbitals 1a_2_ e 2b_1_, as can be seen in Figure 5. However, when the polarization effect is included, this structure disappears and gives way to a slight minimum at around 9 eV, which can also be observed in the experimental photoabsorption cross-section. This happens because, when polarization is included, the structures due to the 1a_2_ and 2b_1_ orbitals are moved below the ionization threshold, as seen in Figure 5. The experimental photoionization cross-section of Xie et al. shows a slope change in the same energy region as well.

In Figure 6, for the SE calculation level, we observe that the first peak of σ_i_ is around 9.2 eV and has, for the length form, the value of 107.9 Mb (contributions of 52.9% from the 2b_1_ orbital and 47.1% from the 1a_2_) and, for the velocity form, 60.8 Mb (contributions of 56.02% from the 2b_1_ orbital and 43.9% from the 1a_2_ orbital). The second peak of σ_i_ is located around 17.5 eV and has, for the length form, the value of 146.4 Mb (major contributions from orbitals 9a_1_ with 24.6%, 5b_2_ with 21.5%, 6b_2_ with 19.0% and 8a_1_ with 11.1%) and, for the velocity form, 112.3 Mb (major contributions from orbitals 9a_1_ with 25.1%, 5b_2_ with 22.2%, 6b_2_ with 20.3% and 8a_1_ with 10.8%). For SEP calculations, σ_i_ was attenuated around the ionization threshold. Thus, σ_i_ has a peak located around 15.1 eV and has, for the length form, the value of 150.5 Mb (largest contributions from orbitals 9a_1_ with 33.0%, 6b_2_ with 24.4%, 5b_2_ with 13.2% and 1a_2_ with 8.0%) and, for the velocity form, 138.8 Mb (major contributions from orbitals 9a_1_ with 33.8%, 6b_2_ with 26.5%, 5b_2_ with 14.1% and 8a_1_ with 7.3%).

Analyzing our measured experimental curves of σ_a_ and our calculated and measured σ_i_, we observed no peak in the region just after the first ionization threshold. It suggests that the peak in this region in our SE results reflects nonphysical behavior. On the other hand, it is noticeable that the SE results present a second maximum peak around 17.5 eV, in good agreement with the experimental results of σ_a_ and σ_i_ curves, while the peak observed in the SEP results is shifted by about 2.5 eV to lower energies. Another point regarding the σ_a_ and theoretical photoionization σ_i_ cross-sections: the difference in behavior between these cross-sections in the range of 10.5–13.5 eV can partly be attributed to the processes leading to excitation and autoionization.

In general, in the region of 13.5–21.5 eV, the theoretical photoionization cross-sections obtained with the static-exchange approximation agree better with the experimental σ_i_ than those obtained with the static-exchange-polarization approximation; this even allows us to evaluate the main ionization channels responsible for the maximum in the experimental σ_i_. However, for energies around and slightly above the first ionization threshold, the results obtained at the SEP level present a physically more acceptable behavior compared to the experimental data. In both cases, the results obtained using the *V* form are more quantitatively reasonable compared with the measured σ_a_. It suggests that including the polarization-correlation term through the PZ potential, used here, is important in the region close to the ionization threshold (mainly to treat the two outermost orbitals 1a_2_ and 2b_1_). When the photoelectron moves away from the ion with low kinetic energies (near 2 eV above the ionization threshold), the *V*_cp_ potential from Perdew and Zunger represents adequately the polarization effects. However, when the photoelectron moves away relatively faster (near 7 eV above the threshold) or when dealing with more innermost orbitals, this potential does not simulate the correlation and polarization effect properly if we compare it to the experimental σ_i_. Despite the fact that this potential works well in many cases of scattering by neutral molecules,^38^ these results show that for photoionization studies this potential may not work adequately for energies around 7 eV above the ionization threshold. This is somewhat expected since in a long-range description the Coulomb interaction is more important than polarization-correlation effects when the electron is moving away relatively faster, which makes it almost unnecessary to include polarization effects in photoionization studies, in this case.

### 3.2 Asymmetry Parameters

An accurate determination of the asymmetry parameter helps in the correct assignment of the intensity and in the angular distribution of a photoelectron spectrum.^76^ For this purpose, we determined the asymmetry parameters (β) at the SE and SEP level, in the length and velocity forms, for the dipole-allowed transitions involving the electron ionization from each of the 13 valence orbitals of the pyrrole molecule. Results for 12 outermost valence orbitals are presented in Figure 7 in the photon energy range from 8.2 to 50.0 eV, while numerical values for selected energies are presented for 13 outermost orbitals in Tables S14–S27 of the Supporting Information. It is observed that there is a difference between the calculations of β at the SE and SEP levels for energies up to about 5 eV above the ionization threshold. This suggests that including the correlation and polarization effect slightly affects the description of the photoelectron angular distribution at lower energies. Other than that there are no significant differences between the length and velocity forms for β.

**Figure 7:**
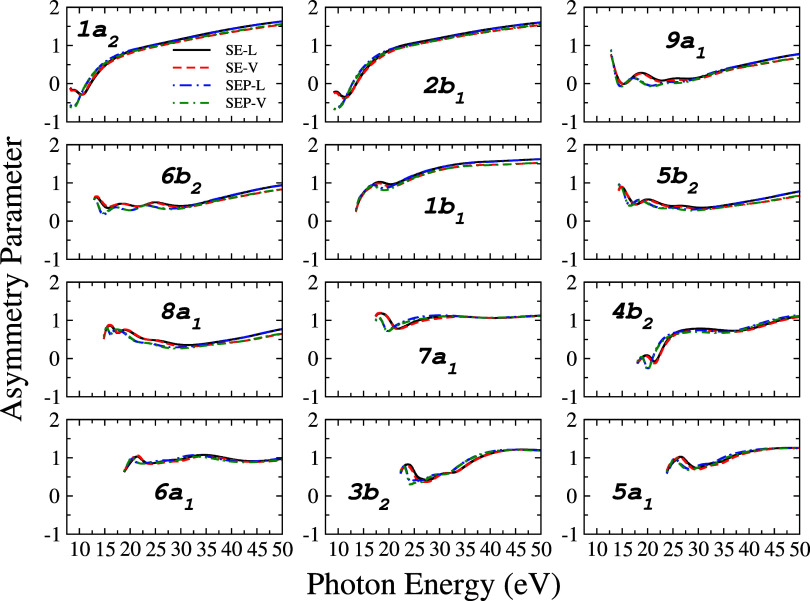
Asymmetry parameters of the 12 outermost valence orbitals of pyrrole, in the length form (*L*): solid black line, without polarization (SE); long-dash-dot blue line, with polarization (SEP) and in the velocity form (*V*): short-dashed red line, without polarization (SE); short-dash-dot green line, with polarization (SEP).

Previous studies indicate that the behavior of β reflects the σ or π character of the orbital from which ionization occurs.^1,77,78^ Thus, it can be employed to characterize the nature of molecular orbitals.^79^ In particular, the asymmetry parameter for π orbitals increases more quickly as a function of the energy of the incident photon until it reaches a plateau region at relatively high energies. On the other hand, σ orbitals tend to oscillate or their values vary less compared to π orbitals; the value of β for ionization of π orbitals is usually 0.2–1.0 units larger than for the ionization of σ orbitals.^71^ In Figure 7 we see that the profiles of the asymmetry parameters for orbitals 1a_2_, 2b_1_, and 1b_1_ grow rapidly with energy and finally reach a plateau, an average asymptotic value of approximately 1.62, indicating that they may be of character π. Orbitals 9a_1_, 6b_2_, 5b_2_, 8a_1_, 7a_1_, 4b_2_, 6a_1_, 3b_2_, 5a_1_, and 4a_1_ show small variations and fluctuations in the β values, most characteristic of σ orbitals. Furthermore, unlike π orbitals, which have a well-defined behavior, it is possible to observe from our results that the β associated with σ orbitals have an oscillating behavior, with differences in magnitude and shape in their profiles.

Another interesting aspect about the asymmetry parameter is that the presence of a minimum in the β of a given orbital is related to the resonance position in the partial photoionization cross-section for that orbital. Carlson et al.^80^ observed this behavior in their experimental measurements. For example, orbital 9a_1_ presents a resonance peak around 17.5 eV at the SE level and 15.0 eV at the SEP level. It can be seen that β for this orbital presents a valley around these energies, with a value around zero (at both calculation levels). As β is related to the angular distribution of the photoelectron ejected in the photoionization process, this result suggests that for incident photon energies around the resonance the photoelectron is preferentially ejected isotropically.

Finally, in Figure 8, we compare the β determined here with theoretical results^81^ and experimental data^69^ found in the literature for the outermost valence orbitals 1a_2_ and 2b_1_. The curves present the same profile, and our results are in better agreement with the theoretical β determined by Ponzi et al.^81^ It is possible to observe that the theoretical results, including ours, present smaller values than the experimental data above approximately 20 eV. The reason for the discrepancy is not clear to us at the moment. A similar discrepancy was observed for benzene, and a possible explanation for the discrepancy was pointed out as the opening of the double ionization channel,^82^ for pyrrole the double ionization occurs at 24.2 eV.^83^ This is not taken into account in our calculations.

**Figure 8:**
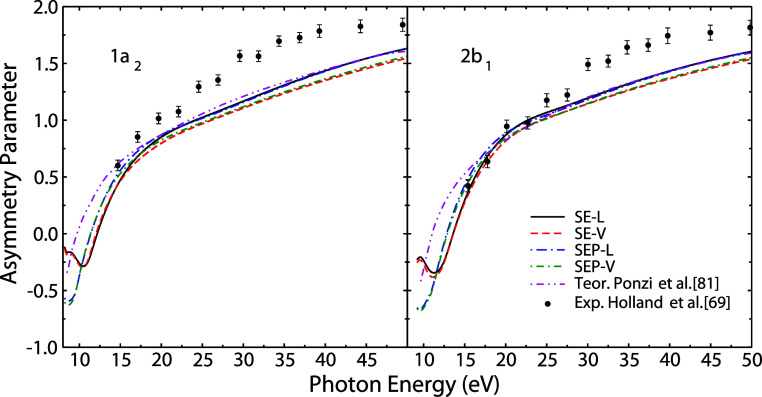
Asymmetry parameters of pyrrole’s two outermost valence orbitals. This work: solid black line, SE-*L*; short-dashed red line, SE-*V*; long-dash-dotted blue line. SEP-*L*; short-dash-dot green line, SEP-*V*. Theoretical: short-dash-dot-dot magenta line, data from ref 81; experimental: full black circle, data from ref 69.

## 4 Conclusions

A joint experimental and theoretical study on the photoionization of pyrrole molecules was presented. The experimental measurement is collected by the use of the double ionization chamber technique. The measured photoabsorption cross-section is reported from 8.1 to 21.5 eV. The quantum efficiency, photoionization cross-section, and neutral decay cross-sections are presented in the energy range 13.5–21.5 eV. The latter are presented for the first time. The comparison of our results of σ_a_ with others available in the literature shows good accordance in magnitude and structures observed. We also used our measured values of oscillator strength and combined with measured and extrapolated results to give a static polarizability of the pyrrole’s electric dipole of 50.3 au, which presents an error of less than 10% with the data available in the literature.

The calculated photoionization cross-section and asymmetry parameters are obtained using the ePolyScat.E computational package, which uses the Schwinger Variational Principle combined with Padé approximants. Our results of partial photoionization cross-sections and asymmetry parameters are shown from 8.2 to 50.0 eV energy range and are presented in four approaches: *L*/*V* and SE/SEP. The partial photoionization cross-sections for some orbitals, such as, 9a_1_, 6b_2_, and 5b_2_, present a resonance peak at the same energy where the asymmetry parameters exhibit a minimum.

We also compare our calculated photoionization cross-section summed for all 13 valence orbitals with our experimental σ_i_ and σ_a_. The behavior of σ_a_ near the ionization threshold indicates that polarization effects are necessary to give reliable results in this energy range. However, around 7 eV above the threshold, it is unnecessary to include polarization effects to compare with experimental photoionization cross-sections, near 17 eV. It suggests that the correlation-polarization is important near the threshold where the photoelectron is moving away slower. On the other hand, when the electron is relatively faster, the Coulomb interaction overcomes the polarization effects. The behavior near 17 eV shows that the summed photoionization cross-sections calculated at the *V* form gives better qualitative and in some cases quantitative agreement with measured data for pyrrole. In principle, the *L* and *V* forms should be equivalent, but in practice, the use of approximate wave functions leads to slightly different values.

Our asymmetry parameters, except for the two outermost orbitals, are presented for the first time. The results for the outermost orbitals 1a_2_ and 2b_1_ are compared with available results and show excellent agreement with another theoretical calculation and also good agreement with experimental values near the threshold and become lower at higher energies. In general, our theoretical and measured data are consistent with each other. These asymmetry parameter results could help experimentalists assign orbitals in the PES spectra.

Furthermore, photoionization cross-sections can be useful for proposing the reaction kinetics of the photodissociation pathways for the pyrrole molecule. Knowledge of σ_i_, together with information about the abundance of each ionic fragment produced, allows the estimation of the absolute value of the partial photoionization cross-section for each specific ionic fragment, which is an important quantity for understanding the photostability of molecules in space.

## Supplementary Material



## Notes

Auxiliary tables containing numerical values of the experimental and calculated data for some selected photon energies (PDF)
