# Supplementary material for: Photoabsorption and Photoionization Cross-Sections and Asymmetry Parameters of Pyrrole in the Vacuum-Ultraviolet Energy Range
Source: ACS Phys Chem Au. 2025 Feb 24;5(3):254–65. doi: 10.1021/acsphyschemau.4c00101 (PMC12123551; doi:10.1021/acsphyschemau.4c00101)
Supplement: Supplementary file 1 [file pg4c00101_si_001.pdf]

Supporting Information
Photoabsorption and Photoionization Cross Sections and Asymmetry Parameters of Pyrrole in the Vacuum-Ultraviolet Energy Range

Mariana B. M. S. Medeiros,† Josenilton N. Sousa,‡ Manuela S. Arruda,† Milton M. Fujimoto,¶ Manoel G. P. Homem,§ Helder K. Tanaka,‖ Bruno Credidio,† Ricardo R. T. Marinho,⊥ and Frederico V. Prudente*,†

†*Instituto de Física, Universidade Federal da Bahia, Salvador, BA, Brasil*

‡*Instituto Federal Baiano, Campus - Guanambi, BA, Brasil*

¶*Departamento de Física, Universidade Federal do Paraná, Curitiba, PR, Brasil*

§*Departamento de Química, Universidade Federal de São Carlos, São Carlos, SP, Brasil*

‖*Instituto Federal da Bahia, Campus - Porto Seguro, BA, Brasil*

⊥*Instituto de Física, Universidade de Brasilia, Brasília, DF, Brasil.*

E-mail: prudente@ufba.br

1 Tables

Table S1: Experimental results for photoabsorption cross sections (σ_a), ionization efficiency (η), photoionization cross sections (σ_i), and neutral-decay cross sections (σ_n) of Pyrrole. Energy in eV and cross section in MB.

E	σ_a	η	σ_i	σ_n	E	σ_a	η	σ_i	σ_n	E	σ_a	η	σ_i	σ_n
8.2	22.719				12.7	70.726				17.2	118.524	0.913	108.225	10.298
8.3	22.700				12.8	71.245				17.3	117.720	0.915	107.710	10.011
8.4	16.619				12.9	71.160				17.4	116.951	0.919	107.480	9.471
8.5	16.276				13.0	71.117				17.5	115.858	0.922	106.832	9.025
8.6	15.594				13.1	70.892				17.6	115.007	0.922	106.062	8.946
8.7	13.641				13.2	70.389				17.7	113.943	0.926	105.476	8.467
8.8	13.317				13.3	70.746				17.8	113.040	0.929	105.023	8.017
8.9	14.487				13.4	70.367				17.9	112.144	0.931	104.412	7.732
9.0	15.203				13.5	71.159	0.718	51.109	20.050	18.0	111.630	0.935	104.400	7.230
9.1	17.021				13.6	72.114	0.732	52.777	19.337	18.1	111.176	0.939	104.354	6.822
9.2	20.008				13.7	74.021	0.747	55.270	18.751	18.2	110.713	0.941	104.190	6.522
9.3	22.673				13.8	76.313	0.759	57.912	18.401	18.3	110.624	0.945	104.491	6.133
9.4	24.991				13.9	79.513	0.770	61.215	18.298	18.4	110.565	0.946	104.547	6.018
9.5	26.844				14.0	82.255	0.779	64.084	18.171	18.5	110.473	0.949	104.819	5.654
9.6	28.217				14.1	83.947	0.791	66.391	17.556	18.6	110.381	0.952	105.038	5.343
9.7	29.273				14.2	85.350	0.809	69.033	16.318	18.7	110.415	0.952	105.134	5.280
9.8	29.921				14.3	87.064	0.829	72.141	14.923	18.8	110.341	0.954	105.279	5.062
9.9	30.452				14.4	88.597	0.844	74.816	13.782	18.9	110.219	0.957	105.496	4.723
10.0	30.962				14.5	89.967	0.859	77.316	12.651	19.0	110.103	0.960	105.685	4.418
10.1	33.294				14.6	91.989	0.873	80.337	11.652	19.1	109.825	0.962	105.673	4.152
10.2	34.358				14.7	94.466	0.881	83.181	11.285	19.2	109.755	0.966	106.034	3.720
10.3	36.816				14.8	96.908	0.885	85.747	11.161	19.3	109.336	0.970	106.087	3.249
10.4	39.170				14.9	98.789	0.889	87.815	10.975	19.4	109.155	0.974	106.349	2.806
10.5	41.959				15.0	100.644	0.894	89.950	10.694	19.5	108.699	0.975	106.022	2.677
10.6	45.296				15.1	102.329	0.898	91.926	10.403	19.6	108.494	0.978	106.124	2.371
10.7	48.765				15.2	103.861	0.902	93.676	10.185	19.7	108.113	0.981	106.065	2.048
10.8	51.877				15.3	105.928	0.905	95.826	10.102	19.8	107.983	0.986	106.456	1.527
10.9	54.178				15.4	107.965	0.910	98.199	9.766	19.9	107.470	0.986	106.018	1.452
11.0	56.741				15.5	109.923	0.912	100.209	9.714	20.0	107.325	0.984	105.620	1.706
11.1	57.782				15.6	111.732	0.911	101.788	9.945	20.1	107.061	0.985	105.469	1.593
11.2	57.689				15.7	112.937	0.910	102.752	10.185	20.2	106.595	0.988	105.360	1.236
11.3	58.614				15.8	114.466	0.910	104.155	10.311	20.3	106.253	0.990	105.151	1.102
11.4	60.190				15.9	115.737	0.909	105.155	10.582	20.4	106.279	0.991	105.341	0.937
11.5	62.899				16.0	116.970	0.907	106.040	10.930	20.5	105.912	0.991	104.990	0.922
11.6	64.982				16.1	117.798	0.904	106.516	11.282	20.6	105.468	0.993	104.722	0.746
11.7	67.652				16.2	118.944	0.903	107.413	11.531	20.7	105.207	0.992	104.393	0.814
11.8	66.721				16.3	119.885	0.905	108.445	11.440	20.8	104.702	0.992	103.842	0.860
11.9	66.747				16.4	120.734	0.904	109.134	11.600	20.9	104.416	0.995	103.847	0.570
12.0	67.177				16.5	121.077	0.905	109.535	11.542	21.0	103.991	0.997	103.670	0.321
12.1	68.139				16.6	120.943	0.908	109.771	11.172	21.1	103.729	0.999	103.605	0.123
12.2	69.331				16.7	120.961	0.910	110.109	10.851	21.2	103.318	0.998	103.128	0.190
12.3	70.124				16.8	120.680	0.901	108.729	11.951	21.3	102.884	0.997	102.568	0.316
12.4	70.854				16.9	120.289	0.899	108.195	12.093	21.4	102.570	0.997	102.280	0.290
12.5	71.026				17.0	119.727	0.910	108.993	10.733	21.5	102.078	0.996	101.700	0.378
12.6	70.959				17.1	119.193	0.911	108.572	10.621					

Table S2: Theoretical partial photoionization cross section for $1a_2$ Orbital. Energy in eV and cross section in MB.

E	SEP-L	SE-L	SEP-V	SE-V	E	SEP-L	SE-L	SEP-V	SE-V	E	SEP-L	SE-L	SEP-V	SE-V
8.25	53.252	27.889	27.416	20.508	22.25	5.850	4.651	3.972	3.691	36.25	1.891	1.605	1.402	1.323
8.50	54.075	22.839	28.648	17.131	22.50	5.682	4.537	3.870	3.606	36.50	1.861	1.580	1.379	1.301
8.75	56.869	20.154	30.526	15.416	22.75	5.524	4.429	3.772	3.526	36.75	1.830	1.555	1.357	1.280
9.00	55.577	18.615	29.997	14.500	23.00	5.373	4.325	3.679	3.449	37.00	1.801	1.531	1.335	1.259
9.25	49.317	17.676	26.782	14.011	23.25	5.229	4.226	3.591	3.376	37.25	1.772	1.507	1.314	1.239
9.50	41.492	17.164	22.781	13.857	23.50	5.093	4.132	3.507	3.306	37.50	1.744	1.484	1.293	1.219
9.75	34.702	17.119	19.371	14.074	23.75	4.963	4.041	3.427	3.239	37.75	1.716	1.462	1.272	1.200
10.00	29.595	17.374	16.868	14.466	24.00	4.840	3.954	3.351	3.174	38.00	1.689	1.440	1.252	1.181
10.25	25.944	17.616	15.136	14.764	24.25	4.722	3.870	3.279	3.112	38.25	1.663	1.418	1.232	1.163
10.50	23.379	17.743	13.976	14.916	24.50	4.609	3.789	3.209	3.052	38.50	1.638	1.397	1.213	1.145
10.75	21.609	17.775	13.234	14.961	24.75	4.502	3.711	3.143	2.993	38.75	1.612	1.377	1.194	1.127
11.00	20.437	17.734	12.810	14.926	25.00	4.399	3.636	3.079	2.937	39.00	1.588	1.357	1.176	1.110
11.25	19.708	17.633	12.610	14.825	25.25	4.300	3.563	3.018	2.882	39.25	1.564	1.337	1.158	1.093
11.50	19.269	17.479	12.537	14.668	25.50	4.205	3.492	2.959	2.829	39.50	1.540	1.318	1.140	1.076
11.75	18.985	17.276	12.508	14.461	25.75	4.115	3.424	2.902	2.778	39.75	1.518	1.299	1.122	1.060
12.00	18.766	17.028	12.475	14.209	26.00	4.027	3.357	2.847	2.727	40.00	1.495	1.281	1.106	1.044
12.25	18.566	16.740	12.416	13.919	26.25	3.943	3.292	2.794	2.678	40.25	1.473	1.263	1.089	1.029
12.50	18.363	16.415	12.328	13.595	26.50	3.861	3.229	2.743	2.630	40.50	1.452	1.245	1.073	1.014
12.75	18.149	16.059	12.212	13.245	26.75	3.783	3.167	2.693	2.583	40.75	1.431	1.228	1.057	0.999
13.00	17.918	15.677	12.068	12.874	27.00	3.707	3.107	2.644	2.536	41.00	1.410	1.212	1.041	0.984
13.25	17.666	15.273	11.900	12.487	27.25	3.633	3.048	2.597	2.491	41.25	1.390	1.195	1.026	0.970
13.50	17.392	14.853	11.708	12.089	27.50	3.561	2.991	2.551	2.447	41.50	1.371	1.179	1.011	0.956
13.75	17.095	14.420	11.494	11.685	27.75	3.492	2.934	2.507	2.403	41.75	1.352	1.164	0.996	0.943
14.00	16.776	13.979	11.261	11.279	28.00	3.424	2.879	2.463	2.361	42.00	1.333	1.148	0.982	0.930
14.25	16.434	13.534	11.012	10.875	28.25	3.359	2.826	2.420	2.319	42.25	1.314	1.133	0.968	0.917
14.50	16.073	13.088	10.747	10.475	28.50	3.295	2.773	2.378	2.277	42.50	1.296	1.118	0.955	0.904
14.75	15.694	12.644	10.471	10.082	28.75	3.232	2.721	2.337	2.237	42.75	1.279	1.104	0.941	0.892
15.00	15.299	12.205	10.186	9.698	29.00	3.171	2.671	2.297	2.197	43.00	1.262	1.090	0.928	0.879
15.25	14.892	11.773	9.895	9.325	29.25	3.112	2.622	2.258	2.158	43.25	1.245	1.076	0.915	0.867
15.50	14.475	11.351	9.599	8.964	29.50	3.054	2.573	2.219	2.120	43.50	1.228	1.062	0.903	0.856
15.75	14.052	10.939	9.301	8.616	29.75	2.997	2.526	2.181	2.082	43.75	1.212	1.049	0.890	0.844
16.00	13.625	10.539	9.003	8.282	30.00	2.942	2.480	2.144	2.045	44.00	1.196	1.036	0.878	0.833
16.25	13.197	10.153	8.707	7.962	30.25	2.888	2.434	2.107	2.009	44.25	1.181	1.023	0.866	0.822
16.50	12.771	9.781	8.416	7.657	30.50	2.835	2.390	2.071	1.973	44.50	1.165	1.011	0.855	0.812
16.75	12.348	9.423	8.129	7.366	30.75	2.783	2.347	2.036	1.938	44.75	1.150	0.999	0.843	0.801
17.00	11.931	9.081	7.848	7.090	31.00	2.732	2.304	2.001	1.904	45.00	1.136	0.987	0.832	0.791
17.25	11.522	8.752	7.576	6.827	31.25	2.683	2.263	1.967	1.870	45.25	1.121	0.975	0.821	0.781
17.50	11.122	8.439	7.311	6.578	31.50	2.634	2.222	1.933	1.837	45.50	1.107	0.963	0.811	0.771
17.75	10.733	8.138	7.055	6.342	31.75	2.587	2.182	1.900	1.805	45.75	1.094	0.952	0.800	0.761
18.00	10.355	7.851	6.809	6.117	32.00	2.541	2.144	1.868	1.773	46.00	1.080	0.941	0.790	0.752
18.25	9.990	7.577	6.572	5.903	32.25	2.495	2.106	1.836	1.742	46.25	1.067	0.930	0.780	0.743
18.50	9.637	7.316	6.345	5.700	32.50	2.451	2.069	1.805	1.711	46.50	1.054	0.919	0.770	0.734
18.75	9.298	7.066	6.128	5.508	32.75	2.407	2.032	1.775	1.681	46.75	1.041	0.909	0.761	0.725
19.00	8.973	6.829	5.920	5.326	33.00	2.365	1.997	1.744	1.652	47.00	1.029	0.899	0.751	0.716
19.25	8.661	6.603	5.723	5.154	33.25	2.323	1.962	1.715	1.623	47.25	1.016	0.888	0.742	0.708
19.50	8.362	6.389	5.534	4.991	33.50	2.283	1.929	1.686	1.595	47.50	1.004	0.879	0.733	0.699
19.75	8.076	6.185	5.354	4.837	33.75	2.243	1.896	1.658	1.568	47.75	0.992	0.869	0.724	0.691
20.00	7.802	5.993	5.182	4.691	34.00	2.204	1.863	1.630	1.541	48.00	0.981	0.859	0.715	0.683
20.25	7.541	5.810	5.019	4.554	34.25	2.166	1.832	1.602	1.514	48.25	0.969	0.850	0.707	0.675
20.50	7.291	5.636	4.864	4.424	34.50	2.129	1.801	1.575	1.488	48.50	0.958	0.841	0.698	0.668
20.75	7.053	5.472	4.716	4.301	34.75	2.093	1.771	1.549	1.463	48.75	0.947	0.832	0.690	0.660
21.00	6.827	5.317	4.575	4.185	35.00	2.057	1.742	1.523	1.439	49.00	0.936	0.823	0.682	0.653
21.25	6.611	5.169	4.442	4.075	35.25	2.022	1.713	1.498	1.414	49.25	0.926	0.814	0.674	0.645
21.50	6.406	5.030	4.315	3.971	35.50	1.989	1.685	1.473	1.391	49.50	0.916	0.805	0.667	0.638
21.75	6.211	4.897	4.195	3.873	35.75	1.955	1.658	1.449	1.368	49.75	0.905	0.797	0.659	0.631
22.00	6.026	4.771	4.081	3.779	36.00	1.923	1.631	1.425	1.345	50.00	0.895	0.789	0.652	0.625

Table S3: Theoretical partial photoionization cross section for $2b_1$ Orbital. Energy in eV and cross section in MB.

E	SEP-L	SE-L	SEP-V	SE-V	E	SEP-L	SE-L	SEP-V	SE-V	E	SEP-L	SE-L	SEP-V	SE-V
9.25	55.227	11.961	34.034	10.233	23.00	6.402	5.213	4.300	4.025	36.75	2.333	2.008	1.652	1.575
9.50	45.410	11.679	28.396	9.991	23.25	6.242	5.094	4.197	3.936	37.00	2.298	1.978	1.627	1.552
9.75	34.992	11.611	21.996	9.932	23.50	6.088	4.981	4.098	3.850	37.25	2.263	1.949	1.603	1.529
10.00	27.406	11.626	17.329	9.943	23.75	5.940	4.873	4.002	3.768	37.50	2.229	1.920	1.580	1.506
10.25	22.498	11.641	14.343	9.954	24.00	5.798	4.770	3.911	3.690	37.75	2.195	1.892	1.557	1.484
10.50	19.356	11.610	12.464	9.939	24.25	5.662	4.670	3.824	3.615	38.00	2.163	1.865	1.534	1.462
10.75	17.298	11.583	11.262	9.944	24.50	5.531	4.575	3.741	3.544	38.25	2.131	1.838	1.512	1.440
11.00	15.911	11.685	10.471	10.080	24.75	5.406	4.483	3.660	3.475	38.50	2.099	1.811	1.490	1.420
11.25	14.944	11.948	9.935	10.330	25.00	5.286	4.395	3.584	3.408	38.75	2.069	1.785	1.468	1.399
11.50	14.251	12.285	9.564	10.618	25.25	5.170	4.310	3.510	3.344	39.00	2.038	1.760	1.447	1.379
11.75	13.748	12.596	9.308	10.865	25.50	5.060	4.228	3.440	3.283	39.25	2.009	1.735	1.426	1.359
12.00	13.398	12.842	9.146	11.043	25.75	4.954	4.149	3.372	3.223	39.50	1.980	1.711	1.406	1.340
12.25	13.184	13.018	9.070	11.151	26.00	4.851	4.072	3.307	3.165	39.75	1.951	1.687	1.386	1.321
12.50	13.092	13.127	9.067	11.198	26.25	4.753	3.998	3.244	3.109	40.00	1.923	1.664	1.367	1.302
12.75	13.092	13.176	9.116	11.190	26.50	4.659	3.926	3.184	3.055	40.25	1.896	1.641	1.348	1.284
13.00	13.150	13.174	9.190	11.136	26.75	4.567	3.856	3.126	3.002	40.50	1.869	1.619	1.329	1.266
13.25	13.229	13.125	9.265	11.043	27.00	4.479	3.788	3.069	2.951	40.75	1.843	1.597	1.310	1.249
13.50	13.306	13.037	9.326	10.916	27.25	4.394	3.722	3.015	2.901	41.00	1.817	1.575	1.292	1.232
13.75	13.365	12.914	9.365	10.761	27.50	4.312	3.657	2.962	2.852	41.25	1.792	1.554	1.274	1.215
14.00	13.397	12.762	9.378	10.582	27.75	4.233	3.594	2.911	2.804	41.50	1.767	1.533	1.257	1.198
14.25	13.401	12.585	9.366	10.385	28.00	4.156	3.533	2.862	2.758	41.75	1.743	1.513	1.240	1.182
14.50	13.374	12.387	9.329	10.174	28.25	4.081	3.473	2.814	2.712	42.00	1.719	1.493	1.223	1.167
14.75	13.318	12.172	9.268	9.951	28.50	4.008	3.414	2.767	2.667	42.25	1.696	1.474	1.207	1.151
15.00	13.233	11.944	9.187	9.719	28.75	3.937	3.356	2.721	2.623	42.50	1.673	1.454	1.191	1.136
15.25	13.122	11.705	9.086	9.483	29.00	3.868	3.300	2.676	2.580	42.75	1.651	1.436	1.175	1.121
15.50	12.987	11.457	8.968	9.243	29.25	3.801	3.245	2.633	2.538	43.00	1.629	1.417	1.159	1.107
15.75	12.830	11.204	8.836	9.001	29.50	3.736	3.191	2.590	2.497	43.25	1.607	1.399	1.144	1.092
16.00	12.654	10.946	8.691	8.760	29.75	3.672	3.138	2.549	2.456	43.50	1.586	1.382	1.129	1.078
16.25	12.460	10.686	8.536	8.520	30.00	3.609	3.086	2.508	2.416	43.75	1.565	1.364	1.115	1.065
16.50	12.252	10.425	8.372	8.282	30.25	3.548	3.035	2.468	2.377	44.00	1.545	1.347	1.100	1.051
16.75	12.032	10.165	8.201	8.048	30.50	3.489	2.985	2.429	2.339	44.25	1.525	1.331	1.086	1.038
17.00	11.802	9.905	8.025	7.818	30.75	3.430	2.936	2.391	2.301	44.50	1.505	1.314	1.073	1.025
17.25	11.563	9.647	7.845	7.592	31.00	3.373	2.888	2.353	2.264	44.75	1.486	1.298	1.059	1.012
17.50	11.319	9.392	7.663	7.372	31.25	3.317	2.841	2.316	2.227	45.00	1.467	1.283	1.046	1.000
17.75	11.069	9.141	7.480	7.157	31.50	3.263	2.794	2.280	2.192	45.25	1.449	1.267	1.033	0.988
18.00	10.817	8.893	7.296	6.947	31.75	3.209	2.749	2.245	2.157	45.50	1.431	1.252	1.020	0.976
18.25	10.563	8.650	7.113	6.744	32.00	3.156	2.705	2.210	2.122	45.75	1.413	1.237	1.008	0.964
18.50	10.308	8.412	6.931	6.546	32.25	3.105	2.661	2.175	2.088	46.00	1.395	1.222	0.995	0.953
18.75	10.055	8.180	6.752	6.354	32.50	3.054	2.618	2.142	2.055	46.25	1.378	1.208	0.983	0.942
19.00	9.802	7.953	6.575	6.169	32.75	3.005	2.576	2.109	2.022	46.50	1.361	1.194	0.972	0.931
19.25	9.552	7.732	6.401	5.990	33.00	2.957	2.535	2.076	1.990	46.75	1.345	1.180	0.960	0.920
19.50	9.305	7.517	6.230	5.818	33.25	2.909	2.495	2.044	1.959	47.00	1.329	1.167	0.949	0.909
19.75	9.062	7.309	6.064	5.651	33.50	2.862	2.455	2.013	1.928	47.25	1.313	1.153	0.938	0.899
20.00	8.823	7.108	5.901	5.491	33.75	2.817	2.417	1.982	1.898	47.50	1.297	1.140	0.927	0.889
20.25	8.589	6.913	5.742	5.338	34.00	2.772	2.379	1.952	1.868	47.75	1.282	1.128	0.916	0.879
20.50	8.360	6.725	5.588	5.190	34.25	2.728	2.341	1.922	1.839	48.00	1.267	1.115	0.905	0.869
20.75	8.136	6.544	5.438	5.048	34.50	2.685	2.305	1.893	1.810	48.25	1.252	1.103	0.895	0.859
21.00	7.918	6.370	5.293	4.913	34.75	2.643	2.269	1.864	1.782	48.50	1.238	1.091	0.885	0.850
21.25	7.706	6.203	5.153	4.783	35.00	2.601	2.234	1.836	1.754	48.75	1.223	1.079	0.875	0.840
21.50	7.501	6.042	5.017	4.659	35.25	2.560	2.200	1.808	1.727	49.00	1.209	1.067	0.865	0.831
21.75	7.301	5.888	4.886	4.541	35.50	2.521	2.166	1.781	1.701	49.25	1.196	1.056	0.856	0.822
22.00	7.108	5.741	4.759	4.428	35.75	2.482	2.133	1.754	1.675	49.50	1.182	1.044	0.846	0.814
22.25	6.922	5.600	4.638	4.320	36.00	2.443	2.101	1.728	1.649	49.75	1.169	1.033	0.837	0.805
22.50	6.742	5.465	4.521	4.217	36.25	2.406	2.069	1.702	1.624	50.00	1.156	1.022	0.828	0.797
22.75	6.569	5.336	4.408	4.119	36.50	2.369	2.038	1.677	1.600					

Table S4: Theoretical partial photoionization cross section for $9a_1$ Orbital. Energy in eV and cross section in MB.

E	SEP-L	SE-L	SEP-V	SE-V	E	SEP-L	SE-L	SEP-V	SE-V	E	SEP-L	SE-L	SEP-V	SE-V
13.00	8.799	12.809	7.453	12.547	25.50	10.183	7.558	7.365	6.262	38.00	2.277	1.881	1.705	1.558
13.25	9.619	15.158	8.100	14.759	25.75	9.889	7.274	7.151	6.028	38.25	2.229	1.844	1.670	1.527
13.50	10.660	18.441	8.925	17.855	26.00	9.581	7.004	6.929	5.805	38.50	2.183	1.808	1.636	1.497
13.75	11.974	22.865	9.970	22.056	26.25	9.265	6.748	6.701	5.593	38.75	2.138	1.773	1.603	1.467
14.00	13.609	28.659	11.273	27.486	26.50	8.946	6.505	6.473	5.393	39.00	2.094	1.738	1.570	1.438
14.25	15.621	35.517	12.874	33.917	26.75	8.628	6.275	6.247	5.202	39.25	2.051	1.705	1.539	1.410
14.50	18.059	42.521	14.814	40.445	27.00	8.317	6.056	6.025	5.021	39.50	2.010	1.672	1.508	1.382
14.75	20.957	47.939	17.112	45.425	27.25	8.014	5.848	5.810	4.849	39.75	1.970	1.640	1.478	1.355
15.00	24.309	50.071	19.760	47.265	27.50	7.722	5.650	5.603	4.685	40.00	1.931	1.609	1.449	1.329
15.25	28.033	48.533	22.686	45.639	27.75	7.442	5.461	5.404	4.529	40.25	1.892	1.579	1.421	1.303
15.50	31.938	44.447	25.735	41.633	28.00	7.173	5.281	5.214	4.380	40.50	1.855	1.549	1.393	1.278
15.75	35.707	39.369	28.647	36.730	28.25	6.916	5.109	5.032	4.237	40.75	1.819	1.520	1.366	1.253
16.00	38.914	34.397	31.087	31.957	28.50	6.672	4.945	4.859	4.101	41.00	1.783	1.492	1.340	1.229
16.25	41.130	30.034	32.715	27.781	28.75	6.439	4.787	4.694	3.970	41.25	1.749	1.465	1.314	1.206
16.50	42.054	26.399	33.308	24.309	29.00	6.216	4.636	4.536	3.845	41.50	1.715	1.438	1.288	1.183
16.75	41.633	23.451	32.837	21.494	29.25	6.004	4.492	4.385	3.725	41.75	1.682	1.412	1.264	1.160
17.00	40.068	21.082	31.471	19.229	29.50	5.802	4.354	4.242	3.610	42.00	1.650	1.386	1.240	1.139
17.25	37.712	19.181	29.499	17.412	29.75	5.608	4.221	4.104	3.499	42.25	1.619	1.362	1.216	1.117
17.50	34.943	17.654	27.223	15.950	30.00	5.424	4.094	3.972	3.394	42.50	1.589	1.338	1.193	1.097
17.75	32.073	16.425	24.889	14.769	30.25	5.247	3.972	3.846	3.292	42.75	1.559	1.314	1.170	1.077
18.00	29.313	15.414	22.659	13.783	30.50	5.078	3.855	3.726	3.195	43.00	1.530	1.291	1.148	1.057
18.25	26.779	14.618	20.622	13.027	30.75	4.916	3.743	3.610	3.102	43.25	1.502	1.269	1.127	1.038
18.50	24.522	13.981	18.815	12.402	31.00	4.761	3.636	3.499	3.013	43.50	1.474	1.247	1.106	1.019
18.75	22.551	13.420	17.241	11.847	31.25	4.612	3.533	3.393	2.928	43.75	1.448	1.226	1.086	1.001
19.00	20.852	12.977	15.887	11.404	31.50	4.470	3.435	3.291	2.846	44.00	1.422	1.206	1.066	0.984
19.25	19.398	12.604	14.731	11.034	31.75	4.334	3.341	3.193	2.768	44.25	1.396	1.186	1.047	0.967
19.50	18.159	12.300	13.747	10.720	32.00	4.203	3.251	3.099	2.694	44.50	1.372	1.167	1.028	0.950
19.75	17.107	12.046	12.912	10.458	32.25	4.078	3.164	3.009	2.622	44.75	1.348	1.148	1.009	0.934
20.00	16.215	11.842	12.204	10.239	32.50	3.958	3.082	2.923	2.554	45.00	1.324	1.130	0.992	0.919
20.25	15.457	11.672	11.603	10.053	32.75	3.843	3.002	2.840	2.488	45.25	1.302	1.112	0.974	0.903
20.50	14.815	11.536	11.092	9.897	33.00	3.734	2.927	2.761	2.425	45.50	1.280	1.095	0.957	0.889
20.75	14.269	11.429	10.659	9.768	33.25	3.628	2.854	2.685	2.365	45.75	1.258	1.078	0.941	0.874
21.00	13.806	11.346	10.288	9.661	33.50	3.528	2.784	2.612	2.307	46.00	1.237	1.062	0.925	0.861
21.25	13.411	11.282	9.971	9.571	33.75	3.431	2.717	2.542	2.252	46.25	1.217	1.046	0.909	0.847
21.50	13.077	11.229	9.701	9.493	34.00	3.339	2.653	2.476	2.199	46.50	1.197	1.031	0.894	0.834
21.75	12.792	11.179	9.470	9.418	34.25	3.251	2.591	2.412	2.147	46.75	1.178	1.016	0.880	0.821
22.00	12.550	11.121	9.272	9.339	34.50	3.166	2.531	2.350	2.098	47.00	1.160	1.001	0.865	0.809
22.25	12.344	11.043	9.101	9.247	34.75	3.085	2.474	2.292	2.051	47.25	1.142	0.987	0.851	0.797
22.50	12.169	10.935	8.954	9.133	35.00	3.007	2.419	2.235	2.005	47.50	1.124	0.973	0.838	0.785
22.75	12.019	10.790	8.826	8.991	35.25	2.932	2.366	2.181	1.961	47.75	1.107	0.960	0.825	0.774
23.00	11.888	10.602	8.713	8.818	35.50	2.861	2.314	2.129	1.919	48.00	1.090	0.947	0.812	0.762
23.25	11.770	10.371	8.610	8.614	35.75	2.792	2.265	2.080	1.878	48.25	1.074	0.934	0.800	0.752
23.50	11.657	10.103	8.512	8.382	36.00	2.726	2.217	2.032	1.838	48.50	1.058	0.922	0.788	0.741
23.75	11.543	9.805	8.413	8.129	36.25	2.663	2.170	1.986	1.799	48.75	1.043	0.910	0.776	0.731
24.00	11.419	9.486	8.309	7.860	36.50	2.601	2.125	1.941	1.762	49.00	1.028	0.898	0.765	0.721
24.25	11.278	9.155	8.194	7.584	36.75	2.543	2.081	1.898	1.725	49.25	1.014	0.887	0.754	0.712
24.50	11.115	8.821	8.064	7.306	37.00	2.486	2.039	1.857	1.690	49.50	1.000	0.876	0.743	0.702
24.75	10.924	8.490	7.917	7.032	37.25	2.431	1.998	1.817	1.656	49.75	0.986	0.865	0.732	0.693
25.00	10.704	8.168	7.751	6.765	37.50	2.378	1.958	1.778	1.622	50.00	0.973	0.855	0.722	0.684
25.25	10.456	7.856	7.566	6.508	37.75	2.327	1.919	1.741	1.590					

Table S5: Theoretical partial photoionization cross section for $6b_2$ Orbital. Energy in eV and cross section in MB.

E	SEP-L	SE-L	SEP-V	SE-V	E	SEP-L	SE-L	SEP-V	SE-V	E	SEP-L	SE-L	SEP-V	SE-V
13.00	9.200	11.239	8.089	11.261	25.50	10.970	8.641	8.001	7.123	38.00	2.610	2.113	1.939	1.748
13.25	9.663	12.435	8.420	12.424	25.75	10.726	8.366	7.811	6.887	38.25	2.548	2.069	1.896	1.712
13.50	10.239	14.200	8.900	14.164	26.00	10.465	8.101	7.609	6.659	38.50	2.489	2.026	1.854	1.677
13.75	10.968	16.765	9.486	16.705	26.25	10.192	7.847	7.400	6.442	38.75	2.432	1.984	1.814	1.642
14.00	11.877	20.394	10.233	20.313	26.50	9.911	7.603	7.186	6.234	39.00	2.377	1.944	1.775	1.609
14.25	13.006	25.217	11.169	25.096	26.75	9.629	7.369	6.973	6.036	39.25	2.325	1.904	1.737	1.576
14.50	14.405	30.771	12.330	30.576	27.00	9.348	7.145	6.761	5.846	39.50	2.274	1.866	1.701	1.544
14.75	16.122	35.484	13.759	35.157	27.25	9.070	6.929	6.554	5.664	39.75	2.225	1.829	1.665	1.513
15.00	18.193	37.472	15.483	37.003	27.50	8.798	6.722	6.352	5.490	40.00	2.177	1.793	1.631	1.483
15.25	20.622	36.438	17.499	35.818	27.75	8.534	6.523	6.156	5.323	40.25	2.131	1.758	1.597	1.454
15.50	23.342	33.599	19.746	32.841	28.00	8.277	6.331	5.966	5.162	40.50	2.086	1.724	1.565	1.425
15.75	26.164	30.233	22.056	29.356	28.25	8.029	6.145	5.784	5.007	40.75	2.043	1.691	1.533	1.397
16.00	28.750	27.063	24.141	26.085	28.50	7.790	5.966	5.609	4.858	41.00	2.001	1.659	1.503	1.369
16.25	30.674	24.364	25.646	23.299	28.75	7.558	5.792	5.440	4.714	41.25	1.961	1.628	1.473	1.342
16.50	31.599	22.172	26.295	21.033	29.00	7.335	5.623	5.277	4.575	41.50	1.921	1.597	1.443	1.316
16.75	31.458	20.430	26.046	19.225	29.25	7.119	5.460	5.121	4.440	41.75	1.883	1.567	1.415	1.290
17.00	30.456	19.054	25.082	17.791	29.50	6.911	5.300	4.971	4.310	42.00	1.846	1.538	1.387	1.265
17.25	28.925	17.966	23.689	16.649	29.75	6.709	5.145	4.826	4.183	42.25	1.810	1.510	1.360	1.241
17.50	27.171	17.099	22.124	15.733	30.00	6.514	4.995	4.686	4.060	42.50	1.774	1.483	1.333	1.217
17.75	25.408	16.397	20.568	14.985	30.25	6.325	4.848	4.550	3.940	42.75	1.740	1.456	1.307	1.194
18.00	23.760	15.818	19.119	14.367	30.50	6.142	4.705	4.419	3.824	43.00	1.707	1.430	1.282	1.171
18.25	22.282	15.329	17.824	13.838	30.75	5.963	4.566	4.292	3.711	43.25	1.675	1.405	1.257	1.149
18.50	20.990	14.906	16.693	13.381	31.00	5.790	4.430	4.169	3.602	43.50	1.643	1.380	1.233	1.127
18.75	19.874	14.530	15.716	12.975	31.25	5.621	4.299	4.049	3.496	43.75	1.613	1.356	1.209	1.106
19.00	18.917	14.185	14.877	12.602	31.50	5.457	4.171	3.933	3.393	44.00	1.583	1.332	1.186	1.086
19.25	18.095	13.864	14.157	12.259	31.75	5.297	4.048	3.820	3.294	44.25	1.554	1.309	1.163	1.065
19.50	17.387	13.560	13.536	11.936	32.00	5.141	3.928	3.710	3.198	44.50	1.525	1.287	1.141	1.046
19.75	16.773	13.263	12.995	11.621	32.25	4.989	3.812	3.604	3.105	44.75	1.498	1.265	1.120	1.027
20.00	16.234	12.987	12.521	11.333	32.50	4.841	3.701	3.500	3.015	45.00	1.471	1.244	1.099	1.008
20.25	15.756	12.728	12.100	11.062	32.75	4.698	3.593	3.399	2.929	45.25	1.445	1.223	1.079	0.990
20.50	15.327	12.489	11.722	10.815	33.00	4.558	3.489	3.301	2.847	45.50	1.419	1.203	1.059	0.972
20.75	14.938	12.275	11.379	10.591	33.25	4.422	3.390	3.206	2.767	45.75	1.395	1.183	1.039	0.955
21.00	14.580	12.087	11.065	10.394	33.50	4.291	3.294	3.114	2.691	46.00	1.370	1.164	1.020	0.938
21.25	14.249	11.931	10.776	10.226	33.75	4.164	3.203	3.025	2.618	46.25	1.347	1.145	1.002	0.922
21.50	13.940	11.806	10.508	10.088	34.00	4.041	3.115	2.939	2.548	46.50	1.324	1.127	0.984	0.906
21.75	13.653	11.711	10.260	9.978	34.25	3.922	3.030	2.856	2.481	46.75	1.301	1.109	0.966	0.890
22.00	13.384	11.638	10.029	9.887	34.50	3.807	2.950	2.776	2.417	47.00	1.279	1.091	0.949	0.875
22.25	13.136	11.575	9.817	9.807	34.75	3.697	2.873	2.699	2.356	47.25	1.258	1.074	0.932	0.860
22.50	12.908	11.505	9.622	9.722	35.00	3.590	2.799	2.625	2.297	47.50	1.237	1.058	0.916	0.846
22.75	12.701	11.414	9.446	9.619	35.25	3.488	2.728	2.554	2.241	47.75	1.217	1.041	0.900	0.832
23.00	12.516	11.288	9.289	9.489	35.50	3.390	2.660	2.485	2.187	48.00	1.197	1.025	0.885	0.818
23.25	12.353	11.121	9.150	9.327	35.75	3.296	2.595	2.420	2.135	48.25	1.178	1.010	0.869	0.805
23.50	12.209	10.914	9.027	9.132	36.00	3.206	2.533	2.357	2.085	48.50	1.159	0.994	0.855	0.792
23.75	12.080	10.671	8.916	8.909	36.25	3.120	2.473	2.297	2.038	48.75	1.140	0.980	0.840	0.779
24.00	11.959	10.402	8.812	8.666	36.50	3.038	2.416	2.239	1.992	49.00	1.122	0.965	0.826	0.766
24.25	11.838	10.115	8.708	8.410	36.75	2.958	2.361	2.184	1.948	49.25	1.105	0.951	0.813	0.754
24.50	11.707	9.818	8.597	8.147	37.00	2.883	2.308	2.131	1.905	49.50	1.088	0.937	0.799	0.742
24.75	11.559	9.517	8.474	7.884	37.25	2.810	2.256	2.080	1.864	49.75	1.071	0.923	0.786	0.731
25.00	11.388	9.219	8.335	7.623	37.50	2.741	2.207	2.031	1.824	50.00	1.054	0.910	0.774	0.720
25.25	11.191	8.926	8.177	7.369	37.75	2.674	2.160	1.984	1.785					

Table S6: Theoretical partial photoionization cross section for $1b_1$ Orbital. Energy in eV and cross section in MB.

E	SEP-L	SE-L	SEP-V	SE-V	E	SEP-L	SE-L	SEP-V	SE-V	E	SEP-L	SE-L	SEP-V	SE-V
13.75	11.476	8.280	9.501	7.941	26.00	3.844	3.331	2.820	2.723	38.25	2.018	1.801	1.466	1.448
14.00	10.729	7.865	8.856	7.489	26.25	3.783	3.279	2.771	2.676	38.50	1.998	1.784	1.453	1.435
14.25	10.148	7.471	8.343	7.060	26.50	3.724	3.227	2.724	2.629	38.75	1.978	1.767	1.440	1.421
14.50	9.656	7.125	7.902	6.683	26.75	3.666	3.177	2.677	2.584	39.00	1.958	1.750	1.427	1.408
14.75	9.216	6.870	7.504	6.404	27.00	3.610	3.129	2.632	2.540	39.25	1.938	1.733	1.414	1.395
15.00	8.811	6.721	7.136	6.240	27.25	3.554	3.081	2.588	2.497	39.50	1.919	1.716	1.401	1.382
15.25	8.433	6.626	6.792	6.133	27.50	3.500	3.034	2.545	2.456	39.75	1.900	1.699	1.388	1.369
15.50	8.081	6.524	6.473	6.021	27.75	3.446	2.989	2.503	2.416	40.00	1.882	1.683	1.376	1.356
15.75	7.763	6.399	6.185	5.884	28.00	3.394	2.945	2.463	2.377	40.25	1.863	1.666	1.364	1.343
16.00	7.486	6.257	5.937	5.731	28.25	3.343	2.902	2.423	2.339	40.50	1.845	1.650	1.352	1.331
16.25	7.257	6.110	5.733	5.573	28.50	3.293	2.859	2.384	2.302	40.75	1.827	1.634	1.339	1.318
16.50	7.071	5.964	5.568	5.419	28.75	3.244	2.818	2.347	2.267	41.00	1.809	1.618	1.327	1.305
16.75	6.913	5.827	5.429	5.275	29.00	3.197	2.778	2.310	2.232	41.25	1.791	1.601	1.315	1.292
17.00	6.768	5.702	5.302	5.145	29.25	3.150	2.739	2.274	2.199	41.50	1.774	1.585	1.303	1.279
17.25	6.626	5.591	5.178	5.031	29.50	3.105	2.701	2.240	2.166	41.75	1.756	1.569	1.291	1.266
17.50	6.485	5.495	5.055	4.933	29.75	3.060	2.664	2.206	2.134	42.00	1.739	1.553	1.279	1.253
17.75	6.345	5.413	4.935	4.849	30.00	3.016	2.627	2.173	2.104	42.25	1.721	1.537	1.267	1.240
18.00	6.211	5.344	4.821	4.777	30.25	2.974	2.592	2.141	2.074	42.50	1.704	1.522	1.255	1.227
18.25	6.085	5.284	4.715	4.714	30.50	2.932	2.557	2.110	2.045	42.75	1.687	1.506	1.243	1.214
18.50	5.969	5.229	4.619	4.656	30.75	2.892	2.523	2.080	2.017	43.00	1.670	1.490	1.231	1.201
18.75	5.865	5.177	4.532	4.599	31.00	2.852	2.490	2.051	1.989	43.25	1.653	1.475	1.219	1.189
19.00	5.772	5.125	4.456	4.541	31.25	2.813	2.458	2.022	1.963	43.50	1.636	1.459	1.207	1.176
19.25	5.689	5.070	4.388	4.481	31.50	2.775	2.426	1.994	1.937	43.75	1.619	1.444	1.195	1.163
19.50	5.614	5.013	4.327	4.417	31.75	2.738	2.396	1.967	1.912	44.00	1.602	1.429	1.183	1.151
19.75	5.546	4.952	4.270	4.350	32.00	2.702	2.366	1.941	1.887	44.25	1.586	1.414	1.171	1.138
20.00	5.481	4.888	4.216	4.280	32.25	2.667	2.336	1.915	1.863	44.50	1.569	1.399	1.159	1.126
20.25	5.418	4.821	4.162	4.208	32.50	2.632	2.308	1.890	1.840	44.75	1.553	1.384	1.147	1.114
20.50	5.355	4.753	4.108	4.135	32.75	2.599	2.280	1.866	1.818	45.00	1.536	1.369	1.135	1.101
20.75	5.291	4.683	4.052	4.060	33.00	2.566	2.253	1.843	1.796	45.25	1.520	1.355	1.123	1.089
21.00	5.225	4.612	3.995	3.985	33.25	2.533	2.226	1.820	1.775	45.50	1.504	1.340	1.111	1.077
21.25	5.157	4.541	3.935	3.910	33.50	2.502	2.200	1.797	1.754	45.75	1.488	1.326	1.100	1.065
21.50	5.088	4.469	3.874	3.836	33.75	2.471	2.174	1.775	1.734	46.00	1.472	1.312	1.088	1.054
21.75	5.017	4.398	3.812	3.762	34.00	2.441	2.150	1.754	1.715	46.25	1.456	1.298	1.076	1.042
22.00	4.945	4.327	3.749	3.689	34.25	2.411	2.125	1.733	1.696	46.50	1.441	1.284	1.065	1.030
22.25	4.872	4.257	3.686	3.618	34.50	2.382	2.102	1.713	1.677	46.75	1.425	1.270	1.054	1.019
22.50	4.799	4.188	3.622	3.548	34.75	2.354	2.078	1.694	1.659	47.00	1.410	1.256	1.042	1.008
22.75	4.726	4.120	3.559	3.479	35.00	2.327	2.056	1.675	1.642	47.25	1.395	1.243	1.031	0.996
23.00	4.652	4.052	3.496	3.412	35.25	2.300	2.034	1.656	1.625	47.50	1.380	1.230	1.020	0.985
23.25	4.580	3.986	3.434	3.346	35.50	2.273	2.012	1.638	1.608	47.75	1.365	1.216	1.009	0.975
23.50	4.508	3.920	3.373	3.282	35.75	2.247	1.991	1.620	1.592	48.00	1.350	1.203	0.998	0.964
23.75	4.437	3.856	3.312	3.220	36.00	2.222	1.970	1.603	1.576	48.25	1.335	1.191	0.987	0.953
24.00	4.367	3.793	3.253	3.158	36.25	2.198	1.950	1.586	1.561	48.50	1.321	1.178	0.976	0.943
24.25	4.297	3.731	3.195	3.099	36.50	2.173	1.930	1.570	1.546	48.75	1.307	1.165	0.966	0.932
24.50	4.229	3.670	3.138	3.041	36.75	2.150	1.911	1.554	1.531	49.00	1.293	1.153	0.955	0.922
24.75	4.162	3.611	3.082	2.984	37.00	2.127	1.892	1.539	1.517	49.25	1.279	1.141	0.945	0.912
25.00	4.096	3.552	3.027	2.929	37.25	2.104	1.873	1.524	1.502	49.50	1.265	1.128	0.935	0.902
25.25	4.031	3.495	2.973	2.876	37.50	2.082	1.855	1.509	1.488	49.75	1.251	1.117	0.925	0.892
25.50	3.967	3.439	2.921	2.823	37.75	2.060	1.837	1.494	1.475	50.00	1.238	1.105	0.915	0.882
25.75	3.905	3.384	2.870	2.773	38.00	2.039	1.819	1.480	1.461					

Table S7: Theoretical partial photoionization cross section for $5b_2$ Orbital. Energy in eV and cross section in MB.

E	SEP-L	SE-L	SEP-V	SE-V	E	SEP-L	SE-L	SEP-V	SE-V	E	SEP-L	SE-L	SEP-V	SE-V
14.50	12.823	15.995	10.465	15.115	26.50	9.696	6.744	6.828	5.405	38.50	2.615	2.249	1.937	1.841
14.75	13.315	17.522	10.844	16.527	26.75	9.357	6.421	6.584	5.150	38.75	2.575	2.212	1.909	1.812
15.00	13.974	19.655	11.350	18.515	27.00	8.997	6.123	6.328	4.915	39.00	2.535	2.176	1.881	1.782
15.25	14.816	22.510	12.001	21.169	27.25	8.624	5.851	6.065	4.700	39.25	2.495	2.140	1.852	1.753
15.50	15.871	26.184	12.819	24.591	27.50	8.247	5.603	5.800	4.504	39.50	2.455	2.104	1.825	1.724
15.75	17.169	30.471	13.832	28.548	27.75	7.873	5.376	5.541	4.326	39.75	2.416	2.069	1.797	1.694
16.00	18.739	34.802	15.060	32.467	28.00	7.509	5.170	5.289	4.163	40.00	2.378	2.033	1.769	1.665
16.25	20.606	38.242	16.523	35.459	28.25	7.162	4.982	5.050	4.015	40.25	2.339	1.998	1.742	1.636
16.50	22.764	39.796	18.211	36.637	28.50	6.833	4.810	4.824	3.879	40.50	2.301	1.963	1.714	1.608
16.75	25.163	38.958	20.081	35.610	28.75	6.526	4.652	4.614	3.754	40.75	2.263	1.929	1.687	1.579
17.00	27.690	36.148	22.037	32.843	29.00	6.241	4.508	4.419	3.640	41.00	2.225	1.895	1.660	1.551
17.25	30.152	32.449	23.918	29.338	29.25	5.977	4.375	4.240	3.535	41.25	2.187	1.861	1.632	1.522
17.50	32.289	28.807	25.515	25.931	29.50	5.734	4.252	4.074	3.438	41.50	2.150	1.828	1.605	1.494
17.75	33.825	25.676	26.610	23.019	29.75	5.511	4.139	3.922	3.348	41.75	2.113	1.795	1.578	1.467
18.00	34.537	23.151	27.040	20.672	30.00	5.306	4.034	3.783	3.264	42.00	2.077	1.762	1.551	1.439
18.25	34.339	21.167	26.752	18.824	30.25	5.118	3.936	3.655	3.187	42.25	2.040	1.730	1.525	1.412
18.50	33.312	19.614	25.828	17.374	30.50	4.945	3.844	3.537	3.114	42.50	2.004	1.698	1.498	1.385
18.75	31.682	18.385	24.453	16.221	30.75	4.786	3.759	3.429	3.046	42.75	1.969	1.667	1.472	1.359
19.00	29.729	17.393	22.850	15.286	31.00	4.639	3.678	3.330	2.982	43.00	1.934	1.637	1.445	1.333
19.25	27.702	16.566	21.209	14.505	31.25	4.503	3.603	3.237	2.922	43.25	1.899	1.607	1.419	1.308
19.50	25.768	15.857	19.657	13.832	31.50	4.378	3.531	3.152	2.865	43.50	1.865	1.577	1.394	1.283
19.75	24.015	15.231	18.257	13.235	31.75	4.262	3.464	3.073	2.811	43.75	1.831	1.548	1.368	1.258
20.00	22.469	14.665	17.026	12.694	32.00	4.154	3.399	2.999	2.760	44.00	1.797	1.520	1.343	1.234
20.25	21.123	14.146	15.955	12.196	32.25	4.053	3.338	2.930	2.711	44.25	1.764	1.492	1.318	1.210
20.50	19.953	13.668	15.025	11.736	32.50	3.959	3.279	2.866	2.664	44.50	1.732	1.465	1.294	1.187
20.75	18.930	13.227	14.211	11.312	32.75	3.870	3.223	2.806	2.620	44.75	1.700	1.438	1.269	1.164
21.00	18.027	12.825	13.492	10.921	33.00	3.787	3.169	2.749	2.577	45.00	1.669	1.412	1.246	1.142
21.25	17.221	12.459	12.850	10.565	33.25	3.709	3.117	2.695	2.535	45.25	1.638	1.387	1.222	1.121
21.50	16.496	12.131	12.271	10.242	33.50	3.635	3.067	2.645	2.495	45.50	1.608	1.362	1.199	1.100
21.75	15.840	11.840	11.746	9.952	33.75	3.565	3.018	2.596	2.456	45.75	1.579	1.338	1.177	1.079
22.00	15.243	11.585	11.268	9.695	34.00	3.498	2.971	2.551	2.419	46.00	1.550	1.314	1.155	1.059
22.25	14.699	11.364	10.832	9.467	34.25	3.435	2.925	2.507	2.382	46.25	1.522	1.291	1.133	1.040
22.50	14.204	11.173	10.434	9.267	34.50	3.374	2.880	2.465	2.346	46.50	1.494	1.268	1.112	1.020
22.75	13.754	11.008	10.071	9.090	34.75	3.316	2.836	2.425	2.311	46.75	1.467	1.246	1.091	1.002
23.00	13.347	10.862	9.742	8.930	35.00	3.260	2.793	2.386	2.277	47.00	1.441	1.224	1.071	0.984
23.25	12.978	10.724	9.442	8.779	35.25	3.206	2.751	2.349	2.244	47.25	1.415	1.203	1.051	0.966
23.50	12.645	10.583	9.172	8.628	35.50	3.154	2.710	2.313	2.211	47.50	1.390	1.183	1.032	0.949
23.75	12.346	10.425	8.927	8.466	35.75	3.103	2.669	2.278	2.178	47.75	1.365	1.163	1.013	0.932
24.00	12.075	10.236	8.705	8.284	36.00	3.054	2.629	2.243	2.146	48.00	1.341	1.143	0.995	0.916
24.25	11.830	10.006	8.503	8.073	36.25	3.006	2.589	2.210	2.114	48.25	1.318	1.124	0.977	0.900
24.50	11.606	9.729	8.317	7.830	36.50	2.959	2.550	2.178	2.083	48.50	1.295	1.105	0.960	0.884
24.75	11.395	9.406	8.143	7.555	36.75	2.914	2.511	2.146	2.052	48.75	1.272	1.087	0.942	0.869
25.00	11.192	9.045	7.976	7.254	37.00	2.869	2.473	2.115	2.021	49.00	1.250	1.069	0.926	0.855
25.25	10.988	8.657	7.811	6.936	37.25	2.825	2.435	2.084	1.991	49.25	1.229	1.052	0.910	0.840
25.50	10.775	8.256	7.641	6.611	37.50	2.782	2.397	2.054	1.961	49.50	1.208	1.035	0.894	0.826
25.75	10.545	7.855	7.461	6.289	37.75	2.739	2.360	2.024	1.931	49.75	1.188	1.018	0.879	0.813
26.00	10.290	7.465	7.268	5.977	38.00	2.697	2.322	1.995	1.901	50.00	1.168	1.002	0.864	0.799
26.25	10.008	7.093	7.057	5.682	38.25	2.656	2.285	1.966	1.871					

Table S8: Theoretical partial photoionization cross section for $8a_1$ Orbital. Energy in eV and cross section in MB.

E	SEP-L	SE-L	SEP-V	SE-V	E	SEP-L	SE-L	SEP-V	SE-V	E	SEP-L	SE-L	SEP-V	SE-V
15.00	11.074	11.856	8.297	10.034	26.75	15.382	9.979	10.332	7.638	38.50	3.121	2.621	2.206	2.062
15.25	11.213	12.235	8.352	10.345	27.00	14.684	9.459	9.859	7.239	38.75	3.063	2.572	2.168	2.026
15.50	11.384	13.050	8.436	11.078	27.25	13.971	8.982	9.378	6.873	39.00	3.006	2.525	2.132	1.990
15.75	11.538	14.681	8.528	12.606	27.50	13.262	8.546	8.901	6.538	39.25	2.951	2.478	2.095	1.955
16.00	11.817	17.293	8.710	15.054	27.75	12.574	8.145	8.438	6.231	39.50	2.896	2.431	2.060	1.920
16.25	12.203	19.465	8.981	17.033	28.00	11.915	7.777	7.997	5.949	39.75	2.842	2.386	2.025	1.886
16.50	12.735	19.461	9.371	16.920	28.25	11.294	7.437	7.581	5.688	40.00	2.789	2.341	1.990	1.852
16.75	13.461	18.629	9.919	16.014	28.50	10.713	7.123	7.193	5.448	40.25	2.737	2.296	1.956	1.818
17.00	14.425	18.104	10.657	15.402	28.75	10.173	6.832	6.832	5.226	40.50	2.686	2.252	1.923	1.784
17.25	15.595	17.969	11.557	15.150	29.00	9.671	6.563	6.498	5.020	40.75	2.636	2.209	1.889	1.750
17.50	16.795	18.035	12.471	15.102	29.25	9.206	6.312	6.190	4.828	41.00	2.586	2.167	1.856	1.717
17.75	17.734	18.167	13.162	15.121	29.50	8.776	6.079	5.904	4.650	41.25	2.537	2.125	1.823	1.684
18.00	18.244	18.294	13.500	15.145	29.75	8.378	5.862	5.640	4.485	41.50	2.489	2.084	1.791	1.652
18.25	18.416	18.403	13.562	15.163	30.00	8.009	5.659	5.396	4.330	41.75	2.441	2.043	1.759	1.620
18.50	18.450	18.494	13.514	15.177	30.25	7.667	5.470	5.170	4.186	42.00	2.395	2.003	1.727	1.588
18.75	18.482	18.579	13.465	15.191	30.50	7.348	5.293	4.960	4.052	42.25	2.348	1.964	1.695	1.557
19.00	18.559	18.655	13.454	15.203	30.75	7.052	5.127	4.765	3.926	42.50	2.303	1.926	1.664	1.526
19.25	18.679	18.713	13.481	15.204	31.00	6.777	4.972	4.584	3.808	42.75	2.258	1.888	1.633	1.495
19.50	18.824	18.743	13.530	15.184	31.25	6.520	4.827	4.415	3.698	43.00	2.215	1.851	1.602	1.465
19.75	18.976	18.736	13.589	15.136	31.50	6.280	4.691	4.258	3.595	43.25	2.171	1.815	1.571	1.436
20.00	19.120	18.691	13.646	15.054	31.75	6.056	4.563	4.111	3.499	43.50	2.129	1.780	1.541	1.407
20.25	19.245	18.612	13.692	14.944	32.00	5.847	4.443	3.974	3.408	43.75	2.087	1.745	1.512	1.378
20.50	19.341	18.507	13.720	14.812	32.25	5.652	4.330	3.846	3.323	44.00	2.046	1.711	1.482	1.351
20.75	19.402	18.383	13.724	14.664	32.50	5.469	4.223	3.727	3.242	44.25	2.006	1.678	1.453	1.323
21.00	19.423	18.264	13.701	14.520	32.75	5.297	4.122	3.615	3.166	44.50	1.967	1.646	1.425	1.297
21.25	19.405	18.151	13.650	14.383	33.00	5.137	4.026	3.510	3.095	44.75	1.928	1.614	1.397	1.271
21.50	19.351	18.065	13.574	14.265	33.25	4.986	3.935	3.412	3.027	45.00	1.891	1.583	1.369	1.245
21.75	19.267	18.007	13.476	14.171	33.50	4.845	3.849	3.320	2.963	45.25	1.854	1.553	1.342	1.220
22.00	19.163	17.979	13.365	14.103	33.75	4.712	3.767	3.233	2.901	45.50	1.818	1.524	1.315	1.196
22.25	19.048	17.980	13.245	14.060	34.00	4.587	3.688	3.152	2.843	45.75	1.782	1.495	1.289	1.172
22.50	18.933	18.001	13.126	14.034	34.25	4.469	3.612	3.075	2.787	46.00	1.748	1.467	1.264	1.149
22.75	18.825	18.029	13.013	14.017	34.50	4.358	3.540	3.003	2.734	46.25	1.714	1.440	1.238	1.126
23.00	18.732	18.044	12.910	13.993	34.75	4.253	3.470	2.935	2.682	46.50	1.681	1.413	1.214	1.104
23.25	18.655	18.019	12.821	13.941	35.00	4.153	3.403	2.870	2.633	46.75	1.649	1.387	1.190	1.083
23.50	18.597	17.922	12.746	13.837	35.25	4.059	3.337	2.809	2.585	47.00	1.618	1.362	1.167	1.062
23.75	18.555	17.723	12.684	13.659	35.50	3.969	3.274	2.751	2.539	47.25	1.587	1.337	1.144	1.041
24.00	18.524	17.396	12.632	13.388	35.75	3.883	3.213	2.695	2.494	47.50	1.557	1.313	1.121	1.021
24.25	18.495	16.934	12.583	13.016	36.00	3.800	3.153	2.642	2.451	47.75	1.528	1.289	1.099	1.002
24.50	18.458	16.343	12.532	12.550	36.25	3.722	3.095	2.591	2.408	48.00	1.500	1.266	1.078	0.983
24.75	18.398	15.651	12.467	12.009	36.50	3.646	3.038	2.543	2.367	48.25	1.472	1.244	1.057	0.964
25.00	18.298	14.891	12.377	11.420	36.75	3.573	2.982	2.496	2.326	48.50	1.445	1.222	1.037	0.946
25.25	18.139	14.102	12.251	10.810	37.00	3.503	2.928	2.450	2.287	48.75	1.419	1.201	1.017	0.929
25.50	17.903	13.315	12.075	10.203	37.25	3.434	2.874	2.407	2.248	49.00	1.393	1.180	0.998	0.912
25.75	17.577	12.554	11.841	9.617	37.50	3.368	2.822	2.364	2.209	49.25	1.368	1.159	0.979	0.895
26.00	17.154	11.835	11.545	9.064	37.75	3.304	2.770	2.323	2.172	49.50	1.344	1.140	0.961	0.879
26.25	16.638	11.165	11.189	8.549	38.00	3.242	2.720	2.283	2.135	49.75	1.320	1.120	0.943	0.863
26.50	16.041	10.547	10.780	8.074	38.25	3.181	2.670	2.244	2.098	50.00	1.296	1.101	0.926	0.848

Table S9: Theoretical partial photoionization cross section for $7a_1$ Orbital. Energy in eV and cross section in MB.

E	SEP-L	SE-L	SEP-V	SE-V	E	SEP-L	SE-L	SEP-V	SE-V	E	SEP-L	SE-L	SEP-V	SE-V
17.50	5.523	7.016	4.183	5.929	28.50	5.808	4.768	4.057	3.659	39.50	2.249	1.881	1.507	1.387
17.75	5.799	7.484	4.389	6.323	28.75	5.677	4.668	3.956	3.570	39.75	2.202	1.845	1.477	1.362
18.00	6.096	8.008	4.607	6.770	29.00	5.548	4.571	3.856	3.485	40.00	2.157	1.811	1.448	1.337
18.25	6.403	8.619	4.838	7.296	29.25	5.424	4.479	3.759	3.404	40.25	2.113	1.777	1.420	1.314
18.50	6.732	9.343	5.085	7.936	29.50	5.303	4.389	3.666	3.326	40.50	2.070	1.745	1.392	1.291
18.75	7.084	10.229	5.352	8.721	29.75	5.187	4.302	3.576	3.251	40.75	2.029	1.715	1.366	1.269
19.00	7.468	11.273	5.643	9.649	30.00	5.075	4.218	3.489	3.178	41.00	1.990	1.685	1.341	1.248
19.25	7.887	12.340	5.962	10.593	30.25	4.968	4.135	3.406	3.108	41.25	1.952	1.656	1.317	1.228
19.50	8.351	13.111	6.316	11.261	30.50	4.864	4.053	3.326	3.039	41.50	1.915	1.628	1.293	1.208
19.75	8.867	13.306	6.708	11.401	30.75	4.764	3.973	3.249	2.972	41.75	1.879	1.601	1.270	1.189
20.00	9.436	12.972	7.140	11.065	31.00	4.667	3.894	3.175	2.907	42.00	1.845	1.575	1.249	1.170
20.25	10.048	12.369	7.603	10.494	31.25	4.574	3.817	3.104	2.844	42.25	1.811	1.550	1.227	1.152
20.50	10.670	11.712	8.069	9.886	31.50	4.482	3.740	3.036	2.781	42.50	1.779	1.526	1.207	1.135
20.75	11.245	11.105	8.492	9.331	31.75	4.393	3.664	2.969	2.720	42.75	1.749	1.502	1.187	1.118
21.00	11.693	10.578	8.813	8.852	32.00	4.305	3.589	2.904	2.660	43.00	1.719	1.479	1.168	1.102
21.25	11.949	10.129	8.981	8.446	32.25	4.219	3.515	2.841	2.601	43.25	1.690	1.457	1.150	1.086
21.50	11.989	9.744	8.982	8.099	32.50	4.135	3.442	2.780	2.544	43.50	1.662	1.436	1.132	1.071
21.75	11.840	9.407	8.839	7.796	32.75	4.052	3.369	2.720	2.487	43.75	1.635	1.415	1.115	1.056
22.00	11.559	9.107	8.599	7.527	33.00	3.969	3.298	2.661	2.432	44.00	1.609	1.394	1.098	1.041
22.25	11.206	8.834	8.307	7.282	33.25	3.888	3.228	2.603	2.377	44.25	1.584	1.375	1.082	1.027
22.50	10.827	8.581	8.000	7.055	33.50	3.808	3.158	2.547	2.324	44.50	1.559	1.355	1.067	1.013
22.75	10.451	8.345	7.699	6.842	33.75	3.729	3.090	2.491	2.272	44.75	1.535	1.337	1.051	1.000
23.00	10.093	8.121	7.414	6.641	34.00	3.651	3.022	2.437	2.221	45.00	1.512	1.318	1.037	0.987
23.25	9.760	7.909	7.149	6.451	34.25	3.574	2.956	2.384	2.171	45.25	1.490	1.300	1.022	0.974
23.50	9.451	7.707	6.905	6.269	34.50	3.498	2.891	2.332	2.122	45.50	1.468	1.283	1.008	0.961
23.75	9.166	7.513	6.680	6.095	34.75	3.423	2.827	2.280	2.074	45.75	1.447	1.266	0.995	0.949
24.00	8.903	7.328	6.472	5.929	35.00	3.349	2.765	2.230	2.028	46.00	1.427	1.249	0.982	0.937
24.25	8.657	7.149	6.279	5.770	35.25	3.277	2.703	2.181	1.982	46.25	1.407	1.233	0.969	0.925
24.50	8.428	6.977	6.098	5.616	35.50	3.205	2.643	2.133	1.938	46.50	1.387	1.217	0.956	0.914
24.75	8.212	6.811	5.928	5.468	35.75	3.135	2.585	2.086	1.895	46.75	1.368	1.202	0.944	0.903
25.00	8.009	6.649	5.768	5.325	36.00	3.066	2.528	2.040	1.853	47.00	1.350	1.187	0.932	0.891
25.25	7.815	6.492	5.616	5.186	36.25	2.998	2.472	1.995	1.812	47.25	1.332	1.172	0.920	0.881
25.50	7.631	6.339	5.471	5.051	36.50	2.932	2.418	1.951	1.773	47.50	1.314	1.157	0.909	0.870
25.75	7.454	6.188	5.333	4.919	36.75	2.867	2.365	1.908	1.734	47.75	1.297	1.143	0.898	0.859
26.00	7.284	6.041	5.200	4.789	37.00	2.803	2.313	1.866	1.697	48.00	1.280	1.129	0.887	0.849
26.25	7.120	5.896	5.071	4.662	37.25	2.741	2.263	1.825	1.661	48.25	1.264	1.115	0.876	0.839
26.50	6.961	5.753	4.948	4.536	37.50	2.680	2.215	1.785	1.626	48.50	1.248	1.101	0.865	0.829
26.75	6.806	5.614	4.827	4.413	37.75	2.621	2.168	1.747	1.593	48.75	1.232	1.088	0.855	0.819
27.00	6.655	5.478	4.710	4.293	38.00	2.563	2.123	1.709	1.560	49.00	1.217	1.075	0.845	0.810
27.25	6.507	5.346	4.596	4.176	38.25	2.507	2.079	1.673	1.529	49.25	1.201	1.062	0.835	0.800
27.50	6.363	5.219	4.484	4.063	38.50	2.452	2.036	1.638	1.498	49.50	1.187	1.050	0.825	0.791
27.75	6.220	5.098	4.375	3.954	38.75	2.399	1.995	1.603	1.469	49.75	1.172	1.037	0.815	0.782
28.00	6.080	4.983	4.267	3.851	39.00	2.348	1.956	1.570	1.441	50.00	1.158	1.025	0.806	0.773
28.25	5.943	4.873	4.161	3.753	39.25	2.298	1.918	1.538	1.413					

Table S10: Theoretical partial photoionization cross section for $4b_2$ Orbital. Energy in eV and cross section in MB.

E	SEP-L	SE-L	SEP-V	SE-V	E	SEP-L	SE-L	SEP-V	SE-V	E	SEP-L	SE-L	SEP-V	SE-V
18.25	6.621	4.221	5.298	3.774	29.00	5.807	5.248	4.173	4.175	39.75	2.622	2.157	1.866	1.682
18.50	5.831	4.287	4.647	3.819	29.25	5.773	5.181	4.142	4.114	40.00	2.561	2.115	1.823	1.650
18.75	5.338	4.351	4.234	3.863	29.50	5.734	5.111	4.107	4.052	40.25	2.502	2.076	1.783	1.619
19.00	5.015	4.425	3.962	3.919	29.75	5.689	5.040	4.068	3.989	40.50	2.446	2.038	1.744	1.590
19.25	4.798	4.529	3.774	4.006	30.00	5.639	4.966	4.027	3.924	40.75	2.392	2.002	1.707	1.562
19.50	4.652	4.689	3.644	4.153	30.25	5.584	4.891	3.982	3.860	41.00	2.341	1.968	1.672	1.536
19.75	4.557	4.923	3.556	4.371	30.50	5.524	4.815	3.934	3.794	41.25	2.292	1.935	1.638	1.511
20.00	4.502	5.200	3.501	4.622	30.75	5.461	4.738	3.885	3.729	41.50	2.246	1.904	1.606	1.487
20.25	4.482	5.446	3.476	4.830	31.00	5.394	4.660	3.833	3.663	41.75	2.201	1.874	1.575	1.464
20.50	4.497	5.610	3.481	4.951	31.25	5.324	4.581	3.779	3.596	42.00	2.159	1.846	1.546	1.442
20.75	4.549	5.697	3.519	4.998	31.50	5.252	4.501	3.725	3.530	42.25	2.119	1.819	1.518	1.420
21.00	4.639	5.734	3.589	5.000	31.75	5.178	4.421	3.669	3.463	42.50	2.081	1.793	1.492	1.400
21.25	4.766	5.744	3.687	4.979	32.00	5.101	4.340	3.612	3.397	42.75	2.044	1.767	1.466	1.381
21.50	4.917	5.741	3.803	4.949	32.25	5.023	4.258	3.554	3.330	43.00	2.009	1.743	1.442	1.362
21.75	5.075	5.733	3.922	4.918	32.50	4.944	4.177	3.496	3.264	43.25	1.976	1.720	1.419	1.344
22.00	5.224	5.724	4.028	4.888	32.75	4.864	4.095	3.437	3.198	43.50	1.945	1.698	1.397	1.326
22.25	5.352	5.717	4.115	4.860	33.00	4.782	4.013	3.378	3.132	43.75	1.914	1.676	1.376	1.309
22.50	5.456	5.709	4.181	4.837	33.25	4.700	3.931	3.319	3.066	44.00	1.885	1.655	1.356	1.293
22.75	5.538	5.705	4.227	4.816	33.50	4.617	3.848	3.259	3.001	44.25	1.858	1.635	1.337	1.277
23.00	5.601	5.703	4.259	4.799	33.75	4.533	3.766	3.199	2.935	44.50	1.831	1.615	1.318	1.262
23.25	5.649	5.704	4.280	4.784	34.00	4.449	3.684	3.139	2.871	44.75	1.805	1.596	1.300	1.247
23.50	5.687	5.705	4.294	4.773	34.25	4.365	3.603	3.079	2.806	45.00	1.781	1.577	1.283	1.232
23.75	5.718	5.706	4.302	4.762	34.50	4.280	3.522	3.019	2.742	45.25	1.757	1.559	1.266	1.218
24.00	5.743	5.710	4.308	4.752	34.75	4.195	3.441	2.959	2.679	45.50	1.734	1.542	1.250	1.204
24.25	5.766	5.712	4.312	4.746	35.00	4.110	3.361	2.900	2.616	45.75	1.712	1.524	1.235	1.190
24.50	5.785	5.723	4.314	4.739	35.25	4.025	3.281	2.840	2.554	46.00	1.691	1.507	1.220	1.177
24.75	5.803	5.727	4.316	4.728	35.50	3.939	3.203	2.780	2.493	46.25	1.670	1.491	1.205	1.164
25.00	5.820	5.726	4.317	4.719	35.75	3.854	3.126	2.721	2.432	46.50	1.650	1.475	1.191	1.151
25.25	5.835	5.729	4.318	4.709	36.00	3.769	3.050	2.661	2.373	46.75	1.631	1.459	1.178	1.138
25.50	5.849	5.728	4.319	4.697	36.25	3.685	2.976	2.602	2.315	47.00	1.612	1.444	1.164	1.126
25.75	5.862	5.724	4.319	4.683	36.50	3.601	2.903	2.544	2.259	47.25	1.594	1.428	1.151	1.114
26.00	5.874	5.716	4.318	4.666	36.75	3.517	2.832	2.486	2.204	47.50	1.576	1.413	1.138	1.102
26.25	5.884	5.705	4.317	4.646	37.00	3.434	2.763	2.428	2.151	47.75	1.558	1.399	1.126	1.090
26.50	5.893	5.690	4.314	4.622	37.25	3.352	2.696	2.371	2.099	48.00	1.541	1.384	1.114	1.079
26.75	5.900	5.669	4.311	4.595	37.50	3.271	2.632	2.315	2.049	48.25	1.524	1.370	1.102	1.067
27.00	5.904	5.644	4.305	4.564	37.75	3.192	2.570	2.260	2.001	48.50	1.508	1.356	1.090	1.056
27.25	5.906	5.613	4.298	4.529	38.00	3.114	2.510	2.206	1.954	48.75	1.492	1.342	1.078	1.045
27.50	5.905	5.576	4.289	4.489	38.25	3.037	2.452	2.153	1.910	49.00	1.476	1.328	1.067	1.034
27.75	5.899	5.534	4.277	4.445	38.50	2.963	2.397	2.101	1.867	49.25	1.461	1.315	1.056	1.024
28.00	5.890	5.486	4.263	4.397	38.75	2.890	2.344	2.051	1.827	49.50	1.446	1.302	1.045	1.013
28.25	5.877	5.432	4.245	4.346	39.00	2.819	2.294	2.002	1.788	49.75	1.431	1.289	1.034	1.003
28.50	5.859	5.375	4.225	4.291	39.25	2.751	2.246	1.955	1.751	50.00	1.416	1.276	1.024	0.992
28.75	5.836	5.313	4.200	4.234	39.50	2.685	2.200	1.909	1.716					

Table S11: Theoretical partial photoionization cross section for $6a_1$ Orbital. Energy in eV and cross section in MB.

E	SEP-L	SE-L	SEP-V	SE-V	E	SEP-L	SE-L	SEP-V	SE-V	E	SEP-L	SE-L	SEP-V	SE-V
19.00	4.190	4.682	3.350	4.100	29.50	5.015	4.773	3.638	3.790	40.00	2.959	2.474	2.051	1.887
19.25	4.295	4.950	3.411	4.323	29.75	5.005	4.728	3.624	3.744	40.25	2.900	2.428	2.012	1.853
19.50	4.415	5.284	3.493	4.609	30.00	4.995	4.680	3.610	3.695	40.50	2.842	2.383	1.973	1.821
19.75	4.556	5.740	3.588	5.010	30.25	4.983	4.629	3.593	3.645	40.75	2.786	2.339	1.936	1.790
20.00	4.720	6.391	3.708	5.593	30.50	4.968	4.577	3.574	3.595	41.00	2.731	2.298	1.900	1.760
20.25	4.912	7.243	3.850	6.364	30.75	4.948	4.525	3.552	3.545	41.25	2.677	2.258	1.865	1.731
20.50	5.137	8.017	4.019	7.062	31.00	4.922	4.472	3.526	3.495	41.50	2.625	2.219	1.831	1.703
20.75	5.404	8.228	4.221	7.236	31.25	4.891	4.419	3.495	3.446	41.75	2.575	2.182	1.799	1.676
21.00	5.716	7.891	4.461	6.903	31.50	4.855	4.367	3.462	3.397	42.00	2.526	2.146	1.767	1.651
21.25	6.072	7.404	4.737	6.435	31.75	4.815	4.315	3.426	3.350	42.25	2.479	2.111	1.737	1.626
21.50	6.450	6.990	5.032	6.037	32.00	4.771	4.263	3.387	3.303	42.50	2.434	2.077	1.708	1.602
21.75	6.800	6.685	5.305	5.743	32.25	4.725	4.211	3.347	3.257	42.75	2.390	2.045	1.679	1.578
22.00	7.050	6.466	5.496	5.529	32.50	4.676	4.160	3.306	3.211	43.00	2.348	2.013	1.652	1.556
22.25	7.144	6.307	5.561	5.371	32.75	4.626	4.108	3.265	3.166	43.25	2.307	1.982	1.626	1.534
22.50	7.084	6.185	5.501	5.249	33.00	4.576	4.057	3.223	3.121	43.50	2.268	1.953	1.601	1.512
22.75	6.924	6.088	5.359	5.149	33.25	4.524	4.005	3.181	3.076	43.75	2.230	1.924	1.577	1.491
23.00	6.727	6.004	5.188	5.064	33.50	4.473	3.953	3.140	3.031	44.00	2.194	1.895	1.553	1.470
23.25	6.535	5.929	5.021	4.988	33.75	4.420	3.900	3.098	2.986	44.25	2.158	1.868	1.530	1.450
23.50	6.367	5.857	4.875	4.916	34.00	4.368	3.846	3.057	2.941	44.50	2.124	1.841	1.508	1.430
23.75	6.228	5.786	4.753	4.843	34.25	4.316	3.792	3.016	2.896	44.75	2.091	1.814	1.487	1.411
24.00	6.114	5.714	4.651	4.773	34.50	4.263	3.737	2.975	2.851	45.00	2.059	1.788	1.466	1.392
24.25	6.019	5.641	4.565	4.701	34.75	4.210	3.681	2.935	2.805	45.25	2.027	1.763	1.446	1.373
24.50	5.938	5.567	4.492	4.628	35.00	4.156	3.624	2.894	2.759	45.50	1.997	1.738	1.426	1.354
24.75	5.866	5.493	4.426	4.556	35.25	4.102	3.566	2.853	2.712	45.75	1.967	1.714	1.406	1.336
25.00	5.800	5.420	4.365	4.485	35.50	4.048	3.508	2.812	2.665	46.00	1.938	1.690	1.387	1.318
25.25	5.737	5.349	4.308	4.416	35.75	3.992	3.448	2.771	2.618	46.25	1.910	1.666	1.368	1.300
25.50	5.676	5.281	4.252	4.350	36.00	3.936	3.388	2.730	2.571	46.50	1.882	1.643	1.350	1.283
25.75	5.615	5.217	4.197	4.288	36.25	3.879	3.328	2.688	2.524	46.75	1.855	1.621	1.332	1.266
26.00	5.555	5.158	4.143	4.231	36.50	3.821	3.267	2.646	2.477	47.00	1.829	1.598	1.314	1.249
26.25	5.496	5.106	4.089	4.179	36.75	3.763	3.205	2.604	2.430	47.25	1.803	1.577	1.297	1.232
26.50	5.438	5.060	4.037	4.133	37.00	3.703	3.144	2.561	2.383	47.50	1.777	1.555	1.280	1.216
26.75	5.382	5.021	3.987	4.094	37.25	3.642	3.083	2.518	2.336	47.75	1.752	1.535	1.263	1.200
27.00	5.328	4.990	3.938	4.061	37.50	3.581	3.022	2.475	2.290	48.00	1.728	1.514	1.246	1.184
27.25	5.277	4.966	3.892	4.034	37.75	3.519	2.962	2.432	2.245	48.25	1.704	1.494	1.230	1.168
27.50	5.229	4.948	3.849	4.012	38.00	3.457	2.903	2.389	2.201	48.50	1.680	1.475	1.213	1.153
27.75	5.185	4.934	3.810	3.993	38.25	3.394	2.845	2.345	2.158	48.75	1.657	1.456	1.197	1.138
28.00	5.146	4.923	3.774	3.975	38.50	3.331	2.787	2.302	2.115	49.00	1.635	1.437	1.182	1.123
28.25	5.112	4.911	3.742	3.955	38.75	3.268	2.731	2.259	2.074	49.25	1.613	1.419	1.166	1.109
28.50	5.083	4.895	3.714	3.933	39.00	3.205	2.677	2.216	2.034	49.50	1.591	1.401	1.151	1.095
28.75	5.059	4.874	3.691	3.905	39.25	3.143	2.624	2.174	1.995	49.75	1.570	1.384	1.136	1.081
29.00	5.041	4.847	3.670	3.872	39.50	3.081	2.572	2.132	1.958	50.00	1.549	1.367	1.122	1.068
29.25	5.026	4.812	3.653	3.833	39.75	3.020	2.522	2.091	1.921					

Table S12: Theoretical partial photoionization cross section for $3b_2$ Orbital. Energy in eV and cross section in MB.

E	SEP-L	SE-L	SEP-V	SE-V	E	SEP-L	SE-L	SEP-V	SE-V	E	SEP-L	SE-L	SEP-V	SE-V
22.50	0.682	0.708	0.550	0.621	31.75	1.104	1.304	0.953	1.212	41.00	2.172	2.200	1.679	1.842
22.75	0.686	0.696	0.549	0.606	32.00	1.124	1.345	0.966	1.241	41.25	2.190	2.208	1.692	1.849
23.00	0.688	0.682	0.547	0.592	32.25	1.149	1.383	0.983	1.268	41.50	2.207	2.215	1.705	1.854
23.25	0.688	0.672	0.544	0.585	32.50	1.178	1.418	1.003	1.292	41.75	2.222	2.220	1.716	1.859
23.50	0.687	0.675	0.540	0.595	32.75	1.211	1.451	1.027	1.314	42.00	2.236	2.224	1.727	1.863
23.75	0.684	0.707	0.536	0.637	33.00	1.248	1.482	1.052	1.333	42.25	2.249	2.227	1.737	1.865
24.00	0.679	0.762	0.533	0.700	33.25	1.287	1.511	1.080	1.352	42.50	2.260	2.228	1.746	1.867
24.25	0.676	0.804	0.531	0.745	33.50	1.328	1.540	1.108	1.370	42.75	2.270	2.228	1.753	1.867
24.50	0.674	0.819	0.532	0.761	33.75	1.369	1.567	1.136	1.387	43.00	2.278	2.227	1.760	1.867
24.75	0.677	0.820	0.538	0.761	34.00	1.409	1.594	1.164	1.404	43.25	2.285	2.225	1.766	1.866
25.00	0.687	0.816	0.551	0.758	34.25	1.448	1.621	1.189	1.422	43.50	2.290	2.221	1.771	1.864
25.25	0.704	0.814	0.572	0.758	34.50	1.485	1.647	1.214	1.439	43.75	2.294	2.217	1.775	1.861
25.50	0.725	0.817	0.596	0.762	34.75	1.520	1.674	1.236	1.457	44.00	2.297	2.211	1.777	1.857
25.75	0.746	0.824	0.619	0.773	35.00	1.553	1.700	1.258	1.474	44.25	2.298	2.204	1.779	1.852
26.00	0.763	0.838	0.639	0.790	35.25	1.585	1.726	1.278	1.492	44.50	2.298	2.196	1.780	1.847
26.25	0.777	0.857	0.655	0.813	35.50	1.616	1.752	1.298	1.510	44.75	2.296	2.187	1.780	1.840
26.50	0.789	0.880	0.669	0.839	35.75	1.646	1.778	1.317	1.528	45.00	2.294	2.177	1.779	1.833
26.75	0.801	0.907	0.684	0.869	36.00	1.675	1.804	1.336	1.546	45.25	2.290	2.166	1.778	1.825
27.00	0.815	0.934	0.700	0.899	36.25	1.703	1.830	1.354	1.564	45.50	2.285	2.154	1.775	1.816
27.25	0.832	0.960	0.719	0.928	36.50	1.732	1.855	1.373	1.582	45.75	2.278	2.140	1.772	1.806
27.50	0.852	0.984	0.740	0.953	36.75	1.759	1.880	1.391	1.601	46.00	2.271	2.126	1.767	1.795
27.75	0.874	1.003	0.763	0.973	37.00	1.787	1.905	1.409	1.618	46.25	2.263	2.110	1.762	1.783
28.00	0.898	1.018	0.788	0.989	37.25	1.814	1.929	1.427	1.636	46.50	2.253	2.094	1.756	1.770
28.25	0.923	1.028	0.812	1.000	37.50	1.841	1.953	1.445	1.654	46.75	2.242	2.076	1.749	1.756
28.50	0.946	1.036	0.835	1.007	37.75	1.868	1.977	1.463	1.671	47.00	2.230	2.057	1.742	1.741
28.75	0.968	1.041	0.857	1.011	38.00	1.894	2.000	1.481	1.688	47.25	2.218	2.037	1.733	1.725
29.00	0.988	1.046	0.875	1.014	38.25	1.920	2.022	1.499	1.705	47.50	2.204	2.017	1.724	1.708
29.25	1.004	1.051	0.890	1.017	38.50	1.946	2.044	1.517	1.721	47.75	2.188	1.995	1.714	1.691
29.50	1.018	1.058	0.901	1.021	38.75	1.971	2.064	1.535	1.737	48.00	2.172	1.972	1.702	1.673
29.75	1.028	1.067	0.909	1.028	39.00	1.996	2.084	1.552	1.752	48.25	2.155	1.949	1.690	1.654
30.00	1.037	1.080	0.915	1.038	39.25	2.020	2.103	1.569	1.766	48.50	2.137	1.925	1.677	1.634
30.25	1.044	1.098	0.920	1.052	39.50	2.044	2.121	1.586	1.780	48.75	2.117	1.900	1.664	1.614
30.50	1.050	1.122	0.923	1.070	39.75	2.068	2.137	1.603	1.793	49.00	2.097	1.875	1.649	1.593
30.75	1.057	1.151	0.926	1.093	40.00	2.090	2.152	1.619	1.805	49.25	2.076	1.850	1.634	1.572
31.00	1.065	1.185	0.930	1.120	40.25	2.112	2.166	1.635	1.815	49.50	2.053	1.824	1.617	1.551
31.25	1.075	1.223	0.936	1.150	40.50	2.133	2.179	1.650	1.825	49.75	2.030	1.798	1.600	1.529
31.50	1.088	1.263	0.943	1.181	40.75	2.153	2.190	1.665	1.834	50.00	2.007	1.772	1.583	1.507

Table S13: Theoretical partial photoionization cross section for $5a_1$ Orbital. Energy in eV and cross section in MB.

E	SEP-L	SE-L	SEP-V	SE-V	E	SEP-L	SE-L	SEP-V	SE-V	E	SEP-L	SE-L	SEP-V	SE-V
24.00	0.718	0.817	0.565	0.697	32.75	1.074	1.216	0.882	1.083	41.50	1.736	1.687	1.345	1.417
24.25	0.737	0.825	0.576	0.700	33.00	1.088	1.250	0.891	1.110	41.75	1.738	1.684	1.347	1.414
24.50	0.753	0.831	0.586	0.706	33.25	1.105	1.283	0.901	1.135	42.00	1.740	1.681	1.348	1.411
24.75	0.766	0.841	0.594	0.724	33.50	1.124	1.314	0.915	1.159	42.25	1.741	1.677	1.349	1.408
25.00	0.777	0.872	0.602	0.772	33.75	1.147	1.344	0.931	1.181	42.50	1.742	1.673	1.349	1.404
25.25	0.787	0.922	0.610	0.838	34.00	1.173	1.371	0.949	1.201	42.75	1.741	1.668	1.349	1.400
25.50	0.795	0.950	0.618	0.865	34.25	1.203	1.396	0.971	1.219	43.00	1.740	1.662	1.348	1.396
25.75	0.803	0.946	0.628	0.858	34.50	1.234	1.419	0.994	1.236	43.25	1.738	1.657	1.347	1.391
26.00	0.812	0.930	0.642	0.842	34.75	1.268	1.441	1.018	1.251	43.50	1.735	1.650	1.345	1.386
26.25	0.825	0.914	0.660	0.827	35.00	1.302	1.462	1.042	1.265	43.75	1.732	1.644	1.343	1.381
26.50	0.840	0.900	0.682	0.817	35.25	1.335	1.482	1.066	1.279	44.00	1.728	1.637	1.340	1.376
26.75	0.854	0.889	0.703	0.810	35.50	1.367	1.501	1.089	1.292	44.25	1.724	1.630	1.338	1.370
27.00	0.865	0.882	0.717	0.806	35.75	1.398	1.519	1.111	1.305	44.50	1.719	1.622	1.334	1.364
27.25	0.869	0.880	0.724	0.807	36.00	1.426	1.536	1.131	1.317	44.75	1.714	1.614	1.331	1.358
27.50	0.869	0.881	0.726	0.810	36.25	1.453	1.552	1.150	1.328	45.00	1.708	1.606	1.327	1.351
27.75	0.867	0.887	0.726	0.818	36.50	1.478	1.568	1.167	1.339	45.25	1.702	1.598	1.322	1.345
28.00	0.866	0.896	0.725	0.828	36.75	1.501	1.583	1.183	1.349	45.50	1.695	1.589	1.318	1.338
28.25	0.866	0.910	0.726	0.841	37.00	1.523	1.596	1.198	1.359	45.75	1.688	1.580	1.313	1.331
28.50	0.868	0.925	0.729	0.856	37.25	1.544	1.609	1.213	1.368	46.00	1.681	1.571	1.308	1.324
28.75	0.874	0.942	0.734	0.872	37.50	1.564	1.621	1.226	1.376	46.25	1.673	1.562	1.303	1.316
29.00	0.882	0.959	0.742	0.887	37.75	1.582	1.632	1.239	1.383	46.50	1.665	1.552	1.297	1.309
29.25	0.892	0.975	0.751	0.901	38.00	1.599	1.642	1.251	1.390	46.75	1.657	1.542	1.292	1.301
29.50	0.905	0.990	0.762	0.913	38.25	1.616	1.651	1.262	1.396	47.00	1.648	1.532	1.286	1.293
29.75	0.920	1.003	0.774	0.924	38.50	1.631	1.659	1.272	1.402	47.25	1.639	1.521	1.280	1.284
30.00	0.936	1.014	0.787	0.932	38.75	1.645	1.666	1.282	1.407	47.50	1.630	1.510	1.273	1.275
30.25	0.952	1.024	0.800	0.939	39.00	1.658	1.673	1.291	1.411	47.75	1.621	1.498	1.267	1.266
30.50	0.967	1.034	0.812	0.946	39.25	1.671	1.678	1.299	1.414	48.00	1.611	1.486	1.260	1.257
30.75	0.982	1.044	0.823	0.952	39.50	1.682	1.682	1.307	1.417	48.25	1.601	1.474	1.253	1.247
31.00	0.996	1.055	0.833	0.960	39.75	1.692	1.686	1.314	1.419	48.50	1.591	1.462	1.245	1.237
31.25	1.009	1.068	0.842	0.968	40.00	1.701	1.688	1.321	1.420	48.75	1.580	1.449	1.238	1.226
31.50	1.020	1.084	0.849	0.980	40.25	1.709	1.690	1.326	1.421	49.00	1.569	1.436	1.230	1.215
31.75	1.031	1.103	0.856	0.994	40.50	1.716	1.691	1.331	1.421	49.25	1.557	1.422	1.222	1.204
32.00	1.041	1.126	0.862	1.012	40.75	1.723	1.691	1.336	1.421	49.50	1.546	1.408	1.213	1.193
32.25	1.051	1.153	0.868	1.033	41.00	1.728	1.690	1.339	1.420	49.75	1.533	1.394	1.204	1.181
32.50	1.062	1.183	0.874	1.057	41.25	1.732	1.689	1.342	1.418					

Table S14: Theoretical partial photoionization cross section for $4a_1$ Orbital. Energy in eV and cross section in MB.

E	SEP-L	SE-L	SEP-V	SE-V	E	SEP-L	SE-L	SEP-V	SE-V	E	SEP-L	SE-L	SEP-V	SE-V
29.75	0.223	0.198	0.244	0.194	36.75	0.705	0.648	0.745	0.632	43.75	0.775	0.786	0.765	0.728
30.00	0.240	0.204	0.277	0.197	37.00	0.713	0.658	0.754	0.642	44.00	0.777	0.788	0.765	0.729
30.25	0.316	0.209	0.418	0.201	37.25	0.721	0.668	0.762	0.651	44.25	0.779	0.790	0.766	0.729
30.50	0.453	0.215	0.629	0.204	37.50	0.729	0.677	0.769	0.660	44.50	0.781	0.792	0.766	0.729
30.75	0.441	0.220	0.567	0.209	37.75	0.735	0.686	0.775	0.669	44.75	0.783	0.794	0.766	0.729
31.00	0.425	0.227	0.517	0.217	38.00	0.740	0.696	0.779	0.677	45.00	0.785	0.796	0.766	0.729
31.25	0.425	0.238	0.497	0.233	38.25	0.744	0.705	0.781	0.686	45.25	0.787	0.798	0.766	0.730
31.50	0.430	0.259	0.489	0.266	38.50	0.746	0.714	0.781	0.694	45.50	0.788	0.800	0.767	0.730
31.75	0.438	0.299	0.487	0.322	38.75	0.746	0.723	0.780	0.702	45.75	0.790	0.802	0.767	0.730
32.00	0.448	0.348	0.490	0.382	39.00	0.746	0.732	0.778	0.709	46.00	0.792	0.804	0.767	0.730
32.25	0.459	0.382	0.497	0.415	39.25	0.746	0.740	0.775	0.716	46.25	0.794	0.806	0.767	0.731
32.50	0.473	0.402	0.507	0.427	39.50	0.745	0.748	0.772	0.722	46.50	0.796	0.808	0.767	0.731
32.75	0.489	0.416	0.521	0.433	39.75	0.745	0.754	0.770	0.727	46.75	0.797	0.810	0.767	0.731
33.00	0.506	0.428	0.537	0.438	40.00	0.745	0.759	0.768	0.731	47.00	0.799	0.812	0.767	0.731
33.25	0.525	0.441	0.555	0.445	40.25	0.746	0.763	0.766	0.733	47.25	0.800	0.814	0.767	0.732
33.50	0.544	0.454	0.575	0.454	40.50	0.747	0.766	0.765	0.735	47.50	0.802	0.815	0.767	0.732
33.75	0.563	0.468	0.595	0.464	40.75	0.748	0.768	0.764	0.735	47.75	0.803	0.817	0.767	0.732
34.00	0.582	0.483	0.614	0.477	41.00	0.750	0.770	0.763	0.735	48.00	0.805	0.819	0.767	0.732
34.25	0.599	0.499	0.632	0.491	41.25	0.752	0.771	0.763	0.734	48.25	0.806	0.820	0.767	0.733
34.50	0.615	0.516	0.649	0.506	41.50	0.754	0.772	0.763	0.733	48.50	0.807	0.822	0.767	0.733
34.75	0.629	0.533	0.664	0.522	41.75	0.756	0.773	0.763	0.732	48.75	0.808	0.824	0.767	0.733
35.00	0.641	0.550	0.677	0.538	42.00	0.759	0.774	0.763	0.731	49.00	0.809	0.825	0.766	0.733
35.25	0.651	0.567	0.688	0.555	42.25	0.761	0.775	0.763	0.730	49.25	0.810	0.826	0.766	0.733
35.50	0.661	0.584	0.699	0.570	42.50	0.763	0.777	0.764	0.729	49.50	0.811	0.828	0.766	0.733
35.75	0.670	0.599	0.709	0.585	42.75	0.766	0.778	0.764	0.729	49.75	0.812	0.829	0.765	0.734
36.00	0.679	0.612	0.718	0.598	43.00	0.768	0.780	0.764	0.729	50.00	0.812	0.830	0.765	0.734
36.25	0.687	0.625	0.727	0.611	43.25	0.770	0.782	0.764	0.728					
36.50	0.696	0.637	0.736	0.622	43.50	0.772	0.784	0.765	0.728					

Table S15: Theoretical Assymetric Parameter for $1a_2$ Orbital. Energy in eV.

E	SEP-L	SE-L	SEP-V	SE-V	E	SEP-L	SE-L	SEP-V	SE-V	E	SEP-L	SE-L	SEP-V	SE-V
8.25	-0.127	-0.571	-0.144	-0.609	22.25	0.941	0.942	0.884	0.901	36.25	1.342	1.335	1.265	1.278
8.50	-0.165	-0.588	-0.184	-0.625	22.50	0.949	0.949	0.893	0.908	36.50	1.348	1.342	1.271	1.284
8.75	-0.162	-0.592	-0.176	-0.626	22.75	0.957	0.956	0.901	0.916	36.75	1.355	1.349	1.277	1.290
9.00	-0.165	-0.582	-0.175	-0.611	23.00	0.965	0.963	0.909	0.923	37.00	1.361	1.355	1.283	1.296
9.25	-0.181	-0.560	-0.190	-0.581	23.25	0.973	0.970	0.917	0.930	37.25	1.368	1.362	1.289	1.301
9.50	-0.206	-0.519	-0.218	-0.530	23.50	0.980	0.977	0.924	0.938	37.50	1.374	1.368	1.295	1.307
9.75	-0.235	-0.452	-0.250	-0.454	23.75	0.988	0.983	0.932	0.945	37.75	1.380	1.375	1.300	1.313
10.00	-0.261	-0.371	-0.277	-0.368	24.00	0.995	0.990	0.940	0.952	38.00	1.387	1.381	1.306	1.319
10.25	-0.279	-0.294	-0.292	-0.287	24.25	1.003	0.997	0.947	0.959	38.25	1.393	1.388	1.312	1.324
10.50	-0.286	-0.222	-0.293	-0.214	24.50	1.010	1.003	0.954	0.966	38.50	1.399	1.394	1.317	1.330
10.75	-0.279	-0.156	-0.276	-0.146	24.75	1.017	1.010	0.962	0.973	38.75	1.405	1.400	1.323	1.336
11.00	-0.255	-0.094	-0.242	-0.084	25.00	1.024	1.017	0.969	0.980	39.00	1.411	1.407	1.329	1.341
11.25	-0.213	-0.034	-0.191	-0.025	25.25	1.031	1.024	0.976	0.987	39.25	1.417	1.413	1.334	1.347
11.50	-0.160	0.022	-0.131	0.031	25.50	1.038	1.030	0.983	0.994	39.50	1.424	1.419	1.340	1.353
11.75	-0.101	0.076	-0.069	0.083	25.75	1.045	1.037	0.990	1.001	39.75	1.429	1.425	1.345	1.358
12.00	-0.042	0.126	-0.010	0.131	26.00	1.052	1.044	0.997	1.008	40.00	1.435	1.431	1.350	1.364
12.25	0.015	0.175	0.046	0.177	26.25	1.060	1.051	1.004	1.015	40.25	1.441	1.437	1.356	1.369
12.50	0.068	0.220	0.098	0.220	26.50	1.067	1.058	1.011	1.022	40.50	1.447	1.443	1.361	1.375
12.75	0.119	0.264	0.146	0.261	26.75	1.074	1.065	1.018	1.029	40.75	1.453	1.449	1.367	1.380
13.00	0.167	0.304	0.190	0.299	27.00	1.081	1.072	1.025	1.036	41.00	1.459	1.455	1.372	1.385
13.25	0.213	0.343	0.232	0.334	27.25	1.088	1.079	1.032	1.043	41.25	1.464	1.461	1.377	1.391
13.50	0.256	0.380	0.272	0.368	27.50	1.095	1.086	1.039	1.050	41.50	1.470	1.466	1.383	1.396
13.75	0.298	0.414	0.309	0.399	27.75	1.102	1.093	1.046	1.057	41.75	1.475	1.472	1.388	1.402
14.00	0.337	0.446	0.343	0.429	28.00	1.109	1.100	1.053	1.064	42.00	1.481	1.478	1.393	1.407
14.25	0.374	0.477	0.376	0.456	28.25	1.116	1.107	1.059	1.070	42.25	1.486	1.483	1.398	1.412
14.50	0.409	0.506	0.407	0.482	28.50	1.124	1.115	1.066	1.077	42.50	1.492	1.489	1.403	1.417
14.75	0.442	0.533	0.436	0.507	28.75	1.131	1.122	1.073	1.084	42.75	1.497	1.495	1.409	1.423
15.00	0.473	0.559	0.463	0.530	29.00	1.138	1.129	1.080	1.091	43.00	1.503	1.500	1.414	1.428
15.25	0.503	0.583	0.489	0.552	29.25	1.145	1.136	1.087	1.098	43.25	1.508	1.505	1.419	1.433
15.50	0.531	0.605	0.514	0.572	29.50	1.152	1.144	1.093	1.105	43.50	1.513	1.511	1.424	1.438
15.75	0.558	0.627	0.537	0.592	29.75	1.160	1.151	1.100	1.111	43.75	1.518	1.516	1.429	1.444
16.00	0.583	0.647	0.559	0.610	30.00	1.167	1.158	1.107	1.118	44.00	1.523	1.521	1.434	1.449
16.25	0.607	0.667	0.579	0.628	30.25	1.174	1.165	1.114	1.125	44.25	1.528	1.527	1.439	1.454
16.50	0.630	0.685	0.599	0.645	30.50	1.181	1.173	1.120	1.132	44.50	1.533	1.532	1.444	1.459
16.75	0.651	0.702	0.617	0.661	30.75	1.188	1.180	1.127	1.138	44.75	1.538	1.537	1.449	1.464
17.00	0.671	0.719	0.635	0.676	31.00	1.196	1.187	1.134	1.145	45.00	1.543	1.542	1.454	1.469
17.25	0.690	0.735	0.651	0.691	31.25	1.203	1.194	1.140	1.152	45.25	1.548	1.547	1.459	1.474
17.50	0.708	0.751	0.667	0.706	31.50	1.210	1.202	1.147	1.158	45.50	1.553	1.552	1.464	1.479
17.75	0.726	0.766	0.682	0.720	31.75	1.217	1.209	1.153	1.165	45.75	1.558	1.557	1.468	1.484
18.00	0.742	0.780	0.697	0.734	32.00	1.224	1.216	1.160	1.172	46.00	1.563	1.562	1.473	1.489
18.25	0.758	0.793	0.711	0.747	32.25	1.231	1.223	1.166	1.178	46.25	1.567	1.567	1.478	1.494
18.50	0.773	0.806	0.724	0.759	32.50	1.239	1.231	1.173	1.185	46.50	1.572	1.572	1.483	1.499
18.75	0.787	0.818	0.737	0.771	32.75	1.246	1.238	1.179	1.191	46.75	1.577	1.576	1.488	1.504
19.00	0.801	0.830	0.750	0.783	33.00	1.253	1.245	1.186	1.197	47.00	1.581	1.581	1.492	1.508
19.25	0.814	0.841	0.762	0.794	33.25	1.260	1.252	1.192	1.204	47.25	1.586	1.586	1.497	1.513
19.50	0.827	0.851	0.774	0.804	33.50	1.267	1.259	1.198	1.210	47.50	1.590	1.590	1.502	1.518
19.75	0.840	0.861	0.786	0.814	33.75	1.274	1.266	1.205	1.217	47.75	1.595	1.595	1.506	1.523
20.00	0.852	0.871	0.797	0.824	34.00	1.281	1.273	1.211	1.223	48.00	1.599	1.599	1.511	1.527
20.25	0.863	0.880	0.808	0.834	34.25	1.288	1.280	1.217	1.229	48.25	1.603	1.603	1.516	1.532
20.50	0.874	0.889	0.819	0.843	34.50	1.294	1.287	1.223	1.235	48.50	1.607	1.608	1.520	1.536
20.75	0.885	0.897	0.829	0.852	34.75	1.301	1.294	1.229	1.241	48.75	1.612	1.612	1.525	1.541
21.00	0.895	0.905	0.839	0.860	35.00	1.308	1.301	1.235	1.248	49.00	1.616	1.616	1.529	1.546
21.25	0.905	0.913	0.848	0.869	35.25	1.315	1.308	1.242	1.254	49.25	1.620	1.620	1.534	1.550
21.50	0.914	0.921	0.858	0.877	35.50	1.322	1.315	1.248	1.260	49.50	1.624	1.624	1.538	1.554
21.75	0.923	0.928	0.867	0.885	35.75	1.328	1.322	1.254	1.266	49.75	1.628	1.628	1.543	1.559
22.00	0.932	0.935	0.876	0.893	36.00	1.335	1.329	1.260	1.272	50.00	1.632	1.632	1.547	1.563

Table S16: Theoretical Assymetric Parameter for $2b_1$ Orbital. Energy in eV.

E	SEP-L	SE-L	SEP-V	SE-V	E	SEP-L	SE-L	SEP-V	SE-V	E	SEP-L	SE-L	SEP-V	SE-V
9.25	-0.224	-0.655	-0.248	-0.664	23.00	1.010	0.992	0.959	0.957	36.75	1.363	1.355	1.300	1.307
9.50	-0.208	-0.664	-0.233	-0.679	23.25	1.018	0.999	0.967	0.964	37.00	1.368	1.361	1.305	1.313
9.75	-0.212	-0.656	-0.241	-0.674	23.50	1.026	1.006	0.976	0.971	37.25	1.374	1.367	1.310	1.318
10.00	-0.236	-0.634	-0.270	-0.653	23.75	1.033	1.013	0.983	0.979	37.50	1.380	1.373	1.315	1.323
10.25	-0.269	-0.603	-0.309	-0.623	24.00	1.040	1.019	0.991	0.986	37.75	1.385	1.379	1.320	1.328
10.50	-0.301	-0.570	-0.344	-0.590	24.25	1.047	1.026	0.998	0.993	38.00	1.391	1.384	1.325	1.334
10.75	-0.326	-0.535	-0.369	-0.553	24.50	1.054	1.033	1.005	1.000	38.25	1.396	1.390	1.331	1.339
11.00	-0.340	-0.489	-0.382	-0.505	24.75	1.061	1.039	1.012	1.006	38.50	1.402	1.396	1.336	1.344
11.25	-0.345	-0.428	-0.382	-0.443	25.00	1.067	1.046	1.018	1.013	38.75	1.407	1.401	1.341	1.349
11.50	-0.340	-0.357	-0.373	-0.372	25.25	1.074	1.052	1.025	1.020	39.00	1.413	1.407	1.346	1.354
11.75	-0.328	-0.284	-0.356	-0.299	25.50	1.080	1.059	1.031	1.027	39.25	1.418	1.412	1.351	1.359
12.00	-0.308	-0.212	-0.331	-0.228	25.75	1.086	1.066	1.038	1.034	39.50	1.423	1.417	1.355	1.364
12.25	-0.279	-0.143	-0.297	-0.159	26.00	1.092	1.072	1.044	1.040	39.75	1.428	1.423	1.360	1.369
12.50	-0.240	-0.076	-0.254	-0.094	26.25	1.099	1.079	1.050	1.047	40.00	1.434	1.428	1.365	1.374
12.75	-0.192	-0.013	-0.202	-0.032	26.50	1.105	1.086	1.057	1.054	40.25	1.439	1.433	1.370	1.379
13.00	-0.137	0.047	-0.146	0.027	26.75	1.111	1.092	1.063	1.061	40.50	1.444	1.439	1.375	1.384
13.25	-0.080	0.104	-0.088	0.082	27.00	1.117	1.099	1.069	1.068	40.75	1.449	1.444	1.380	1.389
13.50	-0.021	0.159	-0.030	0.135	27.25	1.123	1.106	1.075	1.074	41.00	1.454	1.449	1.384	1.394
13.75	0.037	0.210	0.027	0.184	27.50	1.130	1.113	1.082	1.081	41.25	1.459	1.454	1.389	1.398
14.00	0.093	0.259	0.081	0.231	27.75	1.136	1.120	1.088	1.088	41.50	1.464	1.459	1.394	1.403
14.25	0.147	0.304	0.133	0.275	28.00	1.142	1.126	1.094	1.094	41.75	1.469	1.464	1.398	1.408
14.50	0.198	0.348	0.182	0.316	28.25	1.149	1.133	1.100	1.101	42.00	1.473	1.469	1.403	1.413
14.75	0.248	0.389	0.229	0.356	28.50	1.155	1.140	1.106	1.108	42.25	1.478	1.474	1.407	1.417
15.00	0.294	0.428	0.273	0.393	28.75	1.162	1.147	1.113	1.114	42.50	1.483	1.479	1.412	1.422
15.25	0.339	0.464	0.315	0.428	29.00	1.168	1.154	1.119	1.121	42.75	1.488	1.483	1.417	1.427
15.50	0.382	0.499	0.355	0.461	29.25	1.175	1.161	1.125	1.128	43.00	1.492	1.488	1.421	1.431
15.75	0.422	0.532	0.393	0.493	29.50	1.181	1.168	1.131	1.134	43.25	1.497	1.493	1.426	1.436
16.00	0.460	0.563	0.429	0.523	29.75	1.188	1.174	1.138	1.141	43.50	1.501	1.498	1.430	1.440
16.25	0.497	0.593	0.463	0.551	30.00	1.194	1.181	1.144	1.147	43.75	1.506	1.502	1.434	1.445
16.50	0.531	0.621	0.495	0.579	30.25	1.201	1.188	1.150	1.154	44.00	1.510	1.507	1.439	1.449
16.75	0.564	0.648	0.526	0.604	30.50	1.207	1.195	1.156	1.160	44.25	1.515	1.512	1.443	1.454
17.00	0.595	0.673	0.555	0.629	30.75	1.214	1.202	1.162	1.167	44.50	1.519	1.516	1.448	1.458
17.25	0.625	0.696	0.583	0.652	31.00	1.220	1.209	1.168	1.173	44.75	1.523	1.521	1.452	1.463
17.50	0.653	0.719	0.610	0.674	31.25	1.227	1.215	1.174	1.179	45.00	1.528	1.525	1.456	1.467
17.75	0.680	0.740	0.635	0.696	31.50	1.233	1.222	1.181	1.186	45.25	1.532	1.529	1.461	1.472
18.00	0.705	0.760	0.659	0.716	31.75	1.239	1.229	1.187	1.192	45.50	1.536	1.534	1.465	1.476
18.25	0.730	0.779	0.682	0.735	32.00	1.246	1.236	1.193	1.198	45.75	1.540	1.538	1.469	1.480
18.50	0.753	0.797	0.704	0.753	32.25	1.252	1.242	1.199	1.204	46.00	1.545	1.542	1.473	1.485
18.75	0.775	0.814	0.725	0.770	32.50	1.259	1.249	1.204	1.210	46.25	1.549	1.547	1.478	1.489
19.00	0.796	0.830	0.746	0.786	32.75	1.265	1.255	1.210	1.216	46.50	1.553	1.551	1.482	1.494
19.25	0.815	0.845	0.765	0.801	33.00	1.272	1.262	1.216	1.222	46.75	1.557	1.555	1.486	1.498
19.50	0.834	0.859	0.783	0.815	33.25	1.278	1.269	1.222	1.228	47.00	1.561	1.559	1.490	1.502
19.75	0.852	0.872	0.800	0.829	33.50	1.284	1.275	1.228	1.234	47.25	1.565	1.563	1.494	1.506
20.00	0.868	0.884	0.817	0.842	33.75	1.290	1.281	1.234	1.240	47.50	1.569	1.567	1.498	1.511
20.25	0.884	0.896	0.832	0.854	34.00	1.297	1.288	1.239	1.246	47.75	1.573	1.571	1.502	1.515
20.50	0.899	0.907	0.847	0.866	34.25	1.303	1.294	1.245	1.252	48.00	1.576	1.575	1.507	1.519
20.75	0.913	0.917	0.861	0.877	34.50	1.309	1.301	1.251	1.257	48.25	1.580	1.579	1.511	1.523
21.00	0.927	0.927	0.875	0.887	34.75	1.315	1.307	1.256	1.263	48.50	1.584	1.583	1.515	1.527
21.25	0.939	0.937	0.887	0.897	35.00	1.321	1.313	1.262	1.269	48.75	1.588	1.587	1.519	1.531
21.50	0.951	0.946	0.899	0.907	35.25	1.327	1.319	1.267	1.274	49.00	1.591	1.591	1.523	1.535
21.75	0.962	0.954	0.910	0.916	35.50	1.333	1.325	1.273	1.280	49.25	1.595	1.595	1.527	1.540
22.00	0.973	0.962	0.921	0.925	35.75	1.339	1.331	1.278	1.286	49.50	1.599	1.598	1.531	1.544
22.25	0.983	0.970	0.931	0.933	36.00	1.345	1.338	1.284	1.291	49.75	1.602	1.602	1.535	1.548
22.50	0.992	0.978	0.941	0.941	36.25	1.351	1.344	1.289	1.297	50.00	1.606	1.606	1.538	1.552
22.75	1.001	0.985	0.950	0.949	36.50	1.357	1.350	1.294	1.302					

Table S17: Theoretical Assymetric Parameter for $9a_1$ Orbital. Energy in eV.

E	SEP-L	SE-L	SEP-V	SE-V	E	SEP-L	SE-L	SEP-V	SE-V	E	SEP-L	SE-L	SEP-V	SE-V
13.00	0.664	0.721	0.660	0.709	25.50	0.138	0.041	0.099	0.013	38.00	0.461	0.465	0.410	0.418
13.25	0.539	0.521	0.537	0.512	25.75	0.140	0.041	0.101	0.014	38.25	0.469	0.472	0.416	0.424
13.50	0.414	0.330	0.414	0.323	26.00	0.141	0.042	0.101	0.016	38.50	0.476	0.479	0.423	0.429
13.75	0.299	0.173	0.299	0.166	26.25	0.140	0.044	0.100	0.018	38.75	0.484	0.487	0.429	0.435
14.00	0.200	0.058	0.200	0.051	26.50	0.139	0.047	0.099	0.021	39.00	0.491	0.494	0.435	0.441
14.25	0.121	-0.017	0.119	-0.025	26.75	0.138	0.050	0.097	0.024	39.25	0.498	0.501	0.440	0.447
14.50	0.062	-0.057	0.059	-0.064	27.00	0.136	0.054	0.096	0.029	39.50	0.505	0.508	0.446	0.453
14.75	0.022	-0.068	0.019	-0.076	27.25	0.134	0.059	0.094	0.034	39.75	0.513	0.515	0.452	0.458
15.00	0.000	-0.059	-0.005	-0.067	27.50	0.133	0.065	0.093	0.040	40.00	0.520	0.523	0.458	0.464
15.25	-0.008	-0.036	-0.013	-0.044	27.75	0.132	0.071	0.093	0.047	40.25	0.527	0.530	0.463	0.470
15.50	-0.003	-0.005	-0.009	-0.014	28.00	0.132	0.078	0.093	0.055	40.50	0.534	0.537	0.469	0.476
15.75	0.012	0.028	0.006	0.019	28.25	0.133	0.086	0.094	0.063	40.75	0.541	0.544	0.475	0.482
16.00	0.034	0.060	0.027	0.052	28.50	0.134	0.095	0.096	0.072	41.00	0.548	0.552	0.480	0.488
16.25	0.062	0.089	0.054	0.080	28.75	0.136	0.104	0.099	0.082	41.25	0.555	0.559	0.486	0.494
16.50	0.093	0.113	0.084	0.104	29.00	0.139	0.113	0.102	0.092	41.50	0.563	0.566	0.492	0.500
16.75	0.125	0.131	0.116	0.121	29.25	0.143	0.124	0.106	0.102	41.75	0.570	0.574	0.497	0.506
17.00	0.157	0.142	0.147	0.132	29.50	0.147	0.134	0.111	0.113	42.00	0.577	0.581	0.503	0.512
17.25	0.188	0.146	0.176	0.135	29.75	0.152	0.145	0.117	0.124	42.25	0.584	0.588	0.509	0.519
17.50	0.215	0.143	0.202	0.133	30.00	0.158	0.156	0.123	0.135	42.50	0.591	0.595	0.515	0.525
17.75	0.239	0.135	0.225	0.124	30.25	0.165	0.168	0.131	0.147	42.75	0.598	0.603	0.521	0.531
18.00	0.258	0.121	0.242	0.110	30.50	0.172	0.179	0.138	0.158	43.00	0.605	0.610	0.527	0.537
18.25	0.271	0.103	0.255	0.091	30.75	0.180	0.191	0.147	0.170	43.25	0.613	0.617	0.533	0.543
18.50	0.280	0.083	0.262	0.071	31.00	0.189	0.203	0.156	0.182	43.50	0.620	0.624	0.539	0.549
18.75	0.283	0.062	0.264	0.049	31.25	0.198	0.215	0.165	0.193	43.75	0.627	0.631	0.545	0.555
19.00	0.281	0.040	0.260	0.027	31.50	0.207	0.226	0.174	0.205	44.00	0.634	0.638	0.550	0.561
19.25	0.274	0.019	0.252	0.005	31.75	0.217	0.238	0.184	0.216	44.25	0.641	0.645	0.556	0.567
19.50	0.263	-0.001	0.240	-0.015	32.00	0.227	0.250	0.195	0.227	44.50	0.648	0.651	0.562	0.573
19.75	0.249	-0.018	0.224	-0.033	32.25	0.237	0.261	0.205	0.238	44.75	0.655	0.658	0.568	0.578
20.00	0.232	-0.031	0.206	-0.047	32.50	0.248	0.272	0.216	0.249	45.00	0.662	0.665	0.574	0.584
20.25	0.213	-0.042	0.186	-0.059	32.75	0.259	0.283	0.226	0.259	45.25	0.669	0.671	0.580	0.590
20.50	0.193	-0.049	0.166	-0.067	33.00	0.270	0.294	0.237	0.270	45.50	0.675	0.677	0.586	0.595
20.75	0.174	-0.052	0.146	-0.071	33.25	0.280	0.305	0.248	0.279	45.75	0.682	0.684	0.591	0.600
21.00	0.154	-0.051	0.126	-0.072	33.50	0.291	0.315	0.258	0.289	46.00	0.688	0.690	0.597	0.606
21.25	0.137	-0.048	0.107	-0.069	33.75	0.302	0.325	0.268	0.298	46.25	0.695	0.696	0.602	0.611
21.50	0.121	-0.041	0.091	-0.063	34.00	0.313	0.335	0.279	0.307	46.50	0.701	0.702	0.608	0.616
21.75	0.108	-0.032	0.076	-0.055	34.25	0.324	0.345	0.289	0.316	46.75	0.707	0.707	0.613	0.621
22.00	0.097	-0.022	0.065	-0.046	34.50	0.334	0.354	0.299	0.324	47.00	0.714	0.713	0.619	0.626
22.25	0.089	-0.011	0.057	-0.035	34.75	0.345	0.363	0.308	0.332	47.25	0.720	0.719	0.624	0.631
22.50	0.084	0.000	0.051	-0.025	35.00	0.355	0.372	0.318	0.340	47.50	0.726	0.724	0.629	0.635
22.75	0.082	0.011	0.049	-0.015	35.25	0.365	0.381	0.327	0.348	47.75	0.731	0.729	0.634	0.640
23.00	0.082	0.020	0.049	-0.007	35.50	0.375	0.389	0.336	0.355	48.00	0.737	0.734	0.639	0.644
23.25	0.085	0.028	0.051	0.000	35.75	0.384	0.397	0.344	0.362	48.25	0.743	0.739	0.644	0.649
23.50	0.090	0.033	0.056	0.006	36.00	0.393	0.405	0.353	0.369	48.50	0.748	0.744	0.649	0.653
23.75	0.096	0.037	0.062	0.010	36.25	0.403	0.413	0.361	0.375	48.75	0.753	0.749	0.653	0.657
24.00	0.104	0.040	0.068	0.012	36.50	0.411	0.421	0.368	0.382	49.00	0.758	0.753	0.658	0.661
24.25	0.111	0.041	0.075	0.013	36.75	0.420	0.428	0.376	0.388	49.25	0.764	0.758	0.662	0.665
24.50	0.119	0.042	0.082	0.014	37.00	0.429	0.436	0.383	0.394	49.50	0.768	0.762	0.666	0.669
24.75	0.126	0.042	0.088	0.014	37.25	0.437	0.443	0.390	0.400	49.75	0.773	0.766	0.671	0.672
25.00	0.131	0.041	0.093	0.013	37.50	0.445	0.451	0.397	0.406	50.00	0.778	0.771	0.675	0.676
25.25	0.136	0.041	0.097	0.013	37.75	0.453	0.458	0.404	0.412					

Table S18: Theoretical Assymetric Parameter for $6b_2$ Orbital. Energy in eV.

E	SEP-L	SE-L	SEP-V	SE-V	E	SEP-L	SE-L	SEP-V	SE-V	E	SEP-L	SE-L	SEP-V	SE-V
13.00	0.589	0.604	0.604	0.592	25.50	0.487	0.354	0.471	0.343	38.00	0.606	0.614	0.553	0.559
13.25	0.633	0.574	0.647	0.571	25.75	0.481	0.348	0.464	0.336	38.25	0.614	0.622	0.559	0.565
13.50	0.625	0.487	0.645	0.493	26.00	0.474	0.343	0.456	0.330	38.50	0.623	0.630	0.566	0.572
13.75	0.595	0.387	0.620	0.402	26.25	0.467	0.338	0.447	0.325	38.75	0.631	0.638	0.572	0.578
14.00	0.553	0.296	0.582	0.317	26.50	0.459	0.334	0.438	0.320	39.00	0.639	0.646	0.579	0.584
14.25	0.506	0.228	0.539	0.254	26.75	0.451	0.331	0.428	0.316	39.25	0.647	0.654	0.585	0.590
14.50	0.460	0.190	0.495	0.218	27.00	0.443	0.329	0.420	0.313	39.50	0.655	0.661	0.591	0.596
14.75	0.418	0.178	0.455	0.205	27.25	0.436	0.327	0.411	0.311	39.75	0.663	0.669	0.597	0.602
15.00	0.384	0.186	0.422	0.211	27.50	0.429	0.326	0.404	0.309	40.00	0.671	0.677	0.603	0.609
15.25	0.359	0.206	0.398	0.227	27.75	0.423	0.326	0.397	0.308	40.25	0.679	0.685	0.609	0.615
15.50	0.346	0.234	0.383	0.251	28.00	0.418	0.326	0.390	0.308	40.50	0.687	0.693	0.615	0.621
15.75	0.343	0.264	0.377	0.277	28.25	0.413	0.327	0.385	0.308	40.75	0.695	0.701	0.622	0.627
16.00	0.349	0.293	0.379	0.303	28.50	0.409	0.328	0.380	0.309	41.00	0.703	0.708	0.628	0.634
16.25	0.361	0.318	0.387	0.326	28.75	0.405	0.330	0.376	0.311	41.25	0.711	0.716	0.634	0.640
16.50	0.377	0.338	0.398	0.344	29.00	0.402	0.333	0.373	0.313	41.50	0.718	0.724	0.640	0.646
16.75	0.393	0.352	0.410	0.356	29.25	0.400	0.336	0.370	0.316	41.75	0.726	0.732	0.646	0.653
17.00	0.409	0.359	0.422	0.363	29.50	0.399	0.339	0.368	0.319	42.00	0.734	0.739	0.652	0.659
17.25	0.424	0.361	0.432	0.364	29.75	0.398	0.344	0.367	0.323	42.25	0.742	0.747	0.658	0.666
17.50	0.436	0.357	0.441	0.361	30.00	0.398	0.348	0.367	0.328	42.50	0.749	0.755	0.664	0.672
17.75	0.446	0.350	0.448	0.354	30.25	0.398	0.354	0.367	0.333	42.75	0.757	0.762	0.670	0.679
18.00	0.453	0.341	0.453	0.344	30.50	0.399	0.360	0.368	0.338	43.00	0.765	0.770	0.677	0.685
18.25	0.457	0.329	0.454	0.333	30.75	0.401	0.366	0.369	0.344	43.25	0.772	0.777	0.683	0.691
18.50	0.457	0.318	0.454	0.322	31.00	0.403	0.372	0.371	0.350	43.50	0.780	0.784	0.689	0.698
18.75	0.455	0.308	0.450	0.311	31.25	0.405	0.379	0.373	0.357	43.75	0.787	0.792	0.695	0.704
19.00	0.450	0.298	0.445	0.301	31.50	0.409	0.387	0.377	0.364	44.00	0.795	0.799	0.702	0.710
19.25	0.444	0.291	0.438	0.294	31.75	0.412	0.395	0.380	0.372	44.25	0.802	0.806	0.708	0.716
19.50	0.436	0.286	0.429	0.289	32.00	0.417	0.403	0.384	0.379	44.50	0.809	0.813	0.714	0.723
19.75	0.427	0.283	0.420	0.286	32.25	0.422	0.411	0.389	0.387	44.75	0.817	0.820	0.720	0.729
20.00	0.418	0.284	0.411	0.287	32.50	0.427	0.420	0.394	0.395	45.00	0.824	0.826	0.726	0.734
20.25	0.409	0.287	0.402	0.290	32.75	0.433	0.429	0.400	0.403	45.25	0.831	0.833	0.733	0.740
20.50	0.402	0.293	0.394	0.296	33.00	0.439	0.438	0.406	0.411	45.50	0.838	0.840	0.739	0.746
20.75	0.395	0.301	0.388	0.305	33.25	0.446	0.447	0.412	0.420	45.75	0.845	0.846	0.745	0.752
21.00	0.391	0.313	0.383	0.316	33.50	0.453	0.456	0.419	0.428	46.00	0.851	0.852	0.751	0.757
21.25	0.388	0.327	0.380	0.330	33.75	0.460	0.465	0.426	0.436	46.25	0.858	0.859	0.756	0.763
21.50	0.387	0.342	0.379	0.346	34.00	0.468	0.475	0.433	0.445	46.50	0.865	0.865	0.762	0.768
21.75	0.388	0.359	0.380	0.362	34.25	0.475	0.484	0.440	0.453	46.75	0.871	0.871	0.768	0.774
22.00	0.391	0.374	0.383	0.378	34.50	0.484	0.493	0.448	0.461	47.00	0.877	0.876	0.773	0.779
22.25	0.396	0.388	0.388	0.391	34.75	0.492	0.502	0.455	0.469	47.25	0.883	0.882	0.779	0.784
22.50	0.403	0.399	0.396	0.402	35.00	0.501	0.511	0.463	0.477	47.50	0.890	0.888	0.784	0.789
22.75	0.412	0.407	0.405	0.409	35.25	0.509	0.521	0.471	0.484	47.75	0.895	0.893	0.790	0.794
23.00	0.423	0.411	0.416	0.412	35.50	0.518	0.530	0.479	0.492	48.00	0.901	0.898	0.795	0.798
23.25	0.436	0.412	0.428	0.411	35.75	0.527	0.538	0.487	0.499	48.25	0.907	0.904	0.800	0.803
23.50	0.448	0.409	0.441	0.408	36.00	0.536	0.547	0.494	0.506	48.50	0.913	0.909	0.805	0.807
23.75	0.460	0.404	0.453	0.402	36.25	0.545	0.556	0.502	0.513	48.75	0.918	0.914	0.810	0.812
24.00	0.472	0.398	0.463	0.394	36.50	0.554	0.564	0.510	0.520	49.00	0.923	0.919	0.814	0.816
24.25	0.481	0.391	0.472	0.386	36.75	0.563	0.573	0.517	0.527	49.25	0.929	0.923	0.819	0.820
24.50	0.487	0.383	0.477	0.377	37.00	0.571	0.581	0.524	0.534	49.50	0.934	0.928	0.824	0.824
24.75	0.491	0.375	0.480	0.368	37.25	0.580	0.589	0.532	0.540	49.75	0.939	0.932	0.828	0.828
25.00	0.492	0.368	0.480	0.359	37.50	0.589	0.598	0.539	0.547	50.00	0.943	0.937	0.832	0.832
25.25	0.491	0.361	0.476	0.351	37.75	0.597	0.606	0.546	0.553					

Table S19: Theoretical Assymetric Parameter for $1b_1$ Orbital. Energy in eV.

E	SEP-L	SE-L	SEP-V	SE-V	E	SEP-L	SE-L	SEP-V	SE-V	E	SEP-L	SE-L	SEP-V	SE-V
13.75	0.375	0.436	0.363	0.425	26.00	1.245	1.254	1.179	1.192	38.25	1.551	1.540	1.471	1.459
14.00	0.478	0.544	0.466	0.535	26.25	1.257	1.265	1.191	1.203	38.50	1.553	1.542	1.472	1.460
14.25	0.560	0.614	0.548	0.606	26.50	1.269	1.276	1.203	1.214	38.75	1.554	1.543	1.473	1.461
14.50	0.629	0.660	0.617	0.652	26.75	1.281	1.287	1.214	1.225	39.00	1.556	1.545	1.473	1.462
14.75	0.688	0.694	0.675	0.685	27.00	1.292	1.298	1.225	1.235	39.25	1.557	1.547	1.474	1.463
15.00	0.738	0.730	0.724	0.720	27.25	1.302	1.308	1.236	1.246	39.50	1.559	1.549	1.475	1.464
15.25	0.779	0.773	0.765	0.762	27.50	1.313	1.318	1.247	1.256	39.75	1.560	1.550	1.475	1.465
15.50	0.813	0.817	0.799	0.805	27.75	1.323	1.328	1.257	1.265	40.00	1.562	1.552	1.476	1.467
15.75	0.842	0.857	0.825	0.844	28.00	1.333	1.337	1.266	1.274	40.25	1.563	1.554	1.476	1.468
16.00	0.866	0.890	0.848	0.876	28.25	1.342	1.346	1.276	1.283	40.50	1.565	1.555	1.477	1.469
16.25	0.888	0.915	0.869	0.900	28.50	1.352	1.355	1.285	1.292	40.75	1.566	1.557	1.478	1.470
16.50	0.912	0.932	0.891	0.915	28.75	1.361	1.364	1.294	1.301	41.00	1.567	1.559	1.478	1.471
16.75	0.938	0.941	0.915	0.921	29.00	1.370	1.372	1.303	1.309	41.25	1.569	1.561	1.479	1.473
17.00	0.963	0.943	0.938	0.919	29.25	1.378	1.380	1.312	1.317	41.50	1.570	1.563	1.480	1.474
17.25	0.986	0.938	0.958	0.910	29.50	1.386	1.388	1.320	1.325	41.75	1.572	1.564	1.480	1.476
17.50	1.004	0.927	0.975	0.895	29.75	1.394	1.396	1.328	1.333	42.00	1.573	1.566	1.481	1.477
17.75	1.017	0.913	0.985	0.877	30.00	1.402	1.403	1.336	1.340	42.25	1.574	1.568	1.482	1.479
18.00	1.025	0.898	0.989	0.858	30.25	1.410	1.410	1.343	1.347	42.50	1.576	1.570	1.483	1.480
18.25	1.027	0.884	0.988	0.840	30.50	1.417	1.417	1.350	1.354	42.75	1.577	1.572	1.484	1.482
18.50	1.024	0.872	0.981	0.826	30.75	1.424	1.424	1.357	1.360	43.00	1.579	1.574	1.485	1.484
18.75	1.017	0.864	0.970	0.816	31.00	1.431	1.431	1.364	1.367	43.25	1.580	1.576	1.486	1.485
19.00	1.007	0.861	0.957	0.811	31.25	1.438	1.437	1.371	1.373	43.50	1.582	1.578	1.487	1.487
19.25	0.996	0.862	0.943	0.810	31.50	1.444	1.443	1.377	1.379	43.75	1.584	1.580	1.489	1.489
19.50	0.985	0.867	0.929	0.814	31.75	1.451	1.449	1.383	1.385	44.00	1.585	1.581	1.490	1.491
19.75	0.976	0.875	0.918	0.821	32.00	1.457	1.455	1.389	1.390	44.25	1.587	1.583	1.491	1.492
20.00	0.970	0.886	0.910	0.832	32.25	1.463	1.460	1.395	1.395	44.50	1.589	1.585	1.493	1.494
20.25	0.966	0.899	0.904	0.844	32.50	1.468	1.466	1.401	1.400	44.75	1.590	1.587	1.494	1.496
20.50	0.966	0.914	0.903	0.859	32.75	1.474	1.471	1.406	1.405	45.00	1.592	1.589	1.496	1.498
20.75	0.969	0.930	0.905	0.874	33.00	1.479	1.476	1.411	1.410	45.25	1.594	1.591	1.497	1.500
21.00	0.974	0.947	0.910	0.891	33.25	1.484	1.480	1.416	1.414	45.50	1.595	1.593	1.499	1.501
21.25	0.982	0.965	0.917	0.908	33.50	1.489	1.485	1.421	1.418	45.75	1.597	1.594	1.500	1.503
21.50	0.992	0.982	0.927	0.925	33.75	1.494	1.489	1.425	1.422	46.00	1.599	1.596	1.502	1.505
21.75	1.003	1.000	0.938	0.943	34.00	1.499	1.493	1.429	1.425	46.25	1.600	1.598	1.504	1.507
22.00	1.016	1.018	0.951	0.960	34.25	1.503	1.497	1.433	1.429	46.50	1.602	1.600	1.505	1.508
22.25	1.030	1.035	0.965	0.977	34.50	1.507	1.501	1.437	1.432	46.75	1.604	1.602	1.507	1.510
22.50	1.045	1.052	0.979	0.994	34.75	1.511	1.505	1.441	1.435	47.00	1.605	1.603	1.508	1.512
22.75	1.060	1.069	0.994	1.011	35.00	1.515	1.508	1.444	1.438	47.25	1.607	1.605	1.510	1.514
23.00	1.075	1.086	1.009	1.027	35.25	1.519	1.511	1.447	1.440	47.50	1.608	1.607	1.512	1.515
23.25	1.090	1.102	1.025	1.043	35.50	1.522	1.514	1.450	1.443	47.75	1.610	1.608	1.513	1.517
23.50	1.106	1.118	1.040	1.058	35.75	1.526	1.517	1.453	1.445	48.00	1.612	1.610	1.515	1.519
23.75	1.121	1.133	1.055	1.074	36.00	1.529	1.520	1.456	1.447	48.25	1.613	1.612	1.517	1.520
24.00	1.136	1.148	1.070	1.088	36.25	1.532	1.523	1.458	1.449	48.50	1.615	1.613	1.518	1.522
24.25	1.151	1.163	1.085	1.103	36.50	1.535	1.525	1.460	1.451	48.75	1.616	1.615	1.520	1.523
24.50	1.165	1.177	1.099	1.116	36.75	1.537	1.528	1.462	1.452	49.00	1.618	1.616	1.521	1.525
24.75	1.179	1.191	1.114	1.130	37.00	1.540	1.530	1.464	1.454	49.25	1.619	1.618	1.523	1.527
25.00	1.193	1.204	1.127	1.143	37.25	1.542	1.532	1.466	1.455	49.50	1.621	1.619	1.524	1.528
25.25	1.207	1.217	1.141	1.156	37.50	1.545	1.534	1.467	1.456	49.75	1.622	1.621	1.526	1.530
25.50	1.220	1.230	1.154	1.168	37.75	1.547	1.536	1.469	1.457	50.00	1.624	1.622	1.527	1.531
25.75	1.233	1.242	1.167	1.180	38.00	1.549	1.538	1.470	1.458					

Table S20: Theoretical Assymetric Parameter for $5b_2$ Orbital. Energy in eV.

E	SEP-L	SE-L	SEP-V	SE-V	E	SEP-L	SE-L	SEP-V	SE-V	E	SEP-L	SE-L	SEP-V	SE-V
14.50	0.861	0.963	0.855	0.948	26.50	0.403	0.326	0.380	0.305	38.50	0.487	0.485	0.428	0.426
14.75	0.898	0.950	0.895	0.938	26.75	0.402	0.321	0.378	0.299	38.75	0.493	0.490	0.432	0.430
15.00	0.890	0.873	0.888	0.864	27.00	0.400	0.316	0.374	0.294	39.00	0.499	0.496	0.436	0.434
15.25	0.857	0.771	0.857	0.762	27.25	0.398	0.312	0.370	0.289	39.25	0.505	0.501	0.440	0.438
15.50	0.809	0.662	0.809	0.652	27.50	0.394	0.309	0.365	0.285	39.50	0.510	0.507	0.445	0.442
15.75	0.752	0.562	0.752	0.552	27.75	0.390	0.306	0.360	0.282	39.75	0.516	0.513	0.449	0.446
16.00	0.692	0.486	0.691	0.474	28.00	0.385	0.304	0.354	0.279	40.00	0.522	0.518	0.453	0.450
16.25	0.632	0.438	0.631	0.423	28.25	0.380	0.303	0.347	0.277	40.25	0.527	0.524	0.457	0.455
16.50	0.576	0.419	0.574	0.402	28.50	0.375	0.303	0.341	0.276	40.50	0.533	0.530	0.461	0.459
16.75	0.528	0.424	0.525	0.404	28.75	0.370	0.303	0.336	0.276	40.75	0.539	0.536	0.465	0.463
17.00	0.489	0.444	0.484	0.422	29.00	0.365	0.303	0.330	0.276	41.00	0.545	0.542	0.469	0.468
17.25	0.460	0.472	0.455	0.449	29.25	0.361	0.304	0.326	0.277	41.25	0.550	0.548	0.473	0.472
17.50	0.444	0.501	0.437	0.477	29.50	0.358	0.306	0.322	0.279	41.50	0.556	0.554	0.478	0.477
17.75	0.439	0.527	0.430	0.503	29.75	0.355	0.308	0.319	0.280	41.75	0.562	0.560	0.482	0.482
18.00	0.445	0.547	0.434	0.523	30.00	0.353	0.311	0.316	0.283	42.00	0.568	0.567	0.486	0.487
18.25	0.459	0.558	0.446	0.535	30.25	0.352	0.314	0.315	0.285	42.25	0.574	0.573	0.491	0.492
18.50	0.479	0.562	0.464	0.540	30.50	0.351	0.317	0.314	0.288	42.50	0.580	0.580	0.495	0.497
18.75	0.502	0.558	0.485	0.538	30.75	0.351	0.320	0.313	0.291	42.75	0.586	0.586	0.500	0.503
19.00	0.523	0.548	0.506	0.529	31.00	0.352	0.324	0.314	0.295	43.00	0.592	0.593	0.504	0.508
19.25	0.542	0.533	0.524	0.516	31.25	0.353	0.328	0.314	0.298	43.25	0.598	0.600	0.509	0.514
19.50	0.556	0.516	0.537	0.500	31.50	0.354	0.332	0.316	0.302	43.50	0.605	0.607	0.514	0.520
19.75	0.565	0.496	0.546	0.482	31.75	0.356	0.336	0.318	0.306	43.75	0.611	0.614	0.519	0.525
20.00	0.569	0.477	0.550	0.463	32.00	0.359	0.341	0.320	0.310	44.00	0.618	0.621	0.524	0.531
20.25	0.567	0.457	0.549	0.445	32.25	0.362	0.346	0.323	0.314	44.25	0.624	0.628	0.530	0.537
20.50	0.562	0.438	0.543	0.427	32.50	0.365	0.350	0.325	0.318	44.50	0.631	0.635	0.535	0.543
20.75	0.553	0.421	0.534	0.410	32.75	0.368	0.355	0.329	0.323	44.75	0.638	0.642	0.541	0.549
21.00	0.542	0.405	0.523	0.395	33.00	0.372	0.361	0.332	0.327	45.00	0.645	0.649	0.546	0.555
21.25	0.529	0.391	0.511	0.381	33.25	0.376	0.366	0.336	0.332	45.25	0.652	0.656	0.552	0.561
21.50	0.515	0.378	0.497	0.369	33.50	0.380	0.371	0.339	0.336	45.50	0.659	0.663	0.557	0.567
21.75	0.501	0.367	0.483	0.358	33.75	0.384	0.377	0.343	0.341	45.75	0.666	0.670	0.563	0.573
22.00	0.486	0.358	0.469	0.349	34.00	0.389	0.382	0.347	0.346	46.00	0.673	0.677	0.569	0.579
22.25	0.473	0.351	0.456	0.342	34.25	0.393	0.388	0.351	0.350	46.25	0.680	0.684	0.575	0.585
22.50	0.460	0.346	0.443	0.337	34.50	0.398	0.393	0.356	0.355	46.50	0.687	0.691	0.581	0.591
22.75	0.448	0.343	0.431	0.334	34.75	0.403	0.399	0.360	0.360	46.75	0.694	0.698	0.587	0.597
23.00	0.437	0.341	0.420	0.332	35.00	0.408	0.405	0.364	0.365	47.00	0.701	0.705	0.593	0.603
23.25	0.427	0.341	0.410	0.332	35.25	0.414	0.411	0.369	0.369	47.25	0.707	0.711	0.599	0.609
23.50	0.418	0.342	0.401	0.333	35.50	0.419	0.417	0.373	0.374	47.50	0.714	0.718	0.604	0.615
23.75	0.411	0.344	0.394	0.334	35.75	0.424	0.422	0.378	0.379	47.75	0.721	0.725	0.610	0.620
24.00	0.405	0.346	0.388	0.336	36.00	0.430	0.428	0.382	0.383	48.00	0.728	0.731	0.616	0.626
24.25	0.401	0.348	0.384	0.337	36.25	0.436	0.434	0.387	0.388	48.25	0.735	0.737	0.622	0.632
24.50	0.398	0.350	0.381	0.337	36.50	0.441	0.440	0.392	0.392	48.50	0.741	0.744	0.628	0.637
24.75	0.397	0.350	0.379	0.337	36.75	0.447	0.445	0.396	0.397	48.75	0.748	0.750	0.633	0.642
25.00	0.397	0.349	0.378	0.335	37.00	0.453	0.451	0.401	0.401	49.00	0.754	0.756	0.639	0.648
25.25	0.397	0.347	0.379	0.332	37.25	0.458	0.457	0.405	0.405	49.25	0.761	0.762	0.645	0.653
25.50	0.399	0.344	0.379	0.327	37.50	0.464	0.462	0.410	0.409	49.50	0.767	0.768	0.650	0.658
25.75	0.400	0.340	0.380	0.322	37.75	0.470	0.468	0.414	0.414	49.75	0.773	0.773	0.656	0.663
26.00	0.402	0.336	0.381	0.317	38.00	0.476	0.474	0.419	0.418	50.00	0.779	0.779	0.661	0.668
26.25	0.403	0.331	0.380	0.311	38.25	0.482	0.479	0.423	0.422					

Table S21: Theoretical Assymetric Parameter for $8a_1$ Orbital. Energy in eV.

E	SEP-L	SE-L	SEP-V	SE-V	E	SEP-L	SE-L	SEP-V	SE-V	E	SEP-L	SE-L	SEP-V	SE-V
15.00	0.637	0.705	0.644	0.708	26.75	0.441	0.316	0.418	0.292	38.50	0.464	0.455	0.402	0.392
15.25	0.756	0.791	0.767	0.797	27.00	0.433	0.311	0.408	0.286	38.75	0.470	0.461	0.406	0.396
15.50	0.823	0.790	0.837	0.794	27.25	0.425	0.306	0.398	0.280	39.00	0.475	0.466	0.409	0.399
15.75	0.856	0.725	0.871	0.727	27.50	0.417	0.303	0.387	0.276	39.25	0.480	0.472	0.413	0.403
16.00	0.866	0.651	0.882	0.652	27.75	0.409	0.300	0.377	0.272	39.50	0.486	0.477	0.416	0.407
16.25	0.855	0.641	0.871	0.642	28.00	0.401	0.297	0.368	0.269	39.75	0.491	0.483	0.420	0.410
16.50	0.827	0.683	0.842	0.686	28.25	0.394	0.295	0.360	0.267	40.00	0.497	0.489	0.423	0.414
16.75	0.785	0.726	0.798	0.731	28.50	0.388	0.294	0.352	0.265	40.25	0.502	0.495	0.427	0.418
17.00	0.736	0.749	0.747	0.757	28.75	0.382	0.293	0.345	0.264	40.50	0.508	0.501	0.430	0.423
17.25	0.693	0.754	0.702	0.765	29.00	0.376	0.293	0.339	0.263	40.75	0.513	0.507	0.434	0.427
17.50	0.669	0.751	0.677	0.764	29.25	0.372	0.293	0.333	0.263	41.00	0.519	0.513	0.438	0.432
17.75	0.672	0.744	0.678	0.757	29.50	0.368	0.294	0.328	0.264	41.25	0.525	0.520	0.441	0.437
18.00	0.692	0.733	0.699	0.747	29.75	0.364	0.295	0.324	0.265	41.50	0.531	0.527	0.445	0.442
18.25	0.718	0.718	0.725	0.733	30.00	0.362	0.296	0.321	0.266	41.75	0.537	0.533	0.449	0.447
18.50	0.739	0.700	0.747	0.715	30.25	0.359	0.298	0.319	0.268	42.00	0.543	0.540	0.454	0.452
18.75	0.750	0.677	0.759	0.692	30.50	0.358	0.300	0.317	0.270	42.25	0.550	0.548	0.458	0.458
19.00	0.753	0.652	0.763	0.666	30.75	0.356	0.303	0.315	0.272	42.50	0.556	0.555	0.463	0.464
19.25	0.748	0.624	0.759	0.638	31.00	0.355	0.305	0.314	0.275	42.75	0.563	0.562	0.467	0.470
19.50	0.739	0.595	0.750	0.609	31.25	0.355	0.309	0.314	0.278	43.00	0.569	0.570	0.472	0.476
19.75	0.725	0.567	0.736	0.579	31.50	0.355	0.312	0.314	0.281	43.25	0.576	0.577	0.478	0.482
20.00	0.707	0.539	0.719	0.552	31.75	0.356	0.316	0.315	0.285	43.50	0.583	0.585	0.483	0.489
20.25	0.688	0.515	0.699	0.526	32.00	0.356	0.320	0.316	0.289	43.75	0.590	0.592	0.488	0.495
20.50	0.667	0.493	0.677	0.503	32.25	0.358	0.324	0.317	0.293	44.00	0.598	0.600	0.494	0.502
20.75	0.645	0.473	0.655	0.483	32.50	0.359	0.328	0.319	0.297	44.25	0.605	0.608	0.500	0.509
21.00	0.624	0.458	0.633	0.466	32.75	0.361	0.333	0.321	0.301	44.50	0.612	0.616	0.506	0.515
21.25	0.603	0.445	0.611	0.453	33.00	0.364	0.338	0.323	0.305	44.75	0.620	0.623	0.512	0.522
21.50	0.583	0.435	0.590	0.442	33.25	0.366	0.343	0.326	0.310	45.00	0.628	0.631	0.518	0.529
21.75	0.565	0.427	0.571	0.433	33.50	0.369	0.348	0.329	0.314	45.25	0.635	0.639	0.525	0.536
22.00	0.549	0.422	0.554	0.427	33.75	0.373	0.353	0.332	0.319	45.50	0.643	0.646	0.531	0.543
22.25	0.535	0.417	0.540	0.421	34.00	0.376	0.359	0.335	0.323	45.75	0.651	0.654	0.538	0.550
22.50	0.524	0.414	0.527	0.417	34.25	0.380	0.364	0.338	0.327	46.00	0.658	0.662	0.545	0.557
22.75	0.514	0.412	0.516	0.414	34.50	0.384	0.369	0.342	0.332	46.25	0.666	0.669	0.551	0.563
23.00	0.506	0.410	0.507	0.411	34.75	0.388	0.375	0.346	0.336	46.50	0.674	0.676	0.558	0.570
23.25	0.500	0.407	0.500	0.407	35.00	0.393	0.380	0.349	0.340	46.75	0.681	0.684	0.565	0.577
23.50	0.495	0.404	0.494	0.403	35.25	0.397	0.386	0.353	0.345	47.00	0.689	0.691	0.572	0.583
23.75	0.491	0.401	0.489	0.398	35.50	0.402	0.391	0.357	0.349	47.25	0.696	0.698	0.578	0.590
24.00	0.488	0.396	0.485	0.391	35.75	0.407	0.397	0.361	0.353	47.50	0.704	0.705	0.585	0.597
24.25	0.485	0.390	0.481	0.384	36.00	0.412	0.402	0.365	0.357	47.75	0.711	0.712	0.592	0.603
24.50	0.483	0.383	0.478	0.375	36.25	0.417	0.408	0.369	0.360	48.00	0.718	0.719	0.598	0.609
24.75	0.481	0.376	0.474	0.365	36.50	0.422	0.413	0.373	0.364	48.25	0.726	0.726	0.605	0.615
25.00	0.478	0.367	0.470	0.355	36.75	0.427	0.418	0.377	0.368	48.50	0.733	0.732	0.611	0.621
25.25	0.475	0.359	0.465	0.345	37.00	0.433	0.423	0.381	0.371	48.75	0.740	0.739	0.618	0.627
25.50	0.472	0.351	0.460	0.335	37.25	0.438	0.429	0.384	0.375	49.00	0.747	0.745	0.624	0.633
25.75	0.467	0.343	0.453	0.325	37.50	0.443	0.434	0.388	0.378	49.25	0.753	0.751	0.630	0.639
26.00	0.462	0.335	0.446	0.315	37.75	0.449	0.439	0.392	0.382	49.50	0.760	0.757	0.637	0.645
26.25	0.456	0.328	0.437	0.307	38.00	0.454	0.445	0.395	0.385	49.75	0.766	0.763	0.643	0.650
26.50	0.449	0.321	0.428	0.299	38.25	0.459	0.450	0.399	0.389	50.00	0.773	0.769	0.649	0.656

Table S22: Theoretical Assymetric Parameter for $7a_1$ Orbital. Energy in eV.

E	SEP-L	SE-L	SEP-V	SE-V	E	SEP-L	SE-L	SEP-V	SE-V	E	SEP-L	SE-L	SEP-V	SE-V
17.50	1.126	1.013	1.131	1.021	28.50	1.076	1.126	1.027	1.074	39.50	1.063	1.066	1.066	1.068
17.75	1.161	1.051	1.173	1.066	28.75	1.080	1.128	1.031	1.077	39.75	1.063	1.066	1.066	1.068
18.00	1.175	1.060	1.190	1.075	29.00	1.084	1.130	1.035	1.081	40.00	1.062	1.066	1.065	1.069
18.25	1.181	1.047	1.197	1.059	29.25	1.087	1.132	1.039	1.084	40.25	1.061	1.067	1.065	1.069
18.50	1.182	1.011	1.197	1.017	29.50	1.090	1.133	1.043	1.087	40.50	1.061	1.067	1.064	1.069
18.75	1.177	0.951	1.190	0.949	29.75	1.093	1.134	1.047	1.090	40.75	1.061	1.068	1.064	1.070
19.00	1.166	0.872	1.176	0.865	30.00	1.096	1.134	1.051	1.093	41.00	1.061	1.069	1.064	1.071
19.25	1.148	0.793	1.154	0.783	30.25	1.099	1.135	1.055	1.095	41.25	1.061	1.070	1.064	1.072
19.50	1.121	0.740	1.123	0.728	30.50	1.101	1.134	1.059	1.097	41.50	1.062	1.071	1.065	1.072
19.75	1.085	0.721	1.084	0.709	30.75	1.103	1.134	1.063	1.099	41.75	1.062	1.072	1.065	1.073
20.00	1.041	0.730	1.036	0.718	31.00	1.105	1.133	1.067	1.100	42.00	1.063	1.073	1.066	1.075
20.25	0.989	0.752	0.982	0.740	31.25	1.107	1.132	1.070	1.101	42.25	1.064	1.075	1.066	1.076
20.50	0.934	0.778	0.925	0.766	31.50	1.108	1.131	1.073	1.102	42.50	1.065	1.076	1.067	1.077
20.75	0.882	0.805	0.871	0.791	31.75	1.109	1.129	1.076	1.102	42.75	1.066	1.078	1.068	1.078
21.00	0.837	0.829	0.826	0.814	32.00	1.110	1.127	1.078	1.102	43.00	1.068	1.080	1.069	1.079
21.25	0.806	0.852	0.794	0.835	32.25	1.110	1.126	1.081	1.102	43.25	1.069	1.081	1.070	1.081
21.50	0.788	0.872	0.776	0.853	32.50	1.110	1.123	1.083	1.102	43.50	1.071	1.083	1.071	1.082
21.75	0.781	0.891	0.769	0.870	32.75	1.110	1.121	1.084	1.102	43.75	1.072	1.085	1.073	1.083
22.00	0.784	0.909	0.770	0.884	33.00	1.109	1.119	1.086	1.101	44.00	1.074	1.087	1.074	1.085
22.25	0.792	0.925	0.778	0.898	33.25	1.108	1.116	1.087	1.100	44.25	1.076	1.089	1.075	1.086
22.50	0.803	0.940	0.788	0.911	33.50	1.107	1.113	1.088	1.099	44.50	1.078	1.090	1.077	1.088
22.75	0.817	0.955	0.800	0.922	33.75	1.106	1.111	1.088	1.098	44.75	1.080	1.092	1.078	1.089
23.00	0.833	0.969	0.814	0.934	34.00	1.105	1.108	1.089	1.096	45.00	1.082	1.094	1.080	1.091
23.25	0.848	0.983	0.827	0.945	34.25	1.103	1.105	1.089	1.095	45.25	1.084	1.096	1.081	1.092
23.50	0.865	0.996	0.841	0.956	34.50	1.102	1.102	1.089	1.093	45.50	1.086	1.098	1.083	1.093
23.75	0.881	1.009	0.855	0.966	34.75	1.100	1.099	1.089	1.091	45.75	1.088	1.100	1.085	1.095
24.00	0.897	1.021	0.869	0.976	35.00	1.098	1.096	1.088	1.090	46.00	1.091	1.102	1.086	1.096
24.25	0.913	1.032	0.882	0.985	35.25	1.096	1.094	1.088	1.088	46.25	1.093	1.104	1.088	1.098
24.50	0.928	1.042	0.896	0.993	35.50	1.094	1.091	1.087	1.086	46.50	1.095	1.106	1.089	1.099
24.75	0.943	1.052	0.908	1.001	35.75	1.092	1.088	1.086	1.084	46.75	1.097	1.107	1.091	1.100
25.00	0.957	1.060	0.921	1.009	36.00	1.090	1.086	1.085	1.082	47.00	1.100	1.109	1.093	1.101
25.25	0.971	1.068	0.932	1.016	36.25	1.087	1.083	1.084	1.081	47.25	1.102	1.111	1.094	1.103
25.50	0.983	1.075	0.943	1.022	36.50	1.085	1.081	1.082	1.079	47.50	1.104	1.113	1.096	1.104
25.75	0.995	1.082	0.954	1.027	36.75	1.083	1.078	1.081	1.077	47.75	1.106	1.115	1.097	1.105
26.00	1.007	1.088	0.963	1.032	37.00	1.081	1.076	1.079	1.076	48.00	1.108	1.116	1.099	1.106
26.25	1.017	1.093	0.973	1.037	37.25	1.079	1.074	1.078	1.074	48.25	1.111	1.118	1.100	1.107
26.50	1.026	1.098	0.981	1.041	37.50	1.076	1.073	1.076	1.073	48.50	1.113	1.120	1.102	1.108
26.75	1.035	1.102	0.989	1.045	37.75	1.074	1.071	1.075	1.072	48.75	1.115	1.121	1.103	1.108
27.00	1.043	1.106	0.996	1.050	38.00	1.072	1.070	1.074	1.071	49.00	1.117	1.123	1.104	1.109
27.25	1.050	1.110	1.002	1.053	38.25	1.071	1.069	1.072	1.070	49.25	1.119	1.124	1.106	1.110
27.50	1.056	1.114	1.008	1.057	38.50	1.069	1.068	1.071	1.069	49.50	1.121	1.125	1.107	1.111
27.75	1.062	1.117	1.013	1.062	38.75	1.067	1.067	1.070	1.069	49.75	1.123	1.127	1.108	1.111
28.00	1.067	1.120	1.018	1.066	39.00	1.066	1.066	1.068	1.068	50.00	1.125	1.128	1.109	1.112
28.25	1.072	1.123	1.023	1.070	39.25	1.065	1.066	1.067	1.068					

Table S23: Theoretical Assymetric Parameter for $4b_2$ Orbital. Energy in eV.

E	SEP-L	SE-L	SEP-V	SE-V	E	SEP-L	SE-L	SEP-V	SE-V	E	SEP-L	SE-L	SEP-V	SE-V
18.25	-0.074	-0.056	-0.074	-0.022	29.00	0.778	0.745	0.728	0.693	39.75	0.760	0.812	0.740	0.793
18.50	-0.032	-0.005	-0.037	0.019	29.25	0.780	0.746	0.731	0.693	40.00	0.767	0.822	0.748	0.803
18.75	0.003	0.028	-0.005	0.039	29.50	0.782	0.745	0.733	0.693	40.25	0.774	0.833	0.755	0.814
19.00	0.033	0.025	0.022	0.023	29.75	0.784	0.744	0.735	0.693	40.50	0.782	0.844	0.764	0.824
19.25	0.059	-0.025	0.045	-0.041	30.00	0.785	0.743	0.736	0.692	40.75	0.791	0.854	0.772	0.835
19.50	0.077	-0.116	0.059	-0.145	30.25	0.786	0.742	0.737	0.691	41.00	0.799	0.865	0.781	0.846
19.75	0.086	-0.210	0.063	-0.244	30.50	0.786	0.740	0.737	0.690	41.25	0.808	0.876	0.791	0.857
20.00	0.082	-0.255	0.053	-0.289	30.75	0.785	0.738	0.737	0.688	41.50	0.818	0.887	0.801	0.867
20.25	0.063	-0.234	0.028	-0.265	31.00	0.784	0.735	0.736	0.686	41.75	0.827	0.897	0.811	0.878
20.50	0.031	-0.166	-0.010	-0.194	31.25	0.783	0.733	0.735	0.684	42.00	0.837	0.908	0.821	0.889
20.75	-0.010	-0.077	-0.055	-0.103	31.50	0.781	0.730	0.734	0.683	42.25	0.848	0.918	0.831	0.899
21.00	-0.050	0.016	-0.098	-0.009	31.75	0.779	0.728	0.732	0.681	42.50	0.858	0.929	0.841	0.909
21.25	-0.077	0.104	-0.123	0.079	32.00	0.777	0.725	0.730	0.679	42.75	0.868	0.939	0.852	0.919
21.50	-0.079	0.183	-0.122	0.158	32.25	0.774	0.722	0.728	0.677	43.00	0.879	0.949	0.862	0.929
21.75	-0.053	0.254	-0.092	0.228	32.50	0.771	0.719	0.726	0.675	43.25	0.889	0.959	0.873	0.939
22.00	-0.004	0.317	-0.039	0.290	32.75	0.768	0.717	0.723	0.674	43.50	0.900	0.969	0.883	0.949
22.25	0.060	0.372	0.028	0.343	33.00	0.765	0.715	0.720	0.672	43.75	0.910	0.978	0.893	0.958
22.50	0.131	0.420	0.101	0.389	33.25	0.762	0.712	0.718	0.671	44.00	0.921	0.987	0.903	0.967
22.75	0.203	0.462	0.174	0.430	33.50	0.759	0.710	0.715	0.670	44.25	0.931	0.996	0.914	0.976
23.00	0.271	0.499	0.243	0.464	33.75	0.755	0.708	0.713	0.669	44.50	0.941	1.005	0.924	0.984
23.25	0.335	0.531	0.307	0.494	34.00	0.752	0.707	0.710	0.669	44.75	0.951	1.014	0.933	0.993
23.50	0.392	0.558	0.364	0.520	34.25	0.749	0.706	0.708	0.668	45.00	0.961	1.022	0.943	1.001
23.75	0.444	0.583	0.414	0.542	34.50	0.746	0.705	0.705	0.669	45.25	0.971	1.030	0.952	1.009
24.00	0.490	0.603	0.459	0.560	34.75	0.743	0.705	0.703	0.669	45.50	0.980	1.038	0.962	1.017
24.25	0.531	0.622	0.498	0.577	35.00	0.740	0.705	0.701	0.671	45.75	0.990	1.046	0.971	1.024
24.50	0.566	0.637	0.532	0.591	35.25	0.737	0.705	0.700	0.672	46.00	0.999	1.054	0.980	1.031
24.75	0.598	0.650	0.562	0.604	35.50	0.735	0.706	0.698	0.674	46.25	1.008	1.061	0.988	1.038
25.00	0.625	0.663	0.588	0.614	35.75	0.732	0.708	0.697	0.677	46.50	1.016	1.068	0.997	1.045
25.25	0.649	0.673	0.610	0.623	36.00	0.730	0.710	0.696	0.680	46.75	1.025	1.075	1.005	1.052
25.50	0.669	0.681	0.629	0.631	36.25	0.729	0.713	0.695	0.684	47.00	1.033	1.081	1.013	1.058
25.75	0.687	0.689	0.645	0.638	36.50	0.728	0.716	0.695	0.688	47.25	1.041	1.088	1.020	1.064
26.00	0.702	0.697	0.659	0.644	36.75	0.727	0.720	0.696	0.693	47.50	1.049	1.094	1.028	1.070
26.25	0.715	0.703	0.671	0.650	37.00	0.727	0.725	0.696	0.699	47.75	1.057	1.100	1.035	1.076
26.50	0.726	0.709	0.681	0.656	37.25	0.727	0.730	0.698	0.705	48.00	1.064	1.105	1.042	1.082
26.75	0.735	0.715	0.689	0.662	37.50	0.727	0.736	0.699	0.712	48.25	1.071	1.111	1.049	1.087
27.00	0.743	0.720	0.696	0.667	37.75	0.729	0.742	0.701	0.719	48.50	1.078	1.116	1.056	1.092
27.25	0.750	0.726	0.702	0.672	38.00	0.730	0.750	0.704	0.727	48.75	1.085	1.122	1.062	1.097
27.50	0.756	0.730	0.707	0.677	38.25	0.733	0.757	0.708	0.735	49.00	1.091	1.127	1.069	1.102
27.75	0.761	0.734	0.712	0.681	38.50	0.736	0.765	0.712	0.744	49.25	1.098	1.132	1.075	1.107
28.00	0.765	0.738	0.716	0.684	38.75	0.740	0.774	0.716	0.753	49.50	1.104	1.136	1.081	1.111
28.25	0.769	0.741	0.719	0.687	39.00	0.744	0.783	0.721	0.762	49.75	1.110	1.141	1.086	1.116
28.50	0.772	0.743	0.723	0.690	39.25	0.749	0.792	0.727	0.772	50.00	1.116	1.145	1.092	1.120
28.75	0.775	0.745	0.726	0.692	39.50	0.754	0.802	0.733	0.782					

Table S24: Theoretical Assymetric Parameter for $6a_1$ Orbital. Energy in eV.

E	SEP-L	SE-L	SEP-V	SE-V	E	SEP-L	SE-L	SEP-V	SE-V	E	SEP-L	SE-L	SEP-V	SE-V
19.00	0.676	0.678	0.686	0.701	29.50	0.951	0.987	0.907	0.942	40.00	0.965	0.918	0.944	0.900
19.25	0.728	0.740	0.749	0.769	29.75	0.954	0.999	0.910	0.954	40.25	0.959	0.915	0.939	0.896
19.50	0.777	0.807	0.805	0.839	30.00	0.958	1.010	0.915	0.966	40.50	0.953	0.912	0.933	0.893
19.75	0.824	0.875	0.857	0.909	30.25	0.964	1.021	0.921	0.977	40.75	0.948	0.909	0.928	0.890
20.00	0.870	0.931	0.905	0.965	30.50	0.971	1.030	0.928	0.986	41.00	0.943	0.907	0.923	0.888
20.25	0.914	0.962	0.950	0.994	30.75	0.980	1.039	0.937	0.995	41.25	0.938	0.905	0.919	0.886
20.50	0.953	0.960	0.990	0.989	31.00	0.989	1.046	0.947	1.003	41.50	0.934	0.903	0.915	0.884
20.75	0.988	0.936	1.024	0.959	31.25	0.999	1.052	0.957	1.010	41.75	0.930	0.902	0.911	0.883
21.00	1.015	0.905	1.049	0.925	31.50	1.009	1.058	0.968	1.016	42.00	0.927	0.901	0.907	0.881
21.25	1.031	0.880	1.062	0.895	31.75	1.019	1.062	0.978	1.021	42.25	0.924	0.901	0.904	0.881
21.50	1.032	0.862	1.059	0.873	32.00	1.029	1.065	0.988	1.024	42.50	0.921	0.900	0.901	0.880
21.75	1.019	0.852	1.041	0.858	32.25	1.038	1.067	0.997	1.027	42.75	0.918	0.900	0.899	0.880
22.00	0.994	0.847	1.010	0.850	32.50	1.046	1.068	1.006	1.029	43.00	0.916	0.901	0.897	0.880
22.25	0.961	0.847	0.972	0.846	32.75	1.053	1.068	1.013	1.030	43.25	0.915	0.901	0.895	0.880
22.50	0.928	0.850	0.934	0.845	33.00	1.060	1.067	1.020	1.030	43.50	0.914	0.902	0.893	0.881
22.75	0.900	0.854	0.901	0.846	33.25	1.065	1.065	1.026	1.029	43.75	0.913	0.903	0.892	0.881
23.00	0.879	0.858	0.876	0.848	33.50	1.069	1.063	1.030	1.028	44.00	0.912	0.905	0.891	0.882
23.25	0.865	0.864	0.858	0.849	33.75	1.072	1.060	1.034	1.026	44.25	0.912	0.907	0.891	0.884
23.50	0.856	0.869	0.847	0.852	34.00	1.074	1.057	1.037	1.023	44.50	0.912	0.908	0.890	0.885
23.75	0.852	0.875	0.840	0.854	34.25	1.075	1.053	1.039	1.020	44.75	0.912	0.910	0.890	0.887
24.00	0.852	0.882	0.837	0.857	34.50	1.076	1.048	1.040	1.017	45.00	0.913	0.913	0.890	0.889
24.25	0.854	0.888	0.836	0.861	34.75	1.075	1.043	1.040	1.013	45.25	0.914	0.915	0.891	0.891
24.50	0.857	0.895	0.837	0.864	35.00	1.074	1.038	1.040	1.008	45.50	0.915	0.918	0.892	0.893
24.75	0.862	0.902	0.839	0.868	35.25	1.072	1.032	1.039	1.003	45.75	0.917	0.921	0.893	0.895
25.00	0.868	0.909	0.842	0.873	35.50	1.070	1.026	1.037	0.998	46.00	0.918	0.924	0.894	0.898
25.25	0.874	0.915	0.846	0.877	35.75	1.066	1.020	1.034	0.993	46.25	0.920	0.927	0.896	0.901
25.50	0.881	0.921	0.851	0.880	36.00	1.063	1.013	1.031	0.987	46.50	0.923	0.931	0.897	0.904
25.75	0.888	0.926	0.856	0.883	36.25	1.058	1.006	1.028	0.981	46.75	0.925	0.934	0.899	0.907
26.00	0.895	0.930	0.861	0.886	36.50	1.054	0.999	1.024	0.975	47.00	0.928	0.938	0.901	0.911
26.25	0.903	0.933	0.867	0.887	36.75	1.048	0.992	1.020	0.969	47.25	0.930	0.942	0.904	0.914
26.50	0.910	0.935	0.872	0.888	37.00	1.043	0.986	1.015	0.963	47.50	0.933	0.946	0.906	0.918
26.75	0.917	0.935	0.877	0.888	37.25	1.037	0.979	1.010	0.956	47.75	0.936	0.950	0.909	0.921
27.00	0.924	0.935	0.883	0.887	37.50	1.031	0.972	1.004	0.950	48.00	0.940	0.954	0.912	0.925
27.25	0.929	0.935	0.887	0.887	37.75	1.024	0.965	0.999	0.944	48.25	0.943	0.958	0.915	0.929
27.50	0.935	0.935	0.891	0.887	38.00	1.018	0.959	0.993	0.938	48.50	0.947	0.963	0.918	0.933
27.75	0.939	0.936	0.895	0.888	38.25	1.011	0.953	0.987	0.932	48.75	0.951	0.967	0.922	0.937
28.00	0.942	0.938	0.897	0.890	38.50	1.004	0.947	0.981	0.927	49.00	0.955	0.971	0.925	0.941
28.25	0.944	0.942	0.899	0.895	38.75	0.998	0.941	0.975	0.922	49.25	0.959	0.976	0.929	0.945
28.50	0.946	0.948	0.901	0.901	39.00	0.991	0.936	0.968	0.917	49.50	0.963	0.980	0.933	0.950
28.75	0.947	0.955	0.902	0.909	39.25	0.984	0.931	0.962	0.912	49.75	0.967	0.984	0.937	0.954
29.00	0.948	0.965	0.903	0.919	39.50	0.978	0.926	0.956	0.907	50.00	0.971	0.989	0.941	0.958
29.25	0.950	0.976	0.905	0.931	39.75	0.971	0.922	0.950	0.903					

Table S25: Theoretical Assymetric Parameter for $3b_2$ Orbital. Energy in eV.

E	SEP-L	SE-L	SEP-V	SE-V	E	SEP-L	SE-L	SEP-V	SE-V	E	SEP-L	SE-L	SEP-V	SE-V
22.50	0.661	0.686	0.694	0.715	31.75	0.603	0.590	0.600	0.592	41.00	1.144	1.184	1.138	1.176
22.75	0.732	0.750	0.746	0.762	32.00	0.604	0.608	0.601	0.609	41.25	1.152	1.189	1.146	1.181
23.00	0.777	0.774	0.775	0.767	32.25	0.605	0.629	0.603	0.629	41.50	1.159	1.193	1.153	1.185
23.25	0.806	0.748	0.791	0.716	32.50	0.607	0.653	0.605	0.652	41.75	1.166	1.197	1.160	1.189
23.50	0.823	0.658	0.796	0.600	32.75	0.611	0.679	0.610	0.677	42.00	1.172	1.201	1.167	1.192
23.75	0.827	0.522	0.790	0.444	33.00	0.618	0.705	0.617	0.702	42.25	1.178	1.204	1.172	1.195
24.00	0.819	0.419	0.771	0.335	33.25	0.629	0.732	0.628	0.728	42.50	1.184	1.206	1.178	1.198
24.25	0.797	0.383	0.738	0.302	33.50	0.644	0.758	0.642	0.753	42.75	1.189	1.208	1.183	1.200
24.50	0.759	0.387	0.688	0.309	33.75	0.661	0.783	0.658	0.778	43.00	1.193	1.210	1.187	1.202
24.75	0.705	0.400	0.624	0.326	34.00	0.681	0.808	0.678	0.802	43.25	1.197	1.212	1.191	1.203
25.00	0.641	0.410	0.552	0.340	34.25	0.704	0.832	0.699	0.826	43.50	1.201	1.213	1.195	1.204
25.25	0.578	0.415	0.484	0.348	34.50	0.727	0.855	0.722	0.848	43.75	1.204	1.214	1.198	1.205
25.50	0.526	0.414	0.433	0.351	34.75	0.751	0.877	0.745	0.870	44.00	1.207	1.214	1.201	1.205
25.75	0.492	0.411	0.402	0.352	35.00	0.776	0.898	0.769	0.891	44.25	1.209	1.214	1.203	1.205
26.00	0.471	0.408	0.387	0.353	35.25	0.799	0.918	0.792	0.910	44.50	1.211	1.214	1.205	1.205
26.25	0.457	0.408	0.379	0.358	35.50	0.823	0.937	0.815	0.930	44.75	1.213	1.214	1.207	1.205
26.50	0.446	0.411	0.374	0.366	35.75	0.845	0.955	0.837	0.948	45.00	1.214	1.213	1.208	1.205
26.75	0.436	0.419	0.369	0.379	36.00	0.867	0.973	0.859	0.965	45.25	1.215	1.213	1.209	1.204
27.00	0.427	0.431	0.365	0.397	36.25	0.888	0.990	0.880	0.982	45.50	1.216	1.212	1.210	1.203
27.25	0.420	0.447	0.363	0.417	36.50	0.908	1.005	0.900	0.998	45.75	1.217	1.211	1.210	1.203
27.50	0.415	0.465	0.364	0.440	36.75	0.927	1.021	0.919	1.013	46.00	1.217	1.210	1.210	1.202
27.75	0.416	0.484	0.370	0.462	37.00	0.945	1.035	0.937	1.027	46.25	1.217	1.208	1.210	1.200
28.00	0.421	0.502	0.380	0.484	37.25	0.963	1.049	0.955	1.041	46.50	1.217	1.207	1.210	1.199
28.25	0.430	0.519	0.395	0.505	37.50	0.979	1.062	0.971	1.054	46.75	1.216	1.206	1.210	1.198
28.50	0.444	0.534	0.413	0.522	37.75	0.995	1.074	0.987	1.067	47.00	1.216	1.204	1.209	1.197
28.75	0.461	0.546	0.434	0.537	38.00	1.010	1.086	1.003	1.078	47.25	1.215	1.203	1.209	1.196
29.00	0.481	0.556	0.457	0.549	38.25	1.025	1.097	1.017	1.090	47.50	1.214	1.202	1.208	1.195
29.25	0.500	0.562	0.480	0.558	38.50	1.038	1.108	1.031	1.100	47.75	1.213	1.200	1.207	1.193
29.50	0.520	0.566	0.502	0.563	38.75	1.051	1.118	1.044	1.110	48.00	1.212	1.199	1.206	1.192
29.75	0.538	0.568	0.522	0.566	39.00	1.064	1.127	1.057	1.120	48.25	1.211	1.197	1.205	1.191
30.00	0.555	0.567	0.541	0.567	39.25	1.076	1.136	1.069	1.129	48.50	1.210	1.196	1.205	1.190
30.25	0.569	0.565	0.557	0.566	39.50	1.087	1.145	1.081	1.137	48.75	1.209	1.195	1.204	1.189
30.50	0.580	0.563	0.570	0.565	39.75	1.098	1.153	1.092	1.145	49.00	1.207	1.193	1.203	1.188
30.75	0.589	0.562	0.581	0.564	40.00	1.108	1.160	1.102	1.152	49.25	1.206	1.192	1.202	1.187
31.00	0.596	0.563	0.588	0.566	40.25	1.118	1.167	1.112	1.159	49.50	1.205	1.191	1.201	1.187
31.25	0.600	0.567	0.594	0.570	40.50	1.127	1.173	1.121	1.165	49.75	1.204	1.190	1.200	1.186
31.50	0.602	0.576	0.597	0.579	40.75	1.136	1.179	1.130	1.171	50.00	1.203	1.189	1.199	1.185

Table S26: Theoretical Assymetric Parameter for $5a_1$ Orbital. Energy in eV.

E	SEP-L	SE-L	SEP-V	SE-V	E	SEP-L	SE-L	SEP-V	SE-V	E	SEP-L	SE-L	SEP-V	SE-V
24.00	0.677	0.648	0.728	0.690	32.75	0.860	0.863	0.802	0.812	41.50	1.195	1.215	1.177	1.199
24.25	0.745	0.711	0.793	0.757	33.00	0.866	0.875	0.807	0.825	41.75	1.200	1.219	1.183	1.204
24.50	0.796	0.772	0.839	0.818	33.25	0.870	0.889	0.812	0.840	42.00	1.204	1.223	1.188	1.208
24.75	0.839	0.845	0.877	0.889	33.50	0.875	0.905	0.817	0.857	42.25	1.209	1.226	1.193	1.212
25.00	0.876	0.927	0.909	0.960	33.75	0.879	0.922	0.822	0.875	42.50	1.213	1.230	1.198	1.216
25.25	0.909	0.977	0.937	0.990	34.00	0.884	0.940	0.828	0.895	42.75	1.217	1.233	1.203	1.220
25.50	0.940	0.977	0.963	0.973	34.25	0.891	0.958	0.836	0.914	43.00	1.221	1.236	1.208	1.223
25.75	0.970	0.950	0.988	0.937	34.50	0.900	0.975	0.847	0.932	43.25	1.225	1.238	1.212	1.226
26.00	0.996	0.913	1.009	0.896	34.75	0.912	0.992	0.860	0.950	43.50	1.228	1.241	1.216	1.229
26.25	1.018	0.876	1.022	0.857	35.00	0.925	1.008	0.875	0.967	43.75	1.232	1.243	1.220	1.232
26.50	1.027	0.841	1.021	0.820	35.25	0.940	1.023	0.892	0.983	44.00	1.235	1.245	1.224	1.234
26.75	1.020	0.809	1.002	0.787	35.50	0.957	1.037	0.909	0.998	44.25	1.238	1.246	1.228	1.236
27.00	0.997	0.781	0.970	0.757	35.75	0.973	1.050	0.928	1.013	44.50	1.241	1.248	1.231	1.238
27.25	0.966	0.756	0.933	0.731	36.00	0.990	1.062	0.946	1.026	44.75	1.243	1.249	1.234	1.240
27.50	0.931	0.736	0.894	0.709	36.25	1.006	1.073	0.964	1.039	45.00	1.246	1.250	1.237	1.241
27.75	0.895	0.721	0.856	0.692	36.50	1.022	1.084	0.981	1.051	45.25	1.248	1.251	1.239	1.243
28.00	0.861	0.712	0.821	0.682	36.75	1.037	1.094	0.997	1.062	45.50	1.250	1.252	1.242	1.244
28.25	0.830	0.709	0.788	0.677	37.00	1.051	1.104	1.013	1.072	45.75	1.252	1.252	1.244	1.245
28.50	0.802	0.713	0.759	0.679	37.25	1.064	1.113	1.027	1.082	46.00	1.253	1.253	1.246	1.245
28.75	0.777	0.721	0.734	0.686	37.50	1.076	1.121	1.041	1.092	46.25	1.255	1.253	1.248	1.246
29.00	0.758	0.734	0.714	0.696	37.75	1.087	1.129	1.053	1.101	46.50	1.256	1.253	1.249	1.246
29.25	0.745	0.748	0.699	0.709	38.00	1.098	1.137	1.065	1.110	46.75	1.257	1.253	1.251	1.247
29.50	0.737	0.763	0.690	0.723	38.25	1.108	1.144	1.076	1.118	47.00	1.258	1.253	1.252	1.247
29.75	0.734	0.779	0.687	0.737	38.50	1.117	1.151	1.087	1.126	47.25	1.258	1.252	1.253	1.247
30.00	0.737	0.793	0.689	0.749	38.75	1.126	1.158	1.097	1.133	47.50	1.259	1.252	1.254	1.247
30.25	0.745	0.806	0.695	0.761	39.00	1.134	1.164	1.106	1.141	47.75	1.259	1.252	1.254	1.247
30.50	0.756	0.817	0.705	0.770	39.25	1.141	1.170	1.115	1.147	48.00	1.259	1.251	1.255	1.247
30.75	0.768	0.826	0.716	0.778	39.50	1.148	1.176	1.123	1.154	48.25	1.259	1.250	1.255	1.246
31.00	0.782	0.833	0.729	0.784	39.75	1.155	1.181	1.131	1.161	48.50	1.259	1.250	1.256	1.246
31.25	0.797	0.838	0.743	0.788	40.00	1.162	1.187	1.139	1.167	48.75	1.259	1.249	1.256	1.246
31.50	0.810	0.842	0.756	0.790	40.25	1.168	1.192	1.146	1.173	49.00	1.259	1.248	1.256	1.245
31.75	0.823	0.844	0.767	0.792	40.50	1.174	1.197	1.153	1.178	49.25	1.259	1.248	1.256	1.245
32.00	0.835	0.847	0.778	0.795	40.75	1.179	1.202	1.159	1.184	49.50	1.258	1.247	1.256	1.245
32.25	0.845	0.850	0.787	0.798	41.00	1.185	1.206	1.165	1.189	49.75	1.258	1.246	1.256	1.244
32.50	0.853	0.855	0.795	0.804	41.25	1.190	1.211	1.171	1.194					

Table S27: Theoretical Assymetric Parameter for $4a_1$ Orbital. Energy in eV.

E	SEP-L	SE-L	SEP-V	SE-V	E	SEP-L	SE-L	SEP-V	SE-V	E	SEP-L	SE-L	SEP-V	SE-V
29.75	0.603	0.664	0.543	0.616	36.75	0.785	0.735	0.784	0.730	43.75	1.058	1.062	1.031	1.036
30.00	0.560	0.754	0.456	0.700	37.00	0.797	0.753	0.795	0.748	44.00	1.061	1.067	1.033	1.039
30.25	0.293	0.811	0.176	0.753	37.25	0.809	0.769	0.806	0.764	44.25	1.064	1.071	1.036	1.043
30.50	0.289	0.845	0.216	0.783	37.50	0.821	0.785	0.818	0.780	44.50	1.067	1.074	1.038	1.046
30.75	0.487	0.858	0.423	0.788	37.75	0.834	0.799	0.830	0.794	44.75	1.069	1.077	1.040	1.048
31.00	0.598	0.843	0.543	0.757	38.00	0.848	0.813	0.843	0.807	45.00	1.072	1.081	1.043	1.051
31.25	0.638	0.785	0.587	0.674	38.25	0.863	0.826	0.857	0.820	45.25	1.074	1.083	1.045	1.054
31.50	0.643	0.674	0.595	0.542	38.50	0.879	0.839	0.872	0.832	45.50	1.077	1.086	1.048	1.056
31.75	0.632	0.556	0.590	0.429	38.75	0.895	0.851	0.888	0.843	45.75	1.079	1.089	1.051	1.058
32.00	0.618	0.522	0.579	0.421	39.00	0.911	0.863	0.903	0.855	46.00	1.082	1.091	1.053	1.061
32.25	0.603	0.564	0.567	0.485	39.25	0.927	0.875	0.918	0.866	46.25	1.085	1.094	1.056	1.063
32.50	0.589	0.613	0.557	0.549	39.50	0.942	0.888	0.932	0.878	46.50	1.088	1.096	1.059	1.066
32.75	0.578	0.642	0.549	0.587	39.75	0.956	0.901	0.945	0.890	46.75	1.091	1.099	1.063	1.068
33.00	0.572	0.650	0.546	0.602	40.00	0.969	0.915	0.956	0.903	47.00	1.094	1.101	1.066	1.071
33.25	0.571	0.645	0.549	0.603	40.25	0.980	0.929	0.966	0.916	47.25	1.097	1.104	1.069	1.073
33.50	0.576	0.635	0.557	0.597	40.50	0.991	0.943	0.975	0.929	47.50	1.100	1.106	1.073	1.076
33.75	0.587	0.623	0.570	0.590	40.75	1.000	0.957	0.983	0.942	47.75	1.103	1.109	1.076	1.079
34.00	0.601	0.613	0.587	0.584	41.00	1.008	0.970	0.990	0.954	48.00	1.106	1.112	1.080	1.082
34.25	0.619	0.607	0.608	0.581	41.25	1.015	0.983	0.996	0.966	48.25	1.110	1.115	1.084	1.085
34.50	0.639	0.605	0.630	0.582	41.50	1.022	0.995	1.002	0.977	48.50	1.113	1.117	1.088	1.088
34.75	0.659	0.607	0.652	0.588	41.75	1.027	1.006	1.006	0.987	48.75	1.117	1.120	1.092	1.091
35.00	0.679	0.615	0.674	0.598	42.00	1.033	1.016	1.010	0.996	49.00	1.120	1.123	1.096	1.095
35.25	0.698	0.627	0.694	0.612	42.25	1.037	1.025	1.014	1.003	49.25	1.124	1.126	1.099	1.098
35.50	0.716	0.642	0.713	0.630	42.50	1.042	1.033	1.018	1.011	49.50	1.127	1.129	1.103	1.102
35.75	0.732	0.660	0.730	0.649	42.75	1.046	1.040	1.021	1.017	49.75	1.131	1.132	1.107	1.105
36.00	0.747	0.679	0.745	0.670	43.00	1.049	1.047	1.023	1.022	50.00	1.134	1.136	1.111	1.109
36.25	0.761	0.698	0.759	0.690	43.25	1.052	1.052	1.026	1.027					
36.50	0.773	0.717	0.772	0.710	43.50	1.056	1.058	1.029	1.032					
